# p53 suppresses lipid droplet–fueled tumorigenesis through phosphatidylcholine

**DOI:** 10.1172/JCI171788

**Published:** 2024-01-09

**Authors:** Xiuduan Xu, Jianqin Wang, Li Xu, Peng Li, Peng Jiang

**Affiliations:** 1 State Key Laboratory of Molecular Oncology, School of Life Sciences, Tsinghua University, and Tsinghua-Peking Center for Life Sciences, Beijing, China.; 2 School of Life Sciences, Tsinghua University, and Tsinghua-Peking Center for Life Sciences, Beijing, China.; 3 Tianjian Laboratory of Advanced Biomedical Sciences, Zhengzhou University, Zhengzhou, China.

**Keywords:** Cell Biology, Cancer, Cholesterol, Tumor suppressors

## Abstract

Choline deficiency causes disorders including hepatic abnormalities and is associated with an increased risk of multiple types of cancer. Here, by choline-free diet–associated RNA-Seq analyses, we found that the tumor suppressor p53 drives the Kennedy pathway via PCYT1B to control the growth of lipid droplets (LDs) and their fueling role in tumorigenesis. Mechanistically, through upregulation of PCYT1B, p53 channeled depleted choline stores to phosphatidylcholine (PC) biosynthesis during choline starvation, thus preventing LD coalescence. Cells lacking p53 failed to complete this response to choline depletion, leading to hepatic steatosis and tumorigenesis, and these effects could be reversed by enforcement of PCYT1B expression or restoration of PC abundance. Furthermore, loss of p53 or defects in the Kennedy pathway increased surface localization of hormone-sensitive lipase on LDs to release specific fatty acids that fueled tumor cells in vivo and in vitro. Thus, p53 loss leads to dysregulation of choline metabolism and LD growth and couples perturbed LD homeostasis to tumorigenesis.

## Abstract 2

<p>p53 loss drives lipid droplet growth that fuels tumorigenesis.</p>

## Graphical abstract

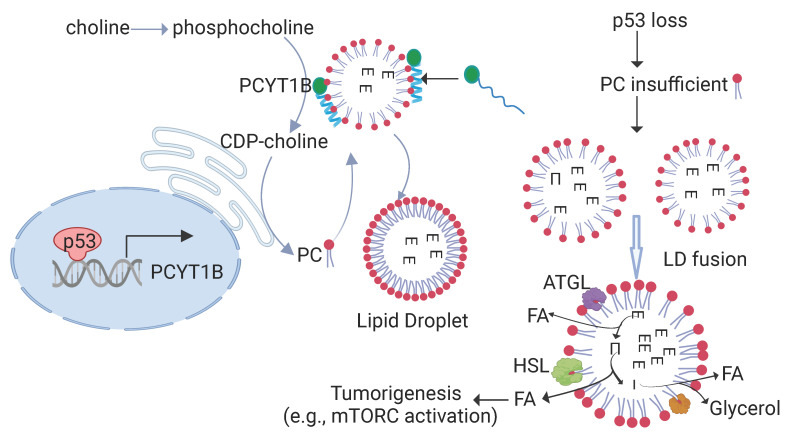

## Introduction

Choline deficiency, a cause of hepatic abnormalities, is associated with greater risk of multiple cancers (1, 2). Lipid droplets (LDs) serve as important organelles that primarily package neutral lipids, such as sterol esters or triglyceride (TG), for metabolic energy and membrane precursors with phospholipid monolayers decorated by LD-specific proteins (3, 4). In mammalian LDs, phosphatidylcholine (PC) is the major surface phospholipid and plays a crucial role in emulsifying LDs and regulating LD coalescence or fusion (5, 6). The main pathway for the synthesis of PC is the Kennedy (CDP-choline) pathway (Supplemental Figure 3B; supplemental material available online with this article; https://doi.org/10.1172/JCI171788DS1) (7), which is conserved from yeast to humans (8). LDs provide neutral lipid to lipoproteins for their maturation and secretion. In hepatocytes, apolipoprotein B lipidation is critical for very low-density lipoprotein (VLDL) maturation and secretion, which directly controls plasma levels of TG and cholesterol. In addition to VLDL secretion, lipolysis also contributes to LD-mediated mobilization of neutral lipids. Hormone-sensitive lipase (HSL) is a key enzyme in the mobilization of fatty acids from acylglycerols and can translocate from the cytosol to the surface of LDs when stimulated to release specific fatty acids. Some of the proteins, such as the diacylglycerol acyltransferase 2 (DGAT2) and CIDE proteins, have been shown as LD-binding proteins that are essential for LD expansion (6, 9–11). Moreover, the LD localization of PCYT1A or PCYT1B (also known as CCTα and CCTβ, respectively) allows for the local synthesis of sufficient PC to meet the demand for PC for LD surface expansion (5).

Choline is indispensable for many fundamental processes in the body, and its deficiency can cause reversible hepatic abnormalities in patients (12). Here, to elucidate how p53 loss confers a more profound fatty liver on mice in the absence of dietary choline, we performed RNA-sequencing (RNA-Seq) analysis and found that the Kennedy pathway is highly dysregulated by p53 loss, contributing to LD coalescence and tumorigenesis. Mechanistically, through transcriptional upregulation of PCYT1B expression, p53 efficiently channels depleted choline stores to PC synthesis during choline starvation, resulting in impaired LD coalescence and hepatic steatosis. Moreover, reduced PCYT1B expression or suppressed PC synthesis due to p53 loss increases the surface localization of HSL on LDs to release specific fatty acids as fuel for tumor cells in vitro and in vivo using different hepatocellular carcinoma mouse models.

## Results

### Choline-free diet–associated RNA-Seq reveals downregulation of the Kennedy pathway in fatty liver of p53-deficient mice.

**Figure 1:**
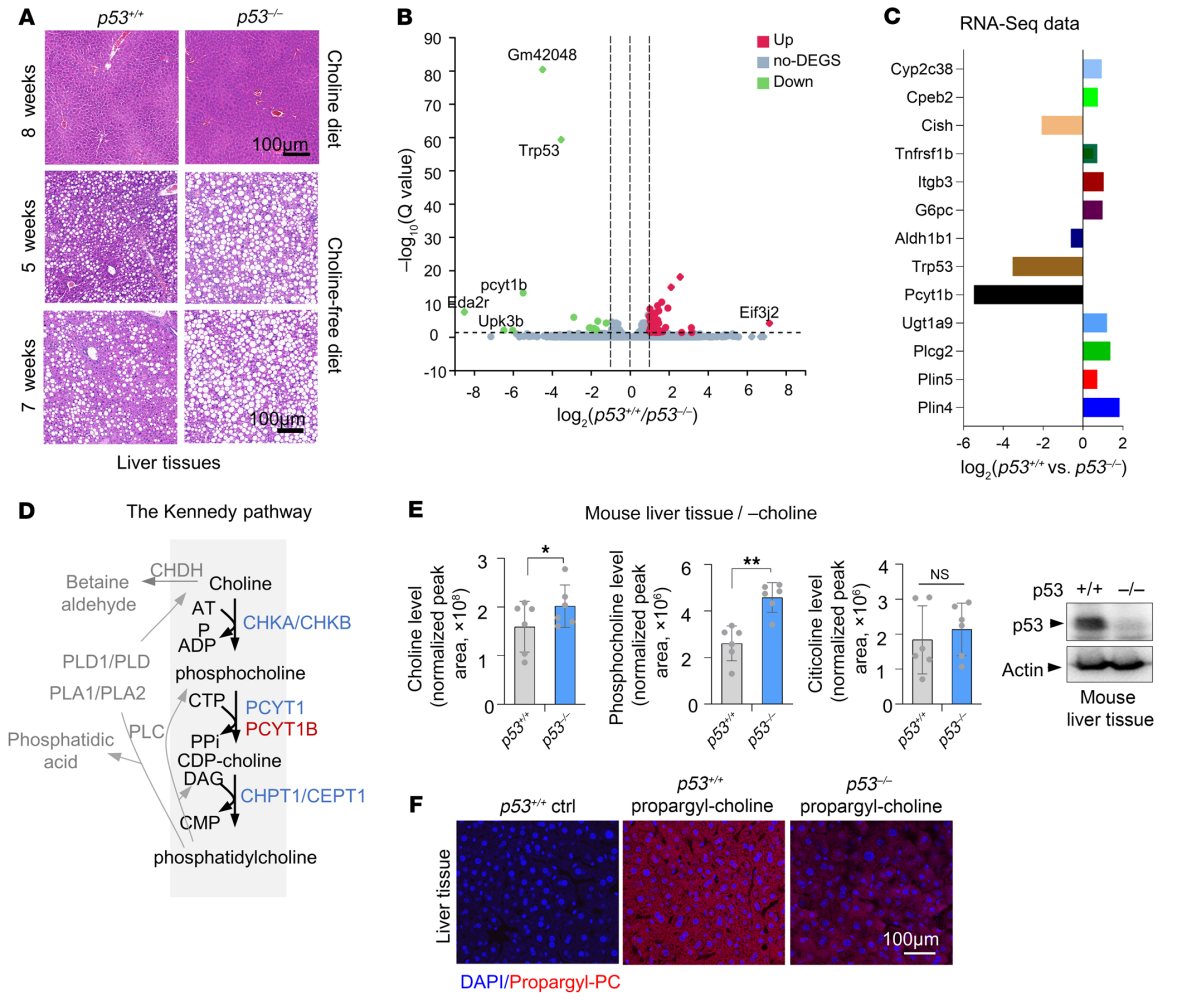
Choline-free diet–associated RNA-Seq analysis reveals that the Kennedy pathway is downregulated in fatty liver of p53-deficient mice. (**A**) *p53^+/+^* and *p53^–/–^* mice were maintained on a normal diet for 8 weeks or on a choline-free diet at different time points. Liver tissue from these mice was stained with H&E. *n* = 3 mice per group. Scale bars: 100 μm. (**B** and **C**) RNA-Seq of liver tissue from *p53^+/+^* and *p53^–/–^* mice maintained on a choline-free diet for 4 weeks. (**B**) Volcano plot of log_2_ fold change of gene expression in the liver tissues between *p53^+/+^* and *p53^–/–^* mice. Highly upregulated genes are labeled as red dots, whereas downregulated ones are labeled in green. (**C**) Bar plot comparison of expression changes of genes involved in lipid metabolism by RNA-Seq analysis. *n* = 3 mice per group. (**D**) Schematic depicting the Kennedy (CDP-choline) pathway. (**E**) Normalized liquid chromatography–mass spectrometry (LC-MS) peak areas of choline, phosphocholine, and citicoline in the livers of *p53^+/+^* and *p53^–/–^* mice maintained on a choline-deficient diet for 5 weeks (*n* = 5 mice per group). p53 expression in liver tissue was determined by Western blot analysis. (**F**) *p53^+/+^*and *p53^–/–^* mice maintained on a choline-free diet were injected in the tail vein with 0.05 mg/g propargyl-choline for 24 hours or left untreated. The liver tissues were collected and sectioned, and images were acquired on a confocal laser scanning microscope. Scale bar: 100 μm. All data are mean ± SD. *P* values were calculated by 2-tailed unpaired Student’s *t* test (**E**). *P < 0.05, **P < 0.01.

Choline deficiency is associated with numerous disorders, such as liver disease and atherosclerosis (13, 14). Here we happened to find that *p53^–/–^* mice showed robust sensitivity to choline starvation compared with *p53^+/+^* control mice. p53-deficient mice maintained on a choline-free diet exhibited more severe fatty liver (Figure 1A). To investigate how this happens, we did RNA-Seq analysis and compared gene expression profiles in the livers of *p53^+/+^* and *p53^–/–^* mice fed a choline-free diet. Interestingly, the expression levels of several genes, including *Eda2r*, *Eif3j2*, *Pcyt1b*, and *Upk3b*, were mostly changed (Figure 1B and Supplemental Figure 1A). As with the increased fatty liver induced by choline starvation in p53-deficient mice, we were particularly interested in genes involved in lipid metabolism. We further compared the expression of genes involved in the regulation of lipid metabolism and found that *Pcyt1b* was the most significantly altered gene, with a strong decrease in expression in the livers of *p53^–/–^* mice (Figure 1C). *Pcyt1b* encodes an enzyme that converts phosphocholine to CDP-choline and is a rate-limiting enzyme in the Kennedy pathway (Figure 1D). Therefore, we focused on this metabolic pathway. Analysis of metabolites in this pathway independently verified the decrease in PCYT1B in *p53^–/–^* mice. Liver tissues from *p53^–/–^* mice showed higher levels of choline and phosphocholine (Figure 1E), and reduced PC as evidenced by decreased levels of propargyl-PC in *p53^–/–^* mice receiving propargyl-labeled choline (Figure 1F), compared with those from wild-type (WT) control animals. These observations suggest that low levels of PCYT1B in *p53^–/–^* tissues limit the metabolic step it catalyzes, leading to an accumulation of upstream metabolites and a reduction in downstream PCs during choline restriction. Intriguingly, no changes in citicoline levels were observed (Figure 1E), which may be due to compensatory effects of other metabolic pathways. Together, these findings reveal a reduced Kennedy pathway in the fatty liver of p53-deficient mice in the absence of dietary choline.

### p53 drives the Kennedy pathway for de novo PC biosynthesis.

Similar to the findings in mice (Figure 1, E and F), metabolomics analysis revealed that p53 ablation in HepG2 cells resulted in an accumulation of cellular choline and phosphocholine and reduced levels of the downstream PC, while citicoline remained unchanged when cells were cultured in choline-free medium (Supplemental Figure 1, B and C), indicating a possible role for p53 in modulating the Kennedy pathway. Notably, treatment of cells or mice with choline minimized the difference in levels of PC in WT and p53-deficient situations (Supplemental Figure 1, D–F), even though supplying cells with choline led to overall elevation of cellular PC abundance (Supplemental Figure 1G), indicating that exogenous choline supplementation could compromise the effect of p53 on PC metabolism.

**Figure 2:**
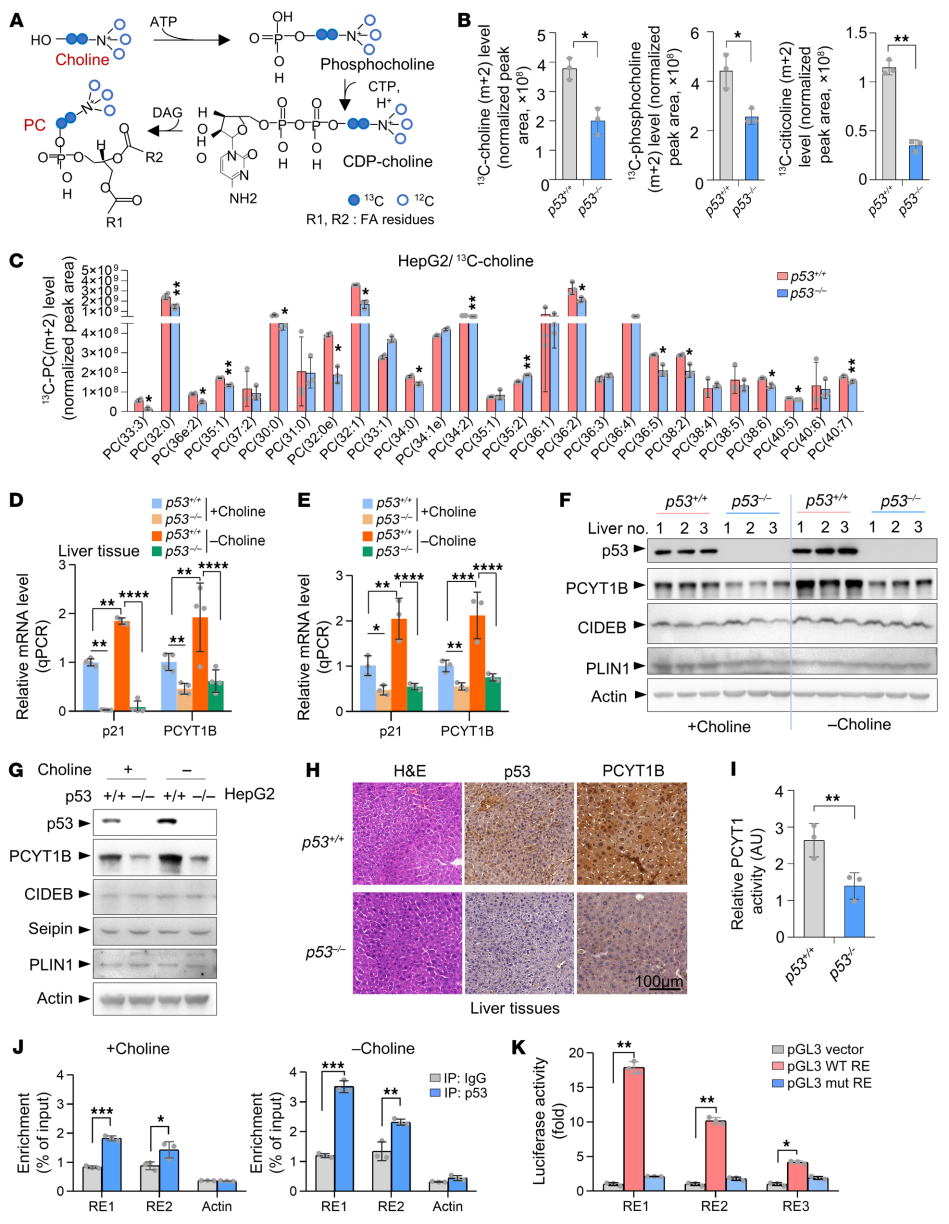
p53 drives the Kennedy pathway and transcriptionally upregulates PCYT1B expression. (**A**) Atom-transition map showing the isotope carbon-13 (^13^C) transfers from [1,2-^13^C_2_]choline through the PC synthetic pathway. Open circles represent carbon-12 (^12^C); blue circles indicate ^13^C from [1,2-^13^C_2_]choline. (**B** and **C**) Normalized peak areas of *m*+2 ^13^C-labeled metabolites from *p53^+/+^* and *p53^–/–^* HepG2 cells cultured with choline-free medium and pulse-labeled with [1,2-^13^C_2_]choline. (**B**) ^13^C-labeled choline, phosphocholine, and citicoline. (**C**) ^13^C-labeled PC. The isotopic labeling of each metabolite is denoted as *m* + *n*, where *n* is the number of ^13^C atoms. *n* = 3 samples per treatment. (**D** and **F**) Mice were fed a choline-deficient or normal diet for 4 weeks (*n* = 3 mice per group). Liver tissues were analyzed by quantitative reverse transcriptase PCR (RT-PCR) (**D**) and Western blot (**F**). (**E** and **G**) HepG2 cells cultured in choline-free medium or complete medium were analyzed by quantitative RT-PCR (**E**) and Western blot (**G**). (**H**) Immunohistochemistry of mouse liver tissues. Scale bar: 100 μm. (**I**) HepG2 cells were cultured with [1,2-^13^C_2_]choline for 30 minutes, and relative PCYT1 activity was determined. (**J**) Liver tissue from *p53^+/+^* mice fed a normal or choline-free diet was lysed and immunoprecipitated with anti-p53 or IgG antibody. The bound DNA was amplified by quantitative RT-PCR. (**K**) Luciferase constructs containing WT or mutant response elements (REs) were transfected into HEK93T cells together with FLAG-p53 or vector control. Renilla vector pRL-CMV was used as a transfection internal control. Relative luciferase activity was calculated by division of FLAG-p53 samples over the FLAG vector control samples. Data are mean ± SD. Each experiment was carried out at least 3 times. *P* values were calculated by 2-tailed unpaired Student’s *t* test (**B**, **C**, and **I**–**K**) or 2-way ANOVA with Tukey’s multiple-comparison test (**D** and **E**). *P < 0.05, **P < 0.01, ***P < 0.001, ****P < 0.0001.

We then performed a ^13^C metabolic flux analysis. By culturing cells with [1,2-^13^C_2_]choline, we observed that p53 loss resulted in a reduction in the synthesis of phosphocholine, citicoline, and PC from choline (Figure 2, A–C). Intriguingly, lower levels of ^13^C-labeled choline were found in *p53^–/–^* cells, raising a possibility that p53 might promote choline uptake (Figure 2B, left). Nevertheless, these data suggest that p53 deficiency impairs PC biosynthesis. In support of this, in vivo flux by treatment of mice with propargyl-labeled choline revealed significantly higher levels of propargyl-PC in liver tissue from *p53^+/+^* mice compared with *p53^–/–^* mice (Figure 1F). Similar results were also obtained in HepG2 cells (Supplemental Figure 1H).

The highly active PC synthesis within *p53^WT^* cells indicates that these cells might be susceptible to choline starvation. Indeed, choline depletion strongly dampened the survival of *p53^WT^* cells, while minimally affecting p53-deficient cells (Supplemental Figure 2, A and B). Notably, addition of exogenous choline or PC totally reversed this effect (Supplemental Figure 2, A and B). Similar findings were also found in the soft agar assays. Supplying cells with a choline-free medium resulted in a decreased anchorage-independent growth of *p53^+/+^*, but not *p53^–/–^*, HepG2 cells in soft agar, and either choline or PC supplementation completely restored the growth of *p53^+/+^* cells (Supplemental Figure 2, C and D). In agreement with the metabolic data (Supplemental Figure 1, C–E), no significant difference in the survival of *p53^+/+^* and *p53^–/–^* cells was observed when choline or PC was sufficient (Supplemental Figure 2E).

Collectively, these findings suggest that p53 positively regulates the Kennedy pathway and that cells with WT p53 are dependent on choline or PC for survival.

### p53 transcriptionally upregulates PCYT1B expression.

Next, we investigated how p53 regulates de novo PC biosynthesis. Consistent with the RNA-Seq data (Figure 1, B and C), loss of p53 was associated with decreased mRNA and protein levels of PCYT1B in mouse liver and HepG2 cells under both choline-deficient and choline-sufficient conditions (Figure 2, D–G). Moreover, results from immunohistochemistry showed that the protein expression of PCYT1B was higher in *p53^+/+^* murine livers and marginally expressed in liver tissues of *p53^–/–^* mice (Figure 2H). In line with these expression data, *p53^–/–^* cells displayed lower cellular PCYT1 activity than that in their WT counterparts (Figure 2I). Additionally, PCYT1B expression was lower in several tissues, though not heart and brain, in *p53^–/–^* mice than in *p53^+/+^* mice (Supplemental Figure 3A).

We further used chemical reagents to activate p53. Pharmacological activation of p53 with doxorubicin (DOX) boosted the expression of PCYT1B in *p53^+/+^* HepG2 cells but modestly boosted expression in *p53^–/–^* HepG2 cells (Supplemental Figure 3, B–D). By comparison, the expression of the other enzymes within these pathways was not dramatically changed (Supplemental Figure 3C). We also generated a tetracycline-controlled (Tet-On) inducible system and found that induction of p53 resulted in an increase in PCYT1B expression (Supplemental Figure 3, E and F). These findings were also supported by the observation that DOX, etoposide (ETO), or an MDM2 antagonist, nutlin-3, increased PCYT1B expression in a dose- and time-dependent manner in different types of cells (Supplemental Figure 4, A–I). Consistently, knockout of p53 downregulated the expression of PCYT1B, and the effect of nutlin-3 or ETO on PCYT1B expression was totally blocked when p53 was absent (Supplemental Figure 5, A–C). These findings indicate that regulation of PCYT1B is p53 dependent.

By analyzing the genomic sequences of the *PCYT1B* gene, we identified 2 putative p53 response elements in the mouse *Pcyt1b* gene and 3 putative p53 response elements in the human *PCYT1B* gene (Supplemental Figure 5, D and E). Chromatin immunoprecipitation assays revealed that endogenous p53 bound to the genomic regions containing these response elements, and this effect could be enhanced by choline starvation in mouse liver (Figure 2J). Similar results were obtained in HepG2 cells, where p53 occupancy of these response elements was found and DOX treatment strongly enhanced this effect (Supplemental Figure 5F). Likewise, FLAG-p53 was found to associate with the PCYT1B genomic DNA when expressed in HEK293 cells (Supplemental Figure 5G). In reporter assays, luciferase expression driven by genomic regions of PCYT1B containing these response elements (RE1, RE2, or RE3) increased luciferase expression in response to p53 introduction, whereas mutant response elements abolished this effect (Figure 2K).

p53 is the most frequently mutated gene in human cancer. To investigate whether PCYT1B expression can also be influenced by tumor-associated mutant p53, we knocked down p53 expression in MDA-MB-231 cells (carrying the p53^R280K^ mutation) and DU145 cells (heterozygous p53^P223L/V274F^) via siRNA interference. Unlike WT p53, mutant p53 ablation showed minimal effect on PCYT1B expression in these cell lines (Supplemental Figure 5, H and I). Furthermore, ectopic expression of mutant p53 (R175 or R273H) in p53-deficient HCT116 cells also did not significantly alter the expression of PCYT1B (Supplemental Figure 5, J and K).

### p53 impairs hepatic steatosis when choline is scarce.

**Figure 3:**
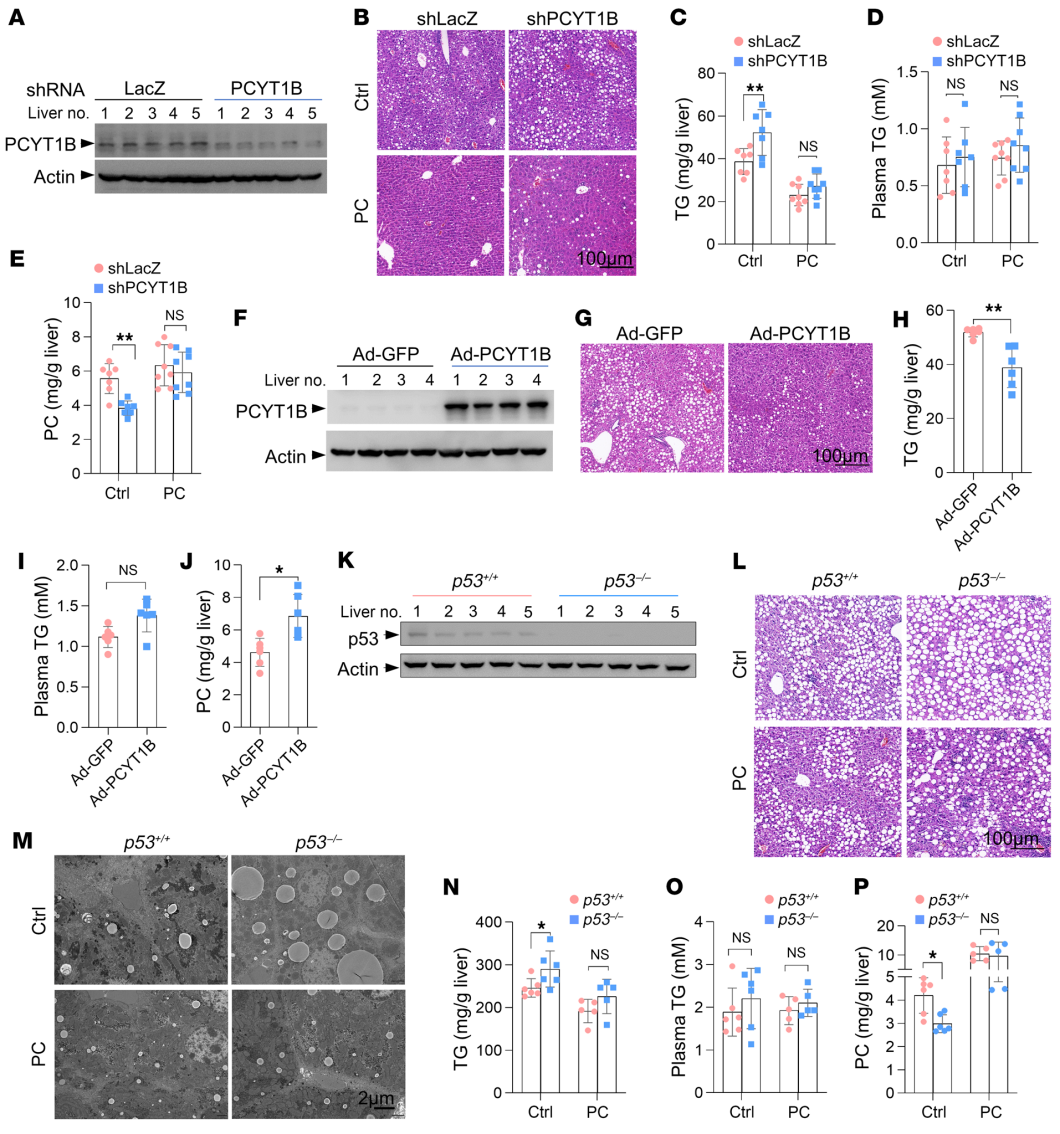
p53 suppresses hepatic steatosis via the PCYT1B/PC axis during choline starvation. (**A**) Mice to which shLacZ or shPCYT1B adenoviruses were administered were maintained on a choline-deficient diet, and liver PCYT1B expression was examined. (**B**–**E**) H&E staining (**B**), TG levels (**C**), and PC abundance (**E**) of liver tissues from mice given adenovirus and fed a choline-deficient diet supplemented or not supplemented with 300 μL PC (10 mg/mL). Plasma TGs were measured (**D**). *n* = 7 mice for control group; *n* = 8 mice for PC treatment. Scale bar: 100 μm. (**F**) PCYT1B expression in the liver of *p53^–/–^* mice given control (Ad-GFP) or Ad-PCYT1B virus and fed a choline-free diet. (**G**–**J**) *p53^–/–^* mice given control (Ad-GFP) or Ad-PCYT1B virus were maintained on a choline-free diet. Liver tissues were stained with H&E (**G**). TG (**H**) and PC abundance (**J**) in the livers and plasma TG (**I**) were measured. *n* = 6 mice per group. Scale bar: 100 μm. (**K**) p53 expression in livers of *p53^+/+^* and *p53^–/–^* mice maintained on a choline-free diet. (**L** and **M**) H&E staining (**L**) and transmission electron microscopy imaging (**M**) of liver tissues and levels of TG (**N**) and PC (**P**) in the liver and the plasma TG (**O**) from *p53^+/+^* and *p53^–/–^* mice maintained on a choline-free diet with or without 300 μL PC (10 mg/mL) orally daily. *n* = 6 mice for control group; *n* = 5 mice for PC treatment. Scale bars: 100 μm (**L**), 2 μm (**M**). All data are mean ± SD. *P* values were calculated by 2-tailed unpaired Student’s *t* test (**C**–**E**, **H**–**J**, and **N**–**P**). *P < 0.05, **P < 0.01.

Next, we considered the possibility that activation of the PCYT1B/PC axis by p53 might explain its role in suppressing hepatic steatosis. To this end, we generated liver-specific PCYT1B-knockdown mice (Figure 3A and Supplemental Figure 6A) and found that PCYT1B downregulation resulted in an increase in lipid accumulation in the livers and developed a fatty liver phenotype (Figure 3B). Consistently, higher content of intrahepatic TG, but not plasma TG, was observed in shPCYT1B mice compared with control shLacZ mice (Figure 3, C and D). Moreover, PCYT1B ablation led to a significant decline in PC levels in liver tissue, further demonstrating the physiological importance of PCYT1B for PC biosynthesis (Figure 3E), which also raised the possibility that PC may be essential for PCYT1B-mediated suppression of lipid accumulation in the liver. Indeed, restoration of PC levels by dietary PC strongly reversed the accumulation of lipid as well as TG induced by choline starvation in the liver and minimized the difference between control mice and PCYT1B-knockdown mice (Figure 3, B, C, and E).

Notably, PCYT1B knockdown did not affect animal body and liver weight (Supplemental Figure 6B). In agreement with the lipid accumulation data (Figure 3, B and C), shPCYT1B mice did to some extent show higher frequency of liver dysfunction (raised aspartate transaminase [AST], alanine transaminase [ALT], and alkaline phosphatase [ALP]), and this effect could be totally blocked by addition of PC (Supplemental Figure 6C). We also verified these findings by overexpression of PCYT1B in liver of *p53^–/–^* mice fed a choline-deficient diet (Figure 3F and Supplemental Figure 6D). In keeping with the knockdown data (Figure 3, A–E), overexpression of PCYT1B expression led to a reduction in contents of liver lipid and TG (not plasma TG) and correspondingly increased PC abundance (Figure 3, G–J, and Supplemental Figure 6E). By contrast, PCYT1B overexpression had no significant effect on body and liver weight or liver function (Supplemental Figure 6, F and G). These findings reveal that PCYT1B limits hepatic steatosis by supporting PC biosynthesis.

Additionally, immunohistochemical analysis showed that dietary administration of PC reduced the lipid content in the liver of *p53^–/–^* mice and narrowed the difference between *p53^+/+^* and *p53^–/–^* mice (Figure 3, K and L, and Supplemental Figure 7, A and B). Similar results were also obtained from transmission electron microscopy scanning analysis of liver tissues (Figure 3M). Analysis of TG levels showed that the increase in liver TG caused by p53 deficiency was abolished by PC administration, and these findings were further supported by the observations that intrahepatic PC in *p53^–/–^* mice was restored by dietary PC (Figure 3, N–P). In keeping with the PCYT1B-knockdown and overexpression data (Supplemental Figure 6), PC-supplemented diet did not change the weight of murine body and liver (Supplemental Figure 7, C and D). Like PCYT1B knockdown, p53 loss caused increased activity of AST, ALT, and ALP, which was completely abrogated by addition of PC (Supplemental Figure 7, E–G). Moreover, given that there is no difference in body weight of *p53^+/+^* and *p53^–/–^* mice on choline-free diet, p53 deficiency did not affect the body composition (Supplemental Figure 7, H and I). Additionally, levels of blood glucose were not changed between *p53^+/+^* and *p53^–/–^* mice (Supplemental Figure 7J). Again, along with the metabolic data in the situation of choline supplementation (Supplemental Figure 1, C–E), dietary choline–treated mice did not develop fatty liver, and there were no differences in liver dysfunction, body weight, or liver weight between *p53^+/+^* and *p53^–/–^* mice (Figure 1A and Supplemental Figure 7, K–N). Collectively, these findings suggest that upregulation of PCYT1B by p53 represses nonalcoholic fatty liver disease development via PC during choline starvation.

### p53 suppresses LD coalescence via PCYT1B-mediated PC synthesis during choline starvation.

**Figure 4:**
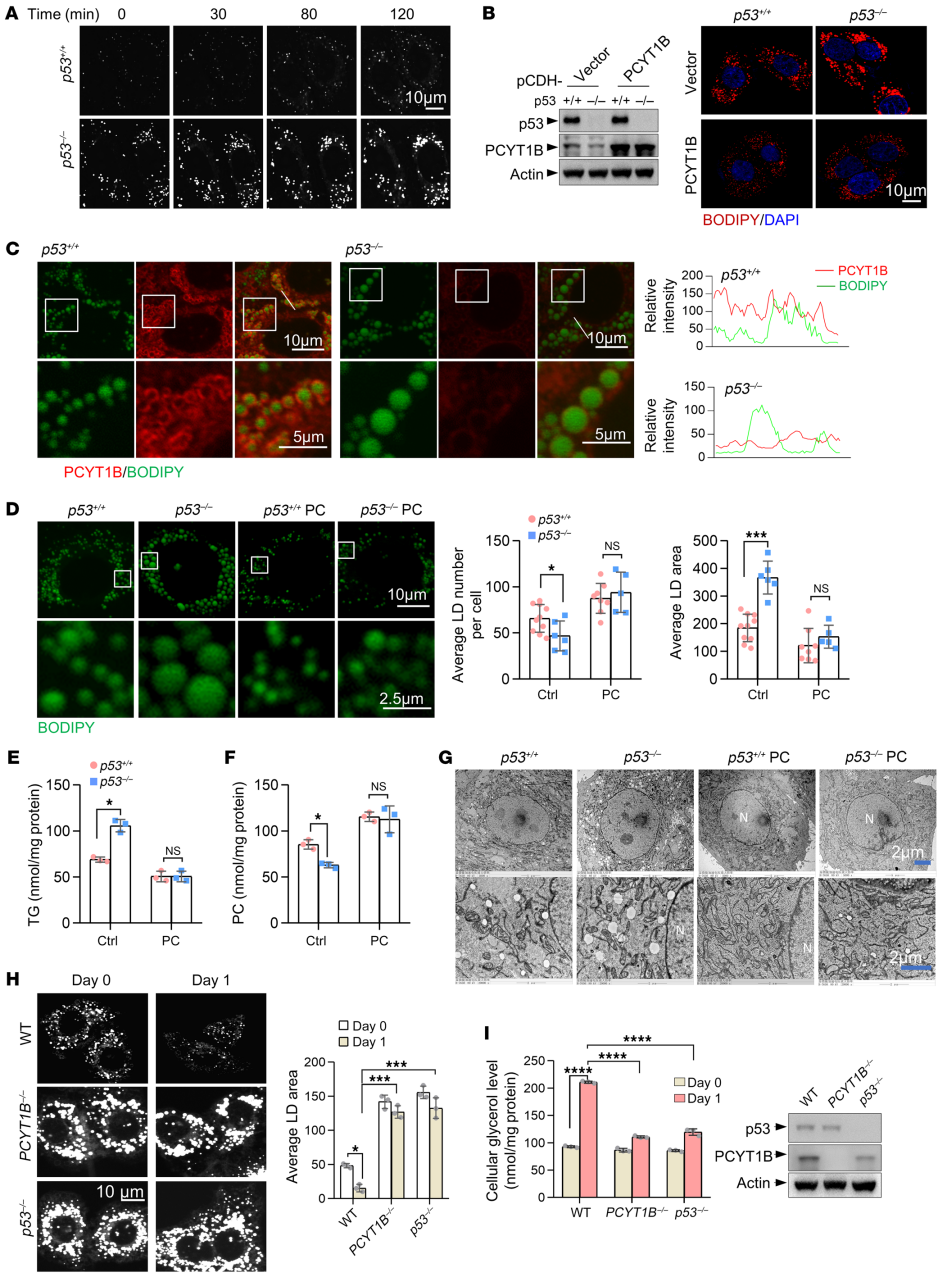
p53 regulates LD coalescence via PCYT1B-mediated PC synthesis. (**A**) HepG2 cells cultured with 200 μM oleate were stained with BODIPY and imaged at the indicated times. Scale bar: 10 μm. (**B**) HepG2 cells stably expressing PCYT1B or vector control were cultured with 200 μM oleate, stained with BODIPY dye and DAPI, and imaged with confocal microscopy. Expression of PCYT1B was determined by Western blot analysis. Scale bar: 10 μm. (**C**) Immunostaining of HepG2 cells loaded with 200 μM oleate for 12 hours. Enlarged areas are indicated, and the relative intensity of the fluorescence in the specified area of the overlapped images was analyzed (bottom panels). Scale bars: 10 μm (top), 5 μm (bottom). (**D**) *p53^+/+^* and *p53^–/–^* HepG2 cells cultured in choline-free medium supplemented or not supplemented with PC were loaded with 200 μM oleate and stained with BODIPY 493/503 dye. Average numbers and area of LDs were measured. Scale bars: 10 μm (top), 2.5 μm (bottom). (**E**–**G**) HepG2 cells were treated as in **D**. TG (**E**) and PC (**F**) levels were measured. LDs were imaged with transmission electron microscopy (**G**). N, nucleus. Scale bars: 2 μm. (**H**) WT control, *PCYT1B^–/–^*, and *p53^–/–^* HepG2 cells were loaded with 200 μM oleate for 1 day. Cells were then imaged by confocal microscopy after staining with BODIPY (day 0, left panels). Oleate was removed from the medium, and the cells were starved for 1 day in serum-free medium to induce lipolysis (day 1, right panels). Representative confocal midsections are shown. Scale bar: 10 μm. (**I**) Experiments were performed as in **H**, and cellular glycerol and protein expression was measured. All data are mean ± SD. Each experiment was carried out at least 3 independent times. *P* values were calculated by 2-tailed unpaired Student’s *t* test (**D**–**F**) or 2-way ANOVA followed by Tukey’s multiple-comparison test (**H** and **I**). *P < 0.05, ***P < 0.001, ****P < 0.0001.

LDs are the defining feature of hepatic steatosis and lipid storage (15). After treatment with oleate, *p53^–/–^* HepG2 cells displayed larger droplets than *p53^+/+^* cells when choline was deprived (Figure 4A). Moreover, by time-lapse confocal microscopy, we observed visible droplet coalescence and size growth in *p53^–/–^* cells, but not in *p53^+/+^* cells (Supplemental Videos 1 and 2), suggesting that p53 inhibits LD coalescence. To investigate whether this role of p53 is mediated by PCYT1B, we compared the size of LDs by BODIPY staining and TG levels in oleate-loaded cells treated with miltefosine, a PCYT1A/B inhibitor. Miltefosine treatment led to the formation of large droplets and minimized the difference between *p53^+/+^* and *p53^–/–^* cells (Supplemental Figure 8A). Given that PCYT1A is not regulated by p53 (Supplemental Figure 3C), these findings indicate that activation of PCYT1B contributes to p53-mediated suppression of growth in size of LDs. We also verified this by generating cell lines with stable overexpression of PCYT1B (Figure 4B, left). Forced expression of PCYT1B visually reduced cellular size of LDs, decreased TG levels, and correspondingly led to a significant increase in PC levels in both *p53^+/+^* and *p53^–/–^* cells when oleate was loaded (Figure 4B and Supplemental Figure 8B). Notably, overexpressed PCYT1B showed colocalization with LDs (Supplemental Figure 8C), and translocation of PCYT1B to the droplet surface is thought to be able to impede LD size by locally providing adequate PC (16). In line with this, we observed strong colocalization of endogenous PCYT1B and LDs in *p53^+/+^* cells, whereas larger LDs were observed in *p53^–/–^* cells with attenuated colocalization of PCYT1B due to reduced expression of PCYT1B (Figure 4C). Hence, p53 inhibits LD coalescence by upregulating surface levels of PCYT1B on LDs during choline starvation.

We further wanted to know whether the regulation of LD coalescence by p53 is PC dependent. p53 depletion led to significant reduction in PC levels in murine liver tissues (Figure 1F, Figure 2C, Figure 3P, and Supplemental Figure 8B) and oleate- or palmitic acid–loaded HepG2 cells (Supplemental Figure 9, A and B). Interestingly, addition of PC decreased LD size in both *p53^+/+^* and *p53^–/–^* cells loaded with oleate and abolished the difference between them (Figure 4D). These results were further verified by transfection of cells with liposomal PC (Supplemental Figure 9C). Consistent with the finding mentioned above that p53 suppresses LD coalescence, although the LD contents and TG contents increased when p53 was knocked out, the number of LDs declined in *p53^–/–^* cells (Figure 4, D–F). Notably, these phenomena were not observed when cells were supplied with PC (Figure 4, D–F). These findings indicate that increased PC synthesis is required for p53-mediated suppression of LD coalescence during choline starvation. To verify these findings and also to visualize and compare the size of LDs, we further used electron microscopy strategies. Similarly, *p53^–/–^* cells had larger LDs, and PC addition strongly reduced the size of LDs in both *p53^+/+^* and *p53^–/–^* cells loaded with oleate (Figure 4G). These findings were further verified by means of electron microscopic 3D reconstruction methods. *p53^–/–^* cells had larger LDs present, and this effect was blocked by PC treatment (Supplemental Figure 9D), reinforcing the notion that p53 regulates LD coalescence by modulating PCYT1B-mediated PC synthesis.

LDs provide lipid for VLDL secretion and fatty acids for oxidation. Therefore, lowering of either of these 2 events would expectedly cause LD growth (providing more surface area). However, levels of plasma TG in *p53^–/–^* mice maintained on a choline-free diet were not significantly different from those in WT mice (Figure 3O), and *p53^+/+^* and *p53^–/–^* mice treated with Triton WR-1339, a potent inhibitor of VLDL-TG catabolism (17), displayed comparable levels of plasma TG (Supplemental Figure 9E). Consistently, the secretion rates of apolipoprotein B-100 (apoB-100) and apoB-48 in the presence of Triton WR-1339 were similar between WT and *p53^–/–^* mice (Supplemental Figure 9F), and the expression of apoB in the murine livers was not affected by p53 knockout or PC treatment (Supplemental Figure 9G). Moreover, we did not observe any change in the activity of diacylglycerol *O*-acyltransferase 1 (DGAT1), which reflects the VLDL secretion and particle size (18) (Supplemental Figure 9H). Thus, it appears that regulation of LD size growth by p53 may not be due to VLDL secretion.

Also, we examined the effect of p53 and PCYT1B on lipolysis, which presumably can induce the breakup of larger droplets into smaller ones to provide more surface area for lipases (16, 19). By inducing LD formation followed by induction of lipid mobilization with serum-free medium lacking oleate, we found that WT control HepG2 cells had much fewer droplets, and by contrast, many droplets remained in *PCYT1B^–/–^* cells or *p53^–/–^* cells (Figure 4H). In line with this, highly increased levels of cellular glycerol, a production of lipolysis, were observed in WT control cells, not in the knockout cells (Figure 4I). These findings together indicate that p53 controls LD size by orchestrating LD coalescence and lipolysis via regulating PCYT1B-mediated PC biosynthesis.

### PCYT1B is a tumor suppressor and metabolic enzyme activity is required for its tumor-suppressive capacity.

**Figure 5:**
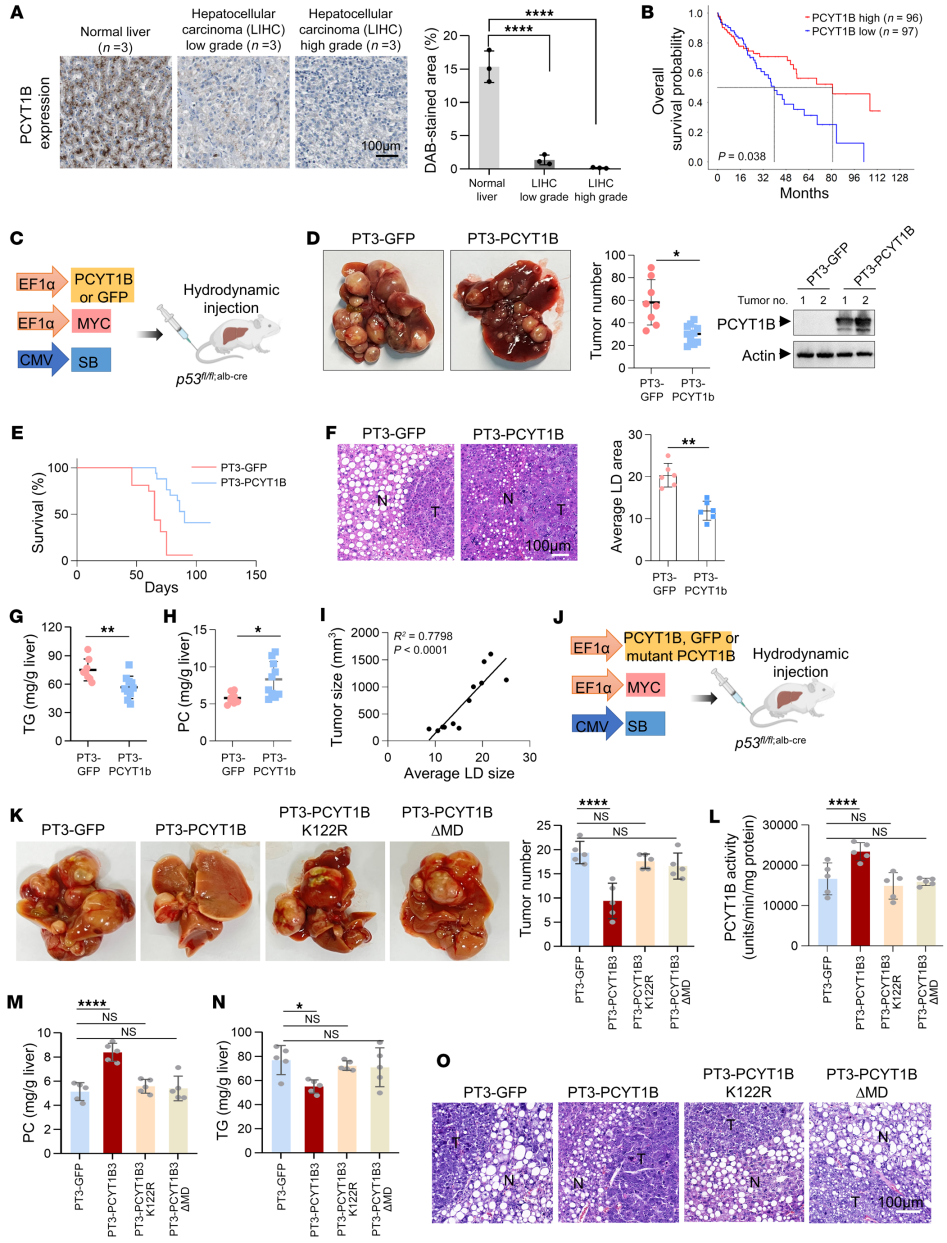
PCYT1B is a metabolic tumor suppressor. (**A**) Expression of PCYT1B in normal liver tissues and carcinoma of different stages was determined by immunohistochemistry (Human Protein Atlas; https://www.proteinatlas.org). Scale bar: 100 μm. (**B**) Kaplan-Meier survival curves of patients with liver cancer based on PCYT1B expression. Gray dotted lines indicate median survival time. (**C**–**I**) PCYT1B suppresses liver tumorigenesis in *p53^fl/fl;alb-cre^* mice. (**C**) Diagram depicting generation of liver tumor models. (**D**) Right: Representative expression of PCYT1B in tumors. Left: Representative images of liver tumor multiplicity and number of tumors. *n* = 8 (for PT3-GFP injection) or 10 (for PT3-PCYT1B injection) mice per group. (**E**) Kaplan-Meier survival curves of *p53*^fl/fl;alb-cre^ mouse hydrodynamic tail vein injection (HTVI) with transposons expressing MYC and PCYT1B (GFP). *n* = 16 for GFP group; *n* = 17 for PCYT1B group. (**F**) Representative histological analysis of the tumors stained for H&E. Images and quantification were taken at tumor periphery. Scale bar: 100 μm. Levels of liver TG (**G**) and PC (**H**) were measured. Linear regression analysis was performed on mean LD size and tumor size (**I**) (*n* = 12). (**J**–**O**) *p53^fl/fl;alb-cre^* mice were fed a choline-free diet 6 weeks after injection with the indicated components using HTVI (**J**). (**K**) Representative images and number of liver tumors. (**L**) Liver tumor tissue PCYT1 activity. Absolute levels of PC (**M**) and TG (**N**) in the livers were measured. (**O**) Representative histological analysis of the tumors stained for H&E. *n* = 5 per group. Scale bar: 100 μm. All data are mean ± SD. Each experiment was carried out at least 3 independent times. *P* values were calculated by 2-tailed unpaired Student’s *t* test (**A**, **D**, **F**, **G**, and **H**), 2-way ANOVA followed by Dunnett’s multiple-comparison test (**K**–**N**), linear regression analysis (**I**), or log-rank (Mantel-Cox) test (**B** and **E**). *P < 0.05, **P < 0.01, ****P < 0.0001.

By comparing the expression of PCYT1B in human hepatocellular carcinoma (HCC) of different grades and in normal tissues, we found that expression of this enzyme was significantly high in normal tissues but strongly declined in high-grade tumors, negatively correlating with tumor progression (Figure 5A). Moreover, decreased PCYT1B expression was significantly correlated with poor patient survival (Figure 5B), suggesting that PCYT1B may play a role in suppressing tumor development.

To explore the effect of PCYT1B on tumorigenesis, we established an HCC model (20), in which *p53^fl/fl;alb-cre^* mice fed a choline-deficient diet were coinjected with the MYC oncogene along with PT3 plasmid DNA expressing PCYT1B or GFP control (Figure 5C). PCYT1B-injected mice showed a reduction in tumor burden in the liver and increased survival rate (Figure 5, D and E). The mTORC signaling pathway is a major determinant of tumorigenesis and can be activated by sufficient fatty acids (21, 22). Consistently, a strong reduction in tumor proliferative activity and mTORC1 signaling activity was observed in PCYT1B-overexpressing mice (Supplemental Figure 10, A and B), with a decrease in LDs and TG content and a corresponding increase in PC abundance in the liver (Figure 5, F–H). Notably, by measuring the size of LDs and tumors in the livers of all treated mice, we found a significant positive correlation between LD size and tumor size (Figure 5I). The body and liver weights and plasma TG as well as blood glucose of mice injected with PCYT1B did not change significantly, except for a decrease in ALT levels (Supplemental Figure 10, C–E). Also, we used diethylnitrosamine (DEN) combined with carbon tetrachloride (CCl_4_) (DEN-CCl_4_) to establish another liver cancer model in *p53^fl/fl;alb-cre^* mice fed a choline-deficient diet to verify the tumor-suppressive ability of PCYT1B (Supplemental Figure 10, F and G). Similarly, in this system, liver-specific overexpression of PCYT1B (Ad-PCYT1B) did not alter body and liver weight but significantly reduced both the number and size of tumors (Supplemental Figure 10, H and I). Also, a strong decrease in proliferative effect was observed in PCYT1B-overexpressed tumors (Supplemental Figure 10, J and K). Moreover, PCYT1B-overexpressed liver tissues displayed increased PC levels and correspondingly reduced TG and lipid contents (Supplemental Figure 10, L–N). Again, we found that the size of LDs positively correlated with tumor size in these mice (Supplemental Figure 10O). In addition, PCYT1B overexpression did not change plasma TG and blood glucose and to some extent affected liver function (Supplemental Figure 10, N, P, and Q). Thus, these results suggest that PCYT1B acts as a tumor suppressor.

We next investigated whether the enzymatic activity of PCYT1B is required for its tumor-suppressive function. We injected *p53^fl/fl;alb-cre^* mice with constructs expressing MYC oncogene along with GFP control, WT PCYT1B, or enzymatic activity–dead mutant PCYT1B bearing K122R mutation or M domain depletion (ΔMD) (Supplemental Figure 11, A and B). Compared with mice injected with WT PCYT1B, neither PCYT1B-K122R– nor PCYT1B-ΔMD–expressing mice showed an inhibitory ability for tumorigenesis (Figure 5, J and K, and Supplemental Figure 11C), and indeed, no increase in the enzymatic activity of PCYT1 was observed in the tumors of these mice (Figure 5L). Consistent with this, K122R mutation or ΔMD totally blocked the ability of PCYT1B to modulate PC, TG, and lipid contents (Figure 5, L–O). As could be expected, mice expressing these PCYT1B mutants in the liver showed no change in body and liver weight or liver function (Supplemental Figure 11, D–F).

Collectively, these findings suggest that PCYT1B is a tumor suppressor whose enzymatic activity is indispensable for its tumor-suppressive capability.

### PC availability suffices for p53-mediated tumor suppression.

To determine the tumor-suppressive effect of PC, we injected *p53^fl/fl^* mice with constructs expressing the MYC oncogene and treated the mice with PC in the presence of a choline-deficient diet (Supplemental Figure 12A). PC administration led to increased levels of intrahepatic PC and did not affect the body and liver weight of the mice (Supplemental Figure 12, B and C). Notably, PC-treated mice exhibited suppressed tumor incidence and proliferative activity and decreased LDs and TG content in the liver (Supplemental Figure 12, D–G). Like PCYT1B overexpression, PC treatment improved liver function as indicated by a reduction in ALT activity (Supplemental Figure 12H). Consistent with this, lower PC levels in the liver of mice with liver-specific knockout of PCYT1B decreased tumor burden, TG levels, and mTORC signaling in the liver, while PC administration abolished these effects (Supplemental Figure 13, A–F).

**Figure 6:**
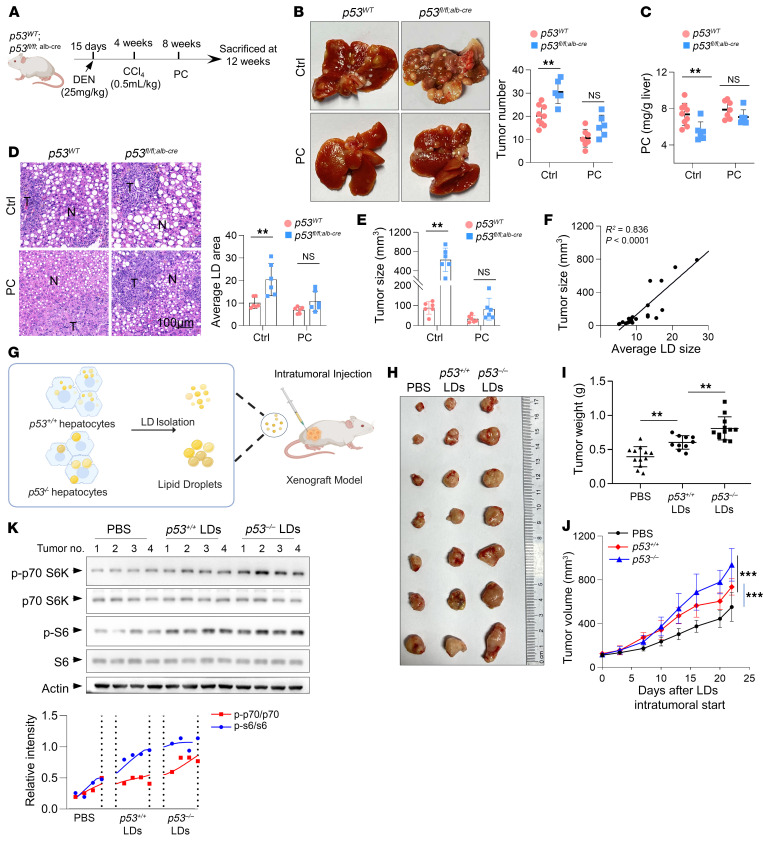
Downregulation of the Kennedy pathway is required for the development of liver tumors harboring p53 loss. (**A**) *p53^WT^* and *p53^fl/fl;alb-cre^* mice were treated with DEN, CCl_4_, and PC and fed a choline-free diet. (**B**) The number of liver tumors was measured at week 12 (right). Representative images of liver tumor multiplicity are shown (left). *n* = 9 control *p53^WT^* mice, 6 PC-treated *p53^WT^* mice, 7 control *p53^fl/fl;alb-cre^* mice, and 6 PC-treated *p53^fl/fl;alb-cre^* mice per group. (**C**) Absolute levels of PC in the livers were measured. (**D**) Left: Representative histological analysis of the tumors stained for H&E. Right: LD area was quantified. Images and quantification were taken at tumor periphery. N, normal tissue; T, tumor tissue. (**E**) Tumor size was assessed. (**F**) Linear regression analysis was performed on mean LD size and tumor size in the murine livers (*n* = 24). (**G**–**K**) Lipid droplets isolated and purified from the equal amount of liver tissues from *p53^WT^* and *p53^fl/fl;alb-cre^* mice fed a choline-free diet were injected into the transplanted tumor derived from HepG2 cells in NCG mice twice a week (**G**). (**H**) Representative images of transplanted tumors. (**I**) Average weights of xenograft tumors on day 22. *n* = 12 tumors for PBS group; *n* = 10 tumors for *p53^+/+^* LDs group; *n* = 12 tumors for *p53^–/–^* LDs group. (**J**) Tumor volume. (**K**) Tumors were analyzed by Western blot. All data are mean ± SD. Each experiment was carried out at least 3 independent times. *P* values were calculated by 2-tailed unpaired Student’s *t* test (**B**–**E**), 2-way ANOVA followed by Holm-Šidák multiple-comparison test (**I**), linear regression analysis (**F**), or 2-way ANOVA (**J**). **P < 0.01, ***P < 0.001.

We next investigated whether PC is necessary for p53-mediated tumor suppression. Compared with p53 WT mice, *p53^fl/fl;alb-cre^* mice developed liver tumors of larger size and at higher frequency in the setting of DEN-CCl_4_ treatment and choline-free diet (Figure 6, A–C and G). Strikingly, PC administration restricted tumorigenesis and minimized the difference between *p53^+/+^* and *p53^–/–^* mice (Figure 6, A–C and E). Moreover, increased tumor proliferative activity and cellular mTORC1 signaling, as well as LD and TG contents in the liver produced by p53 loss, were all reversed by PC treatment (Figure 6D and Supplemental Figure 13, G–I). In the system, LD size was also significantly correlated with tumor size (Figure 6, E and F). No obvious change in body or liver weight, or plasma TG or blood glucose, was observed, but, interestingly, PC treatment reversed the liver dysfunction of *p53^–/–^* mice (Supplemental Figure 13, J–L). In addition, *p53^–/–^* mice exhibited constitutive low expression of PCYT1B before and after the onset of tumors induced by DEN, suggesting that downregulation of PCYT1B is compatible with tumor initiation in the absence of p53 and may facilitate tumorigenesis from an early time (Supplemental Figure 14, A and B). Collectively, these data suggest that blockade of PC synthesis contributes to the development of p53-deficient liver tumors.

### LDs support tumor growth in vivo.

We then were curious to see whether LDs could also support tumor growth in vivo, especially those derived from p53-deficient mice fed a choline-free diet. To this end, LDs were isolated and purified from the equal amount of liver tissue from mice fed a choline-deficient diet and then injected into the transplanted tumor, a strategy that allows a crude comparison of the direct effect of LDs on tumor growth (Figure 6G). Strikingly, intratumoral injection of LDs promoted tumor growth, whereas those isolated from *p53^–/–^* liver had a more profound effect (Figure 6, H–J). Western blot analysis revealed that tumors with exogenous LD supplementation exhibited increased mTORC signaling activity, and consistently, even higher levels of mTORC signaling activity were observed in tumors receiving *p53^–/–^* LDs (Figure 6K). Thus, these results suggest that LDs can support tumor growth and that p53-deficient liver-derived LDs appear to have a stronger effect.

### Defects in the p53/PCYT1B/PC axis increase the surface localization of HSL on LDs to release specific fatty acids.

We also wanted to know why or how LD growth induced by p53 inactivation accompanies tumorigenesis during choline starvation. Prevention of endoplasmic reticulum (ER) stress by LDs is thought to help tumor cells survive (23). However, p53 knockout had minimal effect on the activation of the ER stress response, as measured by the expression of XBP1s/XBP1u and the ER chaperone Bip in choline-starved HepG2 cells, even during oleic acid stimulation (Supplemental Figure 14, C and D). Similarly, no differences in Bip mRNA levels in the liver were observed between *p53^+/+^* and *p53^–/–^* mice fed a choline-free diet (Supplemental Figure 14E), suggesting that improvement of ER homeostasis is not the underlying mechanism.

**Figure 7:**
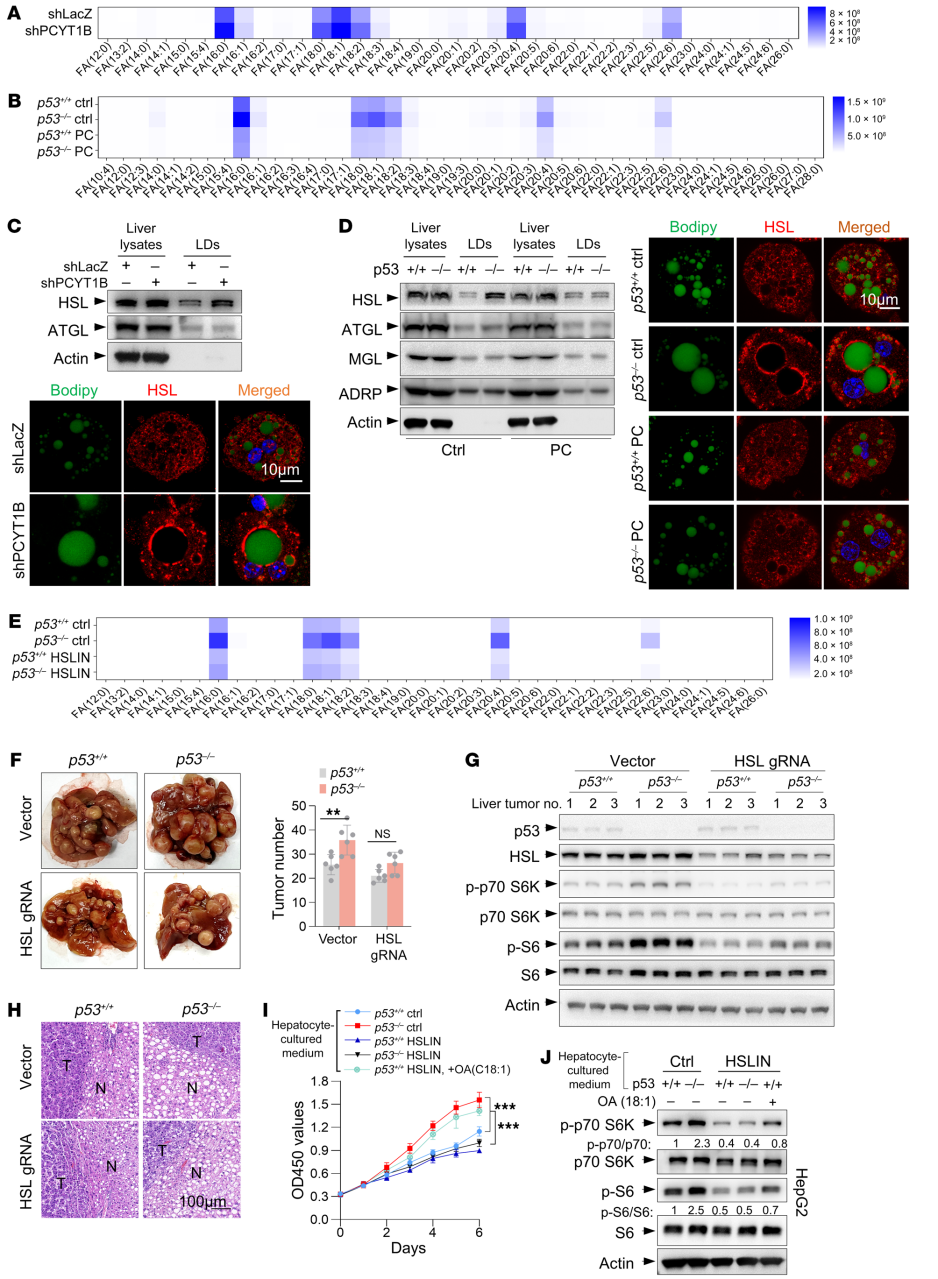
Suppression of PC biosynthesis by p53 loss increases surface localization of HSL to release specific fatty acids as fuel for tumorigenesis. (**A** and **C**) Mice treated with shLacZ or shPCYT1B adenoviruses were maintained on a choline-deficient diet. (**A**) Fatty acid levels in the interstitial fluid of liver tissue. (**C**) Liver tissues and purified liver LDs were analyzed by Western blot, and isolated hepatocytes were immunostained as indicated. Scale bar: 10 μm. (**B** and **D**) *p53^+/+^* and *p53^–/–^* mice were maintained on a choline-free diet with or without oral administration of 300 μL PC (10 mg/mL) daily. (**B**) Fatty acid levels in the interstitial fluid of liver tissue. (**D**) Liver tissue and purified liver LDs were analyzed by Western blot, and isolated hepatocytes were immunostained as indicated. Scale bar: 10 μm. (**E**) Levels of fatty acids in liver tissue interstitial fluid from *p53^+/+^* and *p53^–/–^* mice maintained on a choline-free diet with or without oral administration of 100 μL HSL inhibitor (HSL-IN-1, 5 mg/mL) daily. (**F**–**H**) The experimental procedures are illustrated in Supplemental Figure 15E. (**F**) Representative images of liver tumor multiplicity and the number of tumors. (**G**) Liver tissues were analyzed by Western blot. (**H**) Representative histological analysis of H&E-stained tumors. *n* = 6 mice per group. Scale bar: 100 μm. (**I** and **J**) *p53^+/+^* and *p53^–/–^* mice were maintained on a choline-free diet with or without oral administration of 100 μL HSL inhibitor (5 mg/mL) daily. The isolated hepatocytes were cultured in lipid-free medium, and the conditioned medium containing no oleate or 50 μM oleate was then used to culture HepG2 cells for different times. HepG2 cell proliferation and mTORC1 activity were determined. OA, oleic acid. Data are mean ± SD. Each experiment was carried out at least 3 independent times. *P* values were calculated by 2-tailed unpaired Student’s *t* test (**F**) or 2-way ANOVA (**I**). **P < 0.01, ***P < 0.001.

Interestingly, metabolomic analysis revealed a strong accumulation of 6 specific free fatty acids (FFAs) 16:0, 18:0, 18:1, 18:2, 20:4, and 22:6 in the liver interstitial fluid of PCTY1B-knockdown mice (Figure 7A). Strikingly, the loss of p53 consistently led to the accumulation of these 6 specific FFAs in the liver interstitial fluid, and PC administration completely abolished this phenomenon (Figure 7B and Supplemental Figure 14F). Therefore, it is likely that LD growth induced by defects in the p53/PCYT1B/PC axis fuels tumor cells by providing specific FFAs. p53 did not affect the expression of genes involved in FFA uptake in tumor cells (Supplemental Figure 14, G and H). Next, we were curious about how LD growth alters FFA levels in the microenvironment. The increase in surface area of LD and decrease in PC synthesis induced by p53 loss or inhibition of the Kennedy pathway may increase the surface localization of certain lipases on LDs to generate fatty acids, which are released as fuel for tumors. To test this possibility, we fractioned mouse liver tissues and purified LDs. Interestingly, higher levels of HSL were found in the LD fraction of shPCYT1B-silenced livers, and confocal imaging analysis further revealed more HSL on the LD surface when PCYT1B was knocked down (Figure 7C). ATGL and HSL are the major lipases that are functionally localized to LDs (24). By contrast, LD localization of ATGL was not altered by PCYT1B ablation (Figure 7C). Consistently, p53 knockout resulted in a strong increase in HSL but not in other lipases on LDs in mouse liver and hepatocytes (Figure 7D). When PC was sufficient, this effect disappeared (Figure 7D). In line with the effect of PCYT1B depletion or p53 knockout on FFAs, inhibition of HSL reduced the levels of the 6 FFAs in liver interstitial fluid and minimized the difference between *p53^+/+^* and *p53^–/–^* mice (Figure 7E). Likewise, depletion of PCYT1B or p53 in hepatocytes increased extracellular FFA levels, whereas HSL inhibition or PC supplementation reduced FFA secretion (Supplemental Figure 14I). Thus, p53 loss or defects in the Kennedy pathway increase surface localization of HSL on LDs for release of specific FFAs. These findings were further strengthened by the observations that loss of p53 did not alter either protein or mRNA expression of HSL (Figure 7, C and D, and Supplemental Figure 14J).

### Fatty acids released by HSL fuel tumorigenesis in p53-deficient mice.

We further investigated the effect of HSL on tumorigenesis using an HCC model (20) by injecting mice with the MYC oncogene along with CRISPR plasmid DNA expressing Cas9 and sgRNAs targeting Axin1 (Supplemental Figure 15A). In agreement with the fatty acid data (Figure 7E), HSL inhibition blunted the tumor burden and proliferation of tumor cells in the liver of *p53^–/–^* mice (Supplemental Figure 15, B and C). Notably, *p53^–/–^* tumors exhibited higher mTORC activity compared with *p53^+/+^* tumors, and HSL inhibition strongly attenuated this phenomenon in both tumor types (Supplemental Figure 15D). Consistently, liver-specific knockdown of HSL reduced the number and proliferative activity of tumors in the liver of *p53^–/–^* mice (Figure 7F and Supplemental Figure 15, E and F) and strongly impeded mTORC activity in both *p53^+/+^* and *p53^–/–^* tumors (Figure 7G). In contrast, injection of HSL sgRNA had no effect on LD levels (Figure 7H), supporting the notion that HSL acts downstream of LD growth to influence tumor development.

We also extended these findings to in vitro cell culture systems. Culturing with PCYT1B- or p53-deficient liver interstitial fluid significantly promoted HepG2 cell proliferation and mTORC activation in these tumor cells (Supplemental Figure 16, A–F). Similar findings were obtained using PCYT1B- or p53-deficient hepatocyte-conditioned medium to culture tumor HepG2 cells (Figure 7, I and J, and Supplemental Figure 16, G–K). CD36 is a major player in the fatty acid uptake in metabolic tissues (25). Blocking fatty acid uptake by addition of CD36 inhibitor completely abolished HepG2 cell proliferation induced by culture with PCYT1B-silenced or p53-deficient liver interstitial fluid or hepatocyte-conditioned medium (Supplemental Figure 16, A, H, and I), suggesting the importance of microenvironmental fatty acids in fueling tumor cell proliferation. Consistent with the above-mentioned findings, culture with liver mesenchyme from mice treated with PC or HSL inhibitor, or with conditioned medium from hepatocytes treated with PC or HSL inhibitor, resulted in suppressed proliferation of HepG2 cells and reduced mTORC activity of tumor cells (Figure 7, I and J, and Supplemental Figure 16, B, C, E, F, J, and K). Notably, proliferation and mTORC activity of cells cultured in conditioned media of HSL-inhibited hepatocytes were largely restored upon further addition of oleic acid (18:1) (Figure 7, I and J), reinforcing the notion that fatty acids support tumor cell proliferation induced by p53 deletion or the Kennedy pathway defects.

## Discussion

Here, we identify a role for p53 in LD biology and metabolic fueling of tumors by LD growth through Kennedy pathway regulation (Supplemental Figure 17A). Coupled with the finding that choline starvation promotes LD growth and liver lipid accumulation in the absence of p53, we observed increased LD accumulation associated with tumorigenesis in p53-deficient mice, and PC restoration was able to inhibit tumor burden. In order to functionally explore the contribution of the Kennedy pathway in the early stages of p53-mediated tumor suppression, we also looked at changes in PCYT1B expression after DEN treatment but before the onset of frank tumors or after tumor onset in choline-free diet mice. *p53^–/–^* mice showed persistently low expression of PCYT1B before and after tumor onset, compared with *p53^+/+^* mice. Moreover, low expression of PCYT1B was accompanied by a corresponding increase in LD accumulation, suggesting that inhibition of PCYT1B is compatible with tumor initiation in the absence of p53 and may promote the accumulation of LDs in normal hepatocytes that facilitate tumorigenesis from an early time.

Under certain conditions of nutrient stress, the hydrolysis of LDs is thought to be associated with cancer cell survival and may function as a storage of cellular excess of lipid molecules to counteract stress. However, cancer cells usually contain large amounts of LDs, and how this happens is still a mystery. Some studies suggest that LDs may protect tumor cells either by improving ER homeostasis or by acting as scavengers of reactive oxygen species in tumor cells (26). Unexpectedly, here we found that during choline starvation, LD growth induced by p53 loss–mediated suppression of the Kennedy pathway can actually increase the surface localization of HSL on LDs to release fatty acids as fuel for tumorigenesis. The exact role of LD in tumorigenesis needs further investigation.

Since HSL, but not ATGL, alters the levels of some specific fatty acids, it is possible that these fatty acids are derived from cholesteryl esters. Direct liquid chromatography–mass spectrometry (LC-MS) analysis of liver tissue from mice on a choline-deficient diet showed that certain cholesteryl esters were reduced in p53-deficient or PCYT1B-depleted liver tissues. Furthermore, these cholesteryl esters were mostly enriched in the same 6 fatty acyl chains found in the specific fatty acids, and PC treatment restored the levels of these cholesteryl esters (Supplemental Figure 17, B and C). It is therefore likely that these specific fatty acids are produced by the hydrolysis of cholesteryl esters.

HCC is a particularly challenging tumor with no effective therapies or druggable drivers. Consistent with our observations, human liver tumors with reduced expression of PCYT1B, like those with p53 loss, are associated with poor prognosis and patient survival, suggesting the therapeutic potential of targeting the Kennedy pathway for potential prevention of p53-deficient liver tumors. Together with this, our findings reveal a role for p53 in the regulation of LD growth, provide insights into the understanding of the physiological functions of p53, and have theoretical implications for the clinical treatment of p53-mutant tumors as well as other human diseases associated with excessive lipid storage mediated by LDs.

## Methods

### Semiquantitative and quantitative reverse transcriptase PCR.

Briefly, total RNA was isolated from triplicate wells in each condition using the Total RNA Purification Kit (GeneMark, TR01), and 2 μg RNA of each sample was reversed to cDNA by First-Strand cDNA Synthesis System (Thermo Fisher Scientific, K1621). Next, 0.04 μg cDNA product of each sample was used as template to conduct semiquantitative or quantitative PCR. Quantitative PCR was performed on a CFX96 Real-Time PCR System (Bio-Rad), and the amplifications were done using the SYBR Green PCR Master Mix (GeneStar, A112-100). The fold changes of gene expression were calculated after being normalized to ACTB. The primers used in this study are listed in Supplemental Table 1.

### ^13^C-labeled choline tracer studies.

*p53^+/+^* and *p53^–/–^* HepG2 cells were cultured in choline-free medium for 12 hours. The medium was then removed, and choline-free medium supplemented with 200 μM [1,2-^13^C_2_]choline (Cambridge Isotopes, CLM-548-0.1) was substituted for a further 30 minutes. The cells were washed twice with PBS, and then related metabolites were extracted and analyzed by LC-MS (see Supplemental Methods for details).

### Sex as a biological variable.

Our study exclusively examined male mice. It is unknown whether the findings are relevant to female mice.

### Statistics.

No statistical methods were used to predetermine sample size. All the results are shown as means ± SD. All statistical methods used are specified in the figure legends. All statistical analysis was performed and *P* values were obtained using GraphPad Prism software 7.0 (GraphPad Software Inc.). An unpaired 2-tailed Student’s *t* test (2-way ANOVA) was used to calculate the *P* values, unless specified otherwise. *P* less than 0.05 was considered significant.

### Study approval.

All mice were maintained under specific pathogen–free conditions and used in accordance with protocols approved by the Institutional Animal Care and Use Committee (IACUC) of Tsinghua University for animal welfare. The laboratory animal facility was licensed by the IACUC.

### Data availability.

All data supporting the findings of this study are included in this article or in the Supporting Data Values file. No new code or algorithms were generated. The RNA-Seq data reported in this study were deposited in the NCBI’s BioProject database (BioSamples SAMN37571050, SAMN37571051, SAMN37571052, SAMN37571053, SAMN37571054, and SAMN37571055). Materials generated by the authors and used in this study are listed in Supplemental Methods and are available from the corresponding authors on reasonable request.

## Author contributions

XX, PL, and PJ designed the experiments. XX performed all of the experiments and collected and analyzed the data. JW and LX provided technical assistance. PL and PJ supervised the research, and PJ interpreted the data and wrote the manuscript. All authors commented on the manuscript.

## Supplementary Material

Supplemental data

Unedited blot and gel images

Supplemental video 1

Supplemental video 2

Supporting data values

## Version 1 01/09/2024

In-Press Preview

## Version 2 02/15/2024

Electronic publication
