# Supplementary material for: p53 suppresses lipid droplet–fueled tumorigenesis through phosphatidylcholine
Source: J Clin Invest. 2024 Jan 9;134(4):e171788. doi: 10.1172/JCI171788 (PMC10866454; doi:10.1172/JCI171788)
Supplement: Supplemental data [file jci-134-171788-s026.pdf]

Supplemental Information

p53 suppresses lipid droplet-fueled tumorigenesis through phosphatidylcholine

Xiuduan Xu, Jianqin Wang, Li Xu, Peng Li, and Peng Jiang

Supplemental Materials and Methods

Supplemental Figures 1-17

Supplemental Tables 1

Supplemental Materials and Methods

Antibodies and reagents. The antibodies against the following proteins were purchased from the indicated companies: anti-p53 (Santa Cruz, Cat#sc126-HRP, 1: 1,000 for IB), anti-p53 (CST, Cat#2524T, 1: 1000 for IB), anti-p21 (BD, Cat#556431, 1: 1,000 for IB), anti-Actin (Proteintech, Cat#66009-1-LG), anti-PCYT1B (Proteintech, Cat#13765-1-AP, 1: 150 for IF), anti-PCYT1B (Abcam, Cat#ab127200, 1: 1,000 for IB), anti-PCYT1B (Novus Biologicals, Cat#NBP1-86563, 1: 100 for IHC), anti-Ki67 (Abcam, Cat#ab15580, 1: 600 for IHC), anti-BSCL2/Seipin (Abcam, Cat#ab106793, 1: 1000 for IB), anti-Monoglyceride Lipase (Santa Cruz, Cat#sc-398942, 1: 1,000 for IB), anti-CIDEB (Invitrogen, Cat#PA5-101329, 1: 1000 for IB), anti-ATGL (CST, Cat#2138, 1: 1000 for IB), anti-HSL (CST, Cat#4107, 1: 1000 for IB) anti-Perilipin-1 (CST, Cat#9349, 1: 1000 for IB).

The following reagents were purchased from indicated companies: Nutlin-3 (Sigma, SML0580), DOX (Sigma, D1515), Etoposide (Sigma, E1383), BODIPY 493/503 (Invitrogen, D3922), OCT (SAKURA, 4583), Protein A/G agarose (Pierce, 20421), PD-10 desalting column (GE Healthcare, 17085101), Centrifugal filter (Millipore, 100k-UFC910008-8), Alexa Fluor 647 fluorescent azide (Life Technologies, A10277), Hoechst 33,342 (Enzo Life Sciences, 23,491-52-3), Choline Chloride (Sigma, C7527), CHOLINE CHLORIDE (1,2-13C2, 99%) (Cambridge Isotope Laboratories, CLM-548-PK), William's E medium (Caisson Labs, WMP03-10X1LT), L-α- Phosphatidylcholine (Sigma, P3782), citicoline (MCE, HY-B0739), Propargyl-choline (AOBIOUS, AOBT7378), oleate (Sigma, O7501), Tyloxapol (MCE, HY-B1068), BDP TMR azide (Lumiprobe, Cat#12430), Miltefosine (Avanti, 850337P), Egg phosphatidylcholine (Avanti, 840051P), DEN (Sigma, N0258-1G), CCl4 (Macklin, C805329), Sulfosuccinimidyl oleate sodium (MCE, HY-112847A).

Plasmids pT3-EF1a-c-myc-cre-GFP and CMV-SB10 were kindly gifted by Lei Zhang lab and were obtained from Bio-Research Innovation Center SUZHOU. Then we constructed pT3-EF1a-MYC-cre, pT3-EF1a-Pcyt1b and pT3-EF1a-MYC plasmids.

Cell culture, siRNA transfections and CRISP/Cas9-mediated deletion of p53. HEK293T cells, HEK293A cells, KEK293FT cells, A549 cells, MDA-MB-231 cells, DU145 cells, human colon cancer HCT116 cells, human osteosarcoma U2OS cells, human hepatocellular carcinoma Huh-7 cells and HepG2 were purchased from American Type Culture Collection (ATCC). *p53*$^{+/+}$ and *p53*$^{-/-}$ HCT116 cells were kindly gifted by Dr. Bert Vogelstein at John Hopkins

University. HEK293T, HEK293A cells, KEK293FT cells, DU145, A549, MDA-MB-231 cells, HCT116 and Huh-7 were maintained in standard culture medium. HepG2 cells were cultured in MEM containing 10 μM choline with 10% FBS or also were cultured in choline free William's E medium supplemented 100 μM methionine with 10% FBS, and U2OS was cultured in Maccoy's 5A with 10% FBS. All cells used in this study were cultured without penicillin-streptomycin solution. All cell lines were subjected to examination of mycoplasma contamination and were cultured for no more than 2 months. siRNA transfections were performed with Lipofectamine RNAiMAX (Life technology), using siRNA pools targeting human p53 and PCYT1B. These siRNAs were used at a concentration of 20 nM.

To generate *p53*-knockout HepG2, a lentiviral CRISPR/Cas9 plasmid targeting *p53* was created by cloning the annealed sgRNA into pLenti-CRISPRv2 vector. The sgRNAs were designed by CRISPR Design tool (crispr.mit.cn), and the sequences were: 5'-CACCGTCCATTGCTTGGGACGGCAA-3' and 5'-AAACTTGCCGTCCCAAGCAATGGAC-3'. 293T cells were co-transfected with pLenti-CRISPRv2, VSVg and psPAX2 to make lentiviruses. After infection, cells were selected by 2 μg/mL puromycin for a week.

Animals and Diets. Animal experiments were performed with male C57BL/6J mice (Jackson Laboratory, Jax664), *p53*$^{-/-}$ C57BL/6J mice (BIOCYTOGEN, BCG-DIS-0001), and *p53*$^{fl/fl}$ mice (Jackson Laboratory, Jax008462). The *p53*$^{fl/fl}$ mice was a kind gift of Dr. Wenjing Du, Institute of Basic Medical Sciences Chinese Academy of Medical Sciences, Beijing. Mice were initially randomized by age and weight, and were injected at 8-10 weeks of age.

Choline deficient L-Amino Acid Diet with 10% kcal fat and 0.1% methionine was purchased from SYSE Biotechnology (Changzhou, China, A06071310). The laboratory mice were maintained on a standard chow diet before the initiation of all dietary studies. The durations of each dietary challenge were noted in corresponding figure legends.

Western blot analysis. Cells were lysed by using a modified RIPA buffer containing 10 mM Tris-HCl at pH 7.5, 5 mM EDTA, 150 mM NaCl, 1% NP-40, 1% sodium deoxycholate, 0.025% SDS and proteinase inhibitors on ice for 20 minutes. Protein samples were quantified using BCA protein assay kit (Macgene, China), boiled in 5 x loading buffer for 10 minutes and equal amounts of proteins were resolved by SDS-PAGE, and transferred onto nitrocellulose membrane. 5% skimmed milk was used to block the membrane before immunoblotting with indicated antibodies overnight. Membranes were washed with TBST and then incubated with HRP-conjugated anti-rabbit or anti-mouse secondary antibodies at room temperature for one hour and developed with ECL Western Blotting Detection Reagent (Thermo Scientific, 32132). Blot bands were quantified using ImageJ software.

Chromatin Immunoprecipitation. To search for potential p53 response elements in PCYT1B gene, we used the Genomatix Promoter Inspector software (http://www.genomatix.de). In general, the consensus sequence for p53 response element is: 5'-RRRCWWGYYY-(0-13-base pair spacer)-RRRCWWGYYY-3', in which R is a purine, Y a pyrimidine, and W either A or T. The putative p53 response elements for PCYT1B and the sequences are: PCYT1B-RE1, 5'-CAGCTGCATGTCTGGACTCAAACCC-3'; PCYT1B-RE2, 5'-AGAGACCAGGTTTTGCCATGTTACC-3'; PCYT1B-RE3, 5'-AAGCAGCAAGTTCGGGCCCGTGGGT-3', Chromatin immunoprecipitation assays were performed as previously. Briefly, cells were treated with or without 0.5 μg/mL dox for 12 hours

and crosslinked with 1% formaldehyde solution for 15 minutes at room temperature (25°C). The crosslinking reaction was stopped by the addition of glycine to 125 mM final concentration and lysed in 1 mL SDS lysis buffer for 10 minutes on ice. Lysates were then sonicated to generate DNA fragments with the average size approximately 200-1000bp. Sonicated samples were spun down and subjected to overnight immunoprecipitation with p53 antibodies or control IgG. The next day, bounded DNA fragments were eluted using low salt immune complex buffer, high salt immune complex buffer, and TE buffer sequentially, and amplified by PCR. The primer pairs were: PCYT1B-RE1-F, 5'- CGCGGATCCACCTATGGTCTTACTCATCCAC-3', PCYT1B-RE1-R, 5'- ACGCGTCGACCACACATGTAGTGCAGTTTAATC-3'; PCYT1B-RE2-F, 5'- CGCGGATCCCTTACTCTGTCATCTAGGTTGGAGTGC-3'; PCYT1B-RE2-R, 5'- ACGCGTCGACTAGCACTTTGGGAGGCCGAGGCAGGTG-3'; PCYT1B-RE3-F, 5'- CGCGGATCCCTGCTATGTTGTTTCTCAAGGCGTC-3', PCYT1B-RE3-R, 5'- ACGCGTCGACCATTTGCATAGAGCGCGCTGGTTGG-3'.

Luciferase activity assay. Briefly, the genomic fragment of PCYT1B containing either the wild-type or mutant p53-binding region was cloned into pGL3-basic vector. The reporter plasmids were then transfected into HEK293T cells together with a Renilla luciferase plasmid, and Flag-p53 plasmid or vector control plasmid. 24 hours after transfection, the luciferase activity was determined using a dual Luciferase Assay System (Promega, E1910). Transfection efficiency was normalized based on the Renilla luciferase activity.

Soft agar assay. For soft agar assay, HepG2 cells were suspended in 1 mL of choline-deficient medium supplemented with or without 100 μM choline or 10 μg/mL L-a-PC plus 20% FBS containing a 0.3% agarose and plated on a firm 0.6% agarose base in 12-well plates. Cells were then cultured in 5% CO_2 incubator at 37°C for 2 weeks. Colonies were fixed with 25% formaldehyde and stained with 0.0125% crystal violet till colonies turned into blue. Colonies were then quantified by counting and images were obtained.

Propargyl-Choline labeling of cells tracer studies. Propargyl-choline bromide was synthesized from propargyl-bromide and dimethyl-ethanolamine. $p53^{+/+}$ and $p53^{-/-}$ HepG2 cells were grown in 35 mm glass bottom dishes and labeled with 10 μM and 100 μM Propargyl-Choline in choline free medium for 12 hours. The cells were washed twice with PBS and fixed for 20 minutes with 4% formaldehyde. Then the plate was washed with TBS (50 mM Tris-Cl, 150 mM NaCl, pH 7.5) and reacted with 20 μM Alexa Fluor 647 fluorescent azide (Life Technologies, A10277) for 30 minutes in 100 mM Tris pH 8.5 buffer contained 0.75 mM $CuSO_4$ and 75 mM ascorbic acid. The cells were washed with TBS, 0.5 M NaCl, and then with TBS again before counterstaining with Hoechst 33,342 (Enzo Life Sciences, 23,491-52-3). Cell images were acquired on confocal laser scanning microscope.

PCYT1 enzymatic activity assay. Briefly, liver tissues were rapidly homogenized in ice-cold lysis buffer with a loose-fitting Dounce. The liver homogenates were centrifuged at 13000g for 15min. The reaction mixture contained 30 mM Tris, pH 7.4, 12 mM $MgCl_2$, 10 mM CTP, 10 mM DTT, 250 μM EDTA, 88 mM NaCl, and 10 μg of liver tissue homogenates, and 1.6 mM phosphocholine, in a final volume of 50 μL. After 30 min in a shaking water bath, the reaction was stopped by adding 30 μL of methanol/ammonia (9:1) solution. The amount of CDP-choline formed was determined by LC-MS.

Propargyl-Choline labeling of liver tissue tracer studies. *p53* wild-type (WT) and *p53*$^{-/-}$ mice

maintained on a choline-free diet were tail-vein injected with 0.05mg/g propargyl-choline for 24 hours. Liver tissues were removed 24 hours later and fixed in 4% formalin for 24 hours. Then the liver tissues were dehydrated in 10%, 15%, 30% and 40% sucrose and embedded in OCT, sectioned 10 μm on a cryostat. The following was as described of the above Propargyl-Choline labeling of cells tracer studies.

BODIPY 493/503 Staining of Neutral Lipid Droplets. Cells were cultured with 200μM oleate or fatty acid free BSA for 12 hours. The dye 4,4-difluoro-1,3,5,7,8-pentamethyl-4-bora-3a,4a-diaza-s-indacene (Thermo Fisher, BODIPY 493/503) was prepared at 30 mM concentration in PBS. 2 μM BODIPY solutions was staining for 10 minutes, protected from light, then staining solutions were removed. The cells were washed in PBS twice before imaging on Olympus FV3000 microscope. For living cell imaging, cells were always cultured in choline-free medium containing 2 μM BODIPY and 200 μM oleate. Quantification of LD number, size and coalescence movie was carried out using ImageJ software.

Adeno-Associated Virus Construction and Preparation. The mouse *Pcyt1b* gene were cloned into the pAAV2/8 system, a gift from James M. Wilson (Addgene plasmid #112864 and #112867). 10 X 15cm dishes cells were cultured for 20~24 hours before transfection. To prepare transfection mix, aliquot 50 mL DMEM (FBS free) contained 70 μg AAV-TBG-Pcyt1b or AAV-TBG-GFP, 200 μg Delta F6 helper plasmid and 70 μg AAV2/8, then add 1020 μL PEI (the ratio of DNA: PEI is 1: 3) and mix immediately. Then incubated for 15 minutes. Mix and split the mixture equally into each dish. The medium was changed after transfection for 16 hours with complete media. Then the virus was harvested 60 hours after transfection. The adeno-associated virus (AAV) was packaged in HEK293FT cells, concentrated and purified the virus via discontinuous iodixanol gradient. The quality of AAV preparation was monitored by Coomassie Brilliant Blue (CBB) G-250 staining and quantified viral titer by real-time qPCR using SYBR Green assays. Then 10^11 AAV particles were tail-vein injected into $p53^{-/-}$ mice. Virus-infected mice were sacrificed 14 days after AAV injection.

Adenovirus Construction and Preparation. Adenoviruses expressing shRNA targeting mouse PCYT1B (shPCYT1B) were cloned into pENTER/U6 and recombined into the BLOCK-iT Adenoviral RNAi Expression System. The shRNA sequence was designed by Invitrogen BLOCK-iT™ RNAi Designer (http://rnaidesigner.thermofisher.com/rnaiexpress/). The LacZ shRNA was cloned into the pAD/Block-iT-DEST system as a negative control. The primers used for shRNA-expressing plasmid construct were shown in **Supplemental Table 1**. The adenoviruses were packaged and propagated in HEK293A cells in 6-well plate firstly, enlarged in 10cm dish, freeze and thaw three times, 4°C 3000rpm centrifugation for 20 minutes. Then the supernatant was purified by 55% cesium chloride gradient centrifugation at 25,000rpm for 22 hours, and desalted by PD-10 desalting column (GE Healthcare, 17085101). The viral titer was quantified by Nanodrop. Mice were tail-vein injected with 0.1 OD adenoviruses. The mice were sacrificed 10 days after virus injection, then liver tissue was used for biochemical and phenotypic characterizations.

Hepatocarcinoma mouse Models. For hydrodynamic tail vein injection-induced hepatocarcinoma model, pT3-EF1a-MYC-cre (20 μg), pT3-EF1a-Pcyt1b (20 μg), pT3-EF1a-Pcyt1b mutant (20μg) and CMV-SB10 Transposase (1:5 ratio) plasmids and a CRISPR plasmid targeting either HSL or AXIN1 were mix in sterile 0.9% NaCl solution for each injection. Male $p53^{fl/fl}$ and $p53^{fl/fl;\ alb\text{-}cre}$ mice (6-8 weeks of age) were randomly assigned to experimental groups.

The 0.9% NaCl solution/plasmid was injected into the lateral tail vein with a total volume corresponding to 10% of body weight in 5-7s. Mice were further analyzed after 8 weeks of injection. In pharmacological studies, mice induced tumor were treated orally with dimethyl sulfoxide (DMSO) or HSL inhibitor (5 mg/kg once daily) for 4 weeks. For DEN/CCl_4-induced hepatocarcinoma model, Two-week-old mice were administered with one single i.p. injection of diethyl nitrosamine (DEN, Sigma, 25 mg/kg in PBS). After 2 weeks, CCl_4 (CCl_4 in olive oil, 5mL mixture per kg body weight) were treated twice a week for 9 additional weeks, for the tumorigenesis experiments.

VLDL Secretion Assay in Mice. *p53*$^{+/+}$ and *p53*$^{-/-}$ mice were feed with choline-deficient diets for 7 days. Then the mice were fasted for 8 hours and injected with tyloxapol (Triton wr1339, MCE, HY-B1068) at the dose of 500 mg/kg body weight. Blood samples were collected at different time points and then separated by 4℃ 12000rpm centrifugation. Triglyceride level was measured by Triglyceride Quantification Colorimetric/Fluorometric Kit (Biovision, Cat# K622).

LC-MS analysis of Metabolites. Cells were seeded in 10cm dishes and cultured overnight. The following day, cells were washed twice in PBS and cultured in choline-deficient medium for 48 hours before subjecting to hydrophilic metabolites extraction. Samples analysis was performed using Q Exactive HFX orbitrap (Thermo, CA). 1 μL supernatant was loaded to normal phase chromatography column, then, the sample was eluted to orbitrap mass spectrometer with 50% ACN containing 10 mM ammonium formate as eluent. Data with mass ranges of m/z 70-500 was acquired at positive ion mode with data dependent MSMS acquisition. The full scan and fragment spectra were collected with resolution of 70,000 and 17,500 respectively. The source parameters are as follows: spray voltage: 3000v; capillary temperature: 320°C; heater temperature: 300°C; sheath gas flow rate: 35 Arb; auxiliary gas flow rate: 10 Arb. Metabolite identification was based on Tracefinder search with home-built database.

For phosphatidylcholine analysis, air-dried pellets were re-suspended in 100 μL of dichloromethane (CH_2Cl_2)/ Methanol (MeOH) (v: v = 1: 1), removed at the bottom by centrifugation at 12000 g for 20 minutes. An aliquot of 1 μL from the upper phase was withdrawn for mass spectrometry analysis. The UPLC system was coupled to a Q-Exactive HFX orbitrap mass spectrometer (Thermo Fisher, CA) equipped with a heated electrospray ionization (HESI) probe. Lipid extracts were separated by a CORTECS C18 100 × 2.1 mm 1.9 μm column (Waters). A binary solvent system was used, in which mobile phase A consisted of ACN: H_2O (60:40), 10 mM Ammonium acetate, and mobile phase B of IPA: ACN (90:10). A 35-minute gradient with flow rate of 250μL/min was used. Column chamber and sample tray were held at 45°C and 10°C, respectively. Data with mass ranges of m/z 240-2000 and m/z 200-2000 was acquired at positive ion mode and negative ion mode with data dependent MSMS acquisition. Data analysis and lipid identification were performed by the software lipid search 4.0 and TaceFinder 5.0 (Thermo Fisher, CA).

Electron Microscopy for Lipid Droplets Imaging. *p53*$^{+/+}$ and *p53*$^{-/-}$ HepG2 cells were cultured in choline-deficient medium incubated with 200 μM oleate by adding 50 μg/ml PC liposomes or no for 12 hours. HepG2 Cells were treated in a fixative 0.1M sodium cacodylate buffer (pH 7.4) containing 2.5% glutaraldehyde, 2.4% formaldehyde, and 1 mM calcium chloride in for 2 hours at room temperature. And then the cells were post fixed in a mixture of 1% osmium tetroxide, 0.1% potassium ferrocyanide, and 1 mM calcium chloride. Potassium

ferrocyanide could enhance contrast of the membrane. The sample was then dehydrated in 50%, 70%, 90%, 95%, and 100% ethanol for 5 minutes respectively, stained in 1% uranyl acetate in 100% ethanol, substituted with propylene oxide, and infiltrated with the Quetol812 resin mixture. After polymerization in an oven at 60°C for 2 days, Sections (70 nm) produced on a Leica EM UC6 ultramicrotome are prepared and counterstained by Reynold's lead citrate (Reynolds, 1963) before observation of lipid droplets by transmission electronic microscopy (TEM). The TEM was performed on a FEI Tecnai G2 Spirit BioTWIN TEM at 80 kV. Images were captured using an AMT camera.

Focused Ion Beam Scanning Electron Microscopy (FIB-SEM). Fixed $p53^{+/+}$ and $p53^{-/-}$ HepG2 cells were stained and embedded in epoxy resin as described of the above transmission electronic microscopy sample. A layer of gold was deposited on the cells. Then a layer of platinum (~600 nm thick) was deposited on a surface perpendicular to the block face. The block surface FIB/SEM image was obtained using an electron beam with 2 keV acceleration voltage, 0.4 nA beam current, and 10 μs/pixel dwell time. Then the new surface for serial FIB/SEM imaging was generated by 20-nm-thick superficial layer from the block face for the next round of imaging and milling. The 3D reconstruction of lipid droplet was performed using an AMIRA 7.0 software (Thermo Fisher Scientific).

Preparation of BSA-conjugated oleate acid. Firstly, 10% BSA solution (fatty acid free) in 150 mM NaCl was prepared at 37°C. The BSA completely was dissolved during stirring. Oleate was dissolved in 150 mM NaCl to a final concentration of 50 mM, heated during stirring at 70°C until dissolved. A 0.2 mL aliquot was mixed with 1.2 mL of a 10% BSA solution at 37°C. After 30 minutes of stirring to allow clarification of the solution, 0.6ml of water was added to bring the final concentration to 5 mM oleate. The solutions were filtered and stored at -20°C.

PC Liposome preparation. PC liposomes were prepared by phosphatidylcholine in chloroform/methanol (2:1); drying them under a stream of N2; resuspending them in PBS buffer; and sonication. The liposome suspension should be slightly cloudy but translucent. Then cells were cultured with PC liposomes during oleate loading.

Lipolysis assay. Cells were loaded with 200 μM oleate for one day. Oleate was then removed from the medium and the cells were starved for one day in serum-free medium. Cellular glycerol was measured by using a glycerol colorimetric assay kit (Biovision, Cat# K630) following the manufacturer's instructions. Cells were homogenized with 100 μL of glycerol assay buffer for 30 minutes on ice. The sample were centrifuged at 12000 rpm for 10 minutes. The supernatant was collected. 20 μL cell supernatant were mixed with 30 μL of glycerol assay buffer and 50 μL of a reaction mixture, and then incubated for 30 minutes, protect from light. Measure OD 570 nm for the colorimetric assay in a microtiter plate reader.

***In vivo* administration of Phosphatidylcholine.** Egg phosphatidylcholine (Avanti, 840051P) was dissolved with Mili-Q water. *p53*$^{+/+}$ and *p53*$^{-/-}$ mice were fed with choline-deficient diets for 5 weeks and orally gavage with vehicle or 300 μL 10 mg/ml of egg PC daily for the last 3 weeks.

Primary Hepatocyte Isolation, Culture, and Treatment. Primary hepatocytes were isolated from 8-9 weeks old male mice. Briefly, mice were perfused with 50 mL HBSS containing 0.25 mM EGTA through the inferior vena cava until the liver was blanched. The liver was then digested 5 mg of Type IV collagenase in an HBSS buffer supplemented with $CaCl_2$. The primary hepatocytes were seeded in cell culture dishes coated with 0.1% gelatin for 30min and air-dried,

supplemented with 1% Penicillin and Streptomycin, 2% FBS and 0.2% BSA in M199 for 4h. Then the culture was washed and changed lipid-free medium for12h. The cultured medium was used to culture HepG2 cells for proliferation and other assays.

Lipid droplet isolation. Briefly, liver tissues were rapidly homogenized in ice-cold buffer 10 mM Tris-HCl, pH7.4, 150 mM NaCl, 5 mM EDTA. Homogenize on ice ten times with a loose-fitting Dounce. Sucrose was added to final concentration of 65%. The liver homogenates were transferred into the bottom of the Beckman polycarbonate centrifuge tubes. 2 ml of buffer containing 20 mM HEPES, 100 mM KCl and 2 mM $MgCl_2$, (pH 7.4) was loaded on top of the homogenates. The tubes were then centrifuged in a pre-cooled Beckman SW41 swinging bucket rotor in a Beckman XP-100 ultracentrifuge at 25,700 × g at 4°C for 30 min. Carefully collect LDs from the top band of the gradient formed. The proteins in LDs were precipitated by ice-cold acetone, and washed twice with acetone/diethylether (1:1, vol/vol) and once with diethylether.

Liver tissue interstitial fluid extraction. Liver fresh tissues were cut into small pieces (1-3 mm^3) in PBS containing a protease and phosphatase inhibitor cocktail, transferred to 2 cm cell culture dish, avoiding as long as possible cell damage with minimum manipulation. Then, the tissue was eluted in 500 μL of PBS containing protease and phosphatase inhibitors (PBSi), and incubated at 37 °C in a CO_2 incubator for 2h, then centrifuged at 10000g for 5 min. Finally, the supernatant was collected and filtered (0.22 μm) to avoid cell contamination. The final sample, liver tissue interstitial fluid, was stored at -80 °C for LC-MS and other analysis assay.

Hematoxylin and Eosin Staining. Briefly, liver tissues were immediately collected and fixed in 4% formalin for 48 hours. Then liver tissues were dehydrated and embedded in paraffin, sectioned into 5 μm sections, and stained using hematoxylin and eosin to assess hepatic steatosis.

Immunohistology. Liver tissues were treated as Hematoxylin and Eosin Staining described. The 5 μm sections were dewaxed in Xylene for 30 minutes and rehydrated with sequential washes of diluted ethanol (100%, 95%, 80%) followed by water wash. Antigens were retrieved in sodium citrate buffer for 20 minutes in a 95°C-water bath. Sections were treated with 3% H_2O_2 for 10 minutes to block endogenous peroxidase activity, washed in PBS for10 minutes, then blocked in sheep serum for 1 hour. Primary antibody was incubated at 4°C overnight and washed in PBS. Secondary antibody was incubated for 1 hour at room temperature. Primary antibody positive cells were visualized using DAB(3,3′-diaminobenzidine) and counterstained with hematoxylin. Immunohistology images were acquired on an Olympus FV3000 microscope (Olympus) and imaged at 20× magnification.

Triglyceride and Phosphatidylcholine Extraction and Quantification. Liver tissues were weighed 50 mg, then homogenized in 1 mL ice-cold chloroform/methanol (2:1 v/v), and vortexed vigorously for 30 seconds, then a 1/4 volume of Mili-Q water was added. After centrifugation at 3000g for 10 minutes, the organic phase was collected, transfer equal volumes of the bottom chloroform layer to a new tube and under vacuum in a rotary evaporator. The triglyceride pellet was resuspended in 200 μL 5% Nonidet P40 in ddH_2O. The supernatant was heated at 95°C for 5 minutes and vortexed for 30 seconds, used to measure triglyceride and phosphatidylcholine level using the Triglyceride Quantification Colorimetric/Fluorometric Kit (Biovision, Cat# K622) and Phosphatidylcholine Colorimetric/Fluorometric Assay Kit (Biovision, Cat# K576) based on the manufacturer's instruction. Finally, tissue triglyceride and

phosphatidylcholine levels were normalized to tissue weight.

ALT, AST, ALP activity measurement. Serum ALT activity was measured with Alanine Aminotransferase Activity Colorimetric/Fluorometric Assay Kit (Biovision, Cat# K752); AST activity was calculated by Aspartate Aminotransferase Activity Colorimetric Assay Kit (Biovision, Cat# K753); ALP was measured with Alkaline Phosphatase Activity Colorimetric Assay Kit (Biovision, Cat# K412) the manufacturer's instruction.

Fig. S1

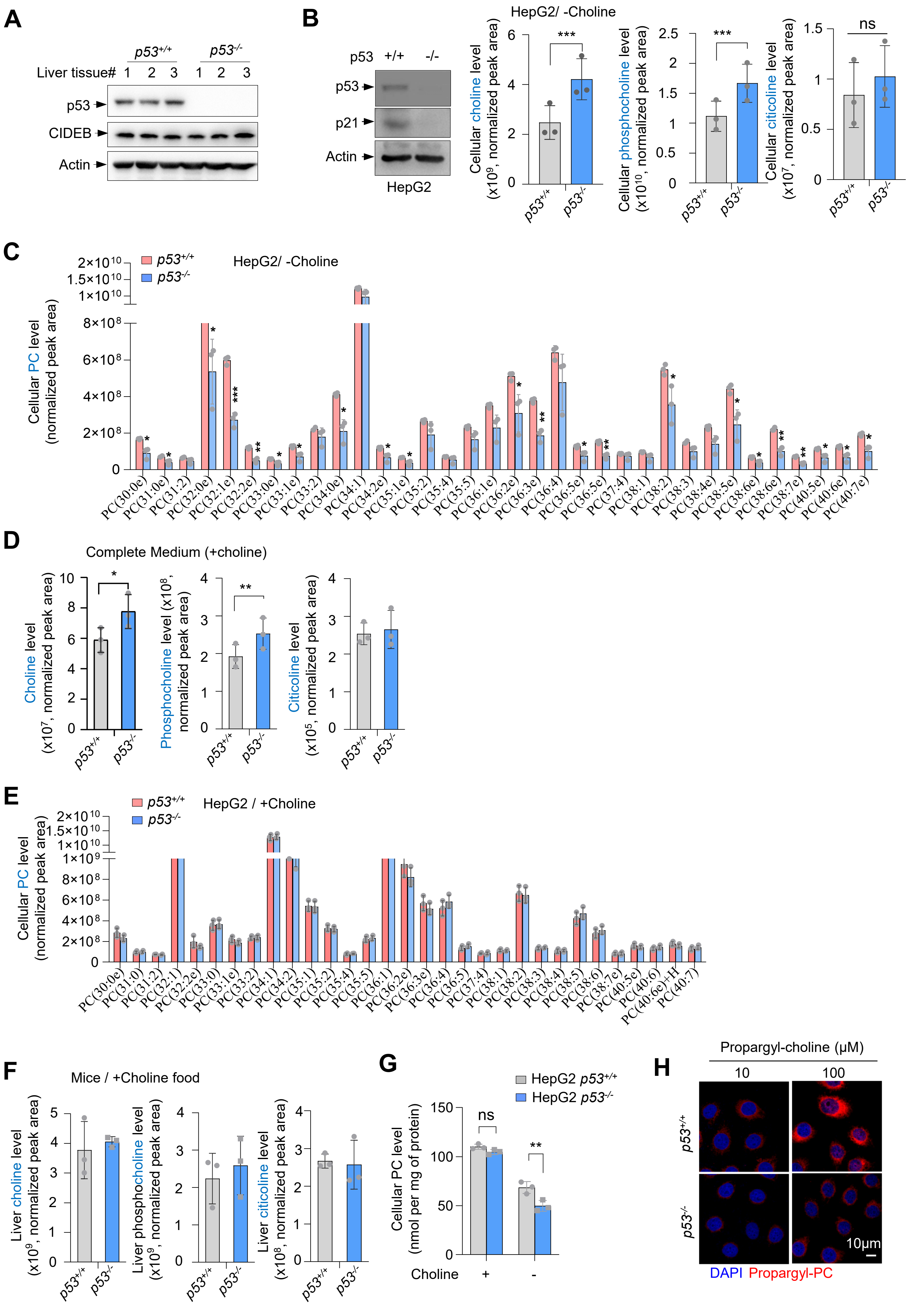
A
p53+/+
p53-/-
Liver tissue# 1 2 3 1 2 3
p53
CIDEB
Actin
B
p53 +/+ -/-
p53
p21
Actin
HepG2
HepG2/ -Choline
Cellular choline level (x10^9, normalized peak area)
Cellular phosphocholine level (x10^10, normalized peak area)
Cellular citicoline level (x10^7, normalized peak area)
ns
C
HepG2/ -Choline
Cellular PC level (normalized peak area)
D
Complete Medium (+choline)
Choline level (x10^7, normalized peak area)
Phosphocholine level (x10^8, normalized peak area)
Citicoline level (x10^5, normalized peak area)
E
HepG2 / +Choline
Cellular PC level (normalized peak area)
F
Mice / +Choline food
Liver choline level (x10^9, normalized peak area)
Liver phosphocholine level (x10^9, normalized peak area)
Liver citicoline level (x10^8, normalized peak area)
G
HepG2 p53+/+
HepG2 p53-/-
Cellular PC level (nmol per mg of protein)
Choline + -
H
Propargyl-choline (μM)
10 100
10μm
DAPI Propargyl-PC

Supplemental Figure 1. p53 deficiency reprograms the Kennedy pathway. (**A**) Related to Figure 1**B** and **C**. RNA-seq of liver tissue from *p53*$^{+/+}$ and *p53*$^{-/-}$ mice maintained on a choline-free diet for 4 weeks. Liver tissue was analyzed by Western blot. (**B**) *p53*$^{+/+}$ and *p53*$^{-/-}$ HepG2 cells cultured in choline-free medium for 48 hours, and relative levels (normalized peak areas) of choline, phosphocholine and citicoline were determined by LC-MS analysis (n=3 samples per treatment). Protein expression was analyzed by western blot. (**C**) Normalized peak areas of different PC species from *p53*$^{+/+}$ and *p53*$^{-/-}$ HepG2 cells cultured in choline-free medium for 48 hours (n=3 samples per treatment). (**D**) *p53*$^{+/+}$ and *p53*$^{-/-}$ HepG2 cells cultured in completed (choline-supplemented, unless otherwise indicated) medium for 48 hours, and normalized peak areas of choline, phosphocholine and citicoline are shown (n=3 samples per treatment). (**E**) Normalized peak areas of different PC species from *p53*$^{+/+}$ and *p53*$^{-/-}$ HepG2 cells cultured in completed medium for 48 hours (n=3 samples per treatment). (**F**) Normalized peak areas of choline, phosphocholine and citicoline in the livers of *p53*$^{+/+}$ and *p53*$^{-/-}$ mice maintained on a choline-supplemented diet for 8 weeks (n=3 mice per group). (**G**) Absolute cellular levels of PC in *p53*$^{+/+}$ and *p53*$^{-/-}$ HepG2 cells cultured in normal or choline-free medium for 48 hours (n=3 samples per treatment). (**H**) *p53*$^{+/+}$ and *p53*$^{-/-}$ HepG2 cells were cultured in choline-deficient medium containing 10 μM and 100 μM propargyl-choline for 12 hours. cells were then fixed and stained with 20 μM Alexa Fluor 647 fluorescent azide and DAPI (*please see methods for details*). Images were acquired on a confocal laser scanning microscope.

All data are the mean ± SD. **P*<0.05, ***P*<0.01, ****P*<0.001, based on two-tailed unpaired Student's t-test (**B**-**G**). n.s., not statistical significance.

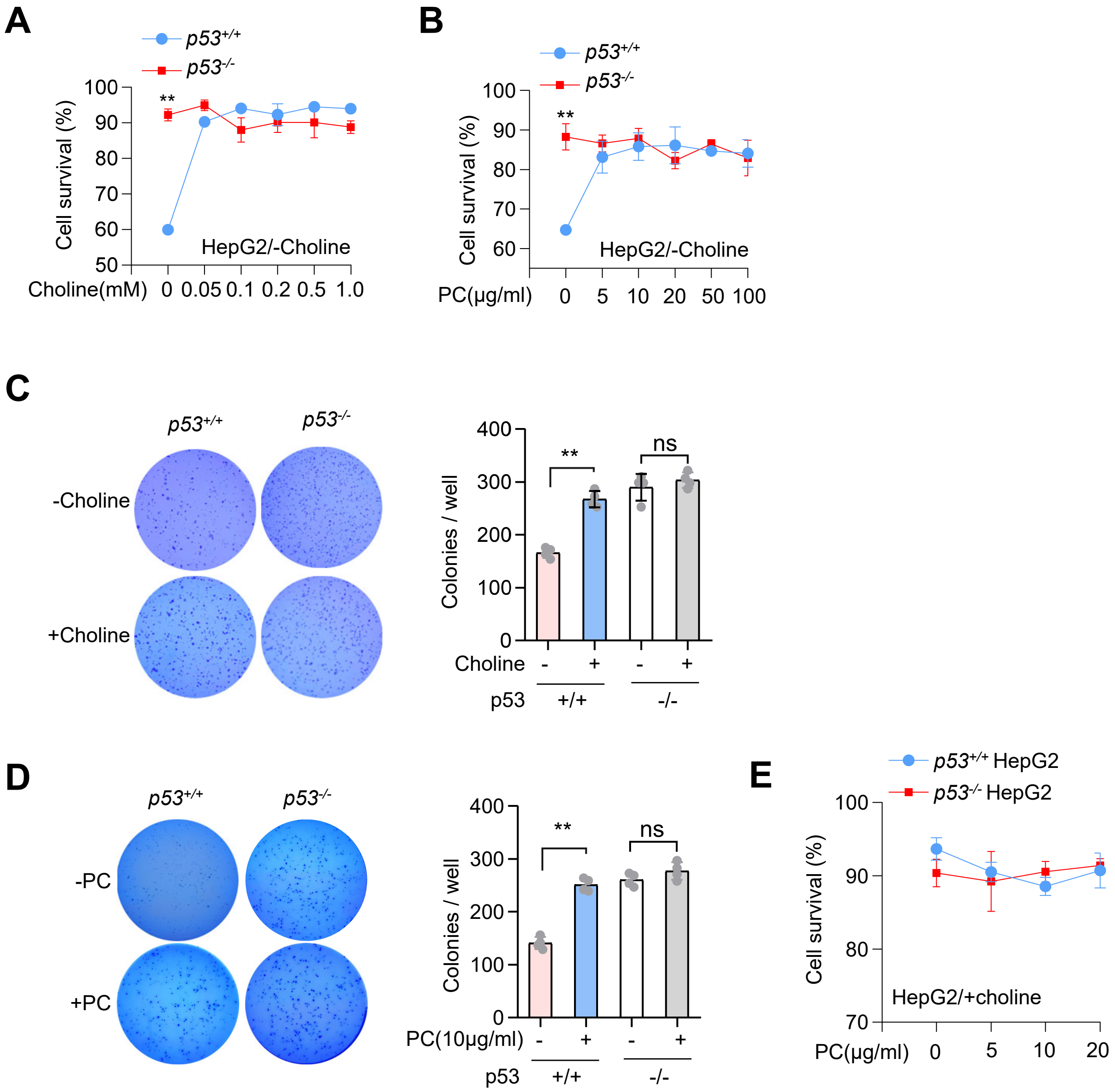
A
p53+/+
p53-/-
Cell survival (%)
HepG2/-Choline
Choline(mM) 0 0.05 0.1 0.2 0.5 1.0
**
B
p53+/+
p53-/-
Cell survival (%)
HepG2/-Choline
PC(μg/ml) 0 5 10 20 50 100
**
C
p53+/+
p53-/-
-Choline
+Choline
Colonies / well
**
ns
Choline - + - +
p53 +/+ -/-
D
p53+/+
p53-/-
-PC
+PC
Colonies / well
**
ns
PC(10μg/ml) - + - +
p53 +/+ -/-
E
p53+/+ HepG2
p53-/- HepG2
Cell survival (%)
HepG2/+choline
PC(μg/ml) 0 5 10 20

Supplemental Figure 2. Cells that lose p53 are resistant to choline deficiency. (**A**) Survival of *p53*$^{+/+}$ and *p53*$^{-/-}$ HepG2 cells cultured in choline-free medium for 12 hours and then treated without (0) or with increasing amounts of choline for another 72 hours. (**B**) Survival of *p53*$^{+/+}$ and *p53*$^{-/-}$ HepG2 cells cultured in choline-free medium in the absence (0) or presence of increasing amounts of L-α-PC for 72 hours. (**C**) Colony formation assay of *p53*$^{+/+}$ and *p53*$^{-/-}$ HepG2 cells cultured in complete (+choline) or choline-free (-choline) medium for 14 days. Numbers of colonies with a diameter greater than 20 μm were quantified (n=4 samples per treatment). (**D**) Images of colonies derived from *p53*$^{+/+}$ and *p53*$^{-/-}$ HepG2 cells cultured in choline-free medium in the absence or presence of 10 μg/ml L-α-PC for 14 days. (n=4 samples per treatment). Numbers of colonies with a diameter greater than 20 μm were quantified (n=4 samples per treatment). (**E**) Survival of *p53*$^{+/+}$ and *p53*$^{-/-}$ HepG2 cells cultured in complete medium in the absence (0) or presence of increasing amounts of L-α-PC for 72 hours. All data are the mean ± SD. Each experiment was carried out at least 3 independent times. *P<0.05, **P<0.01, ***P<0.001, based on two-tailed unpaired Student's t-test (**C** and **D**) and two-way ANOVA (**A**, **B**, and **E**). n.s., not statistical significance.

Fig. S3

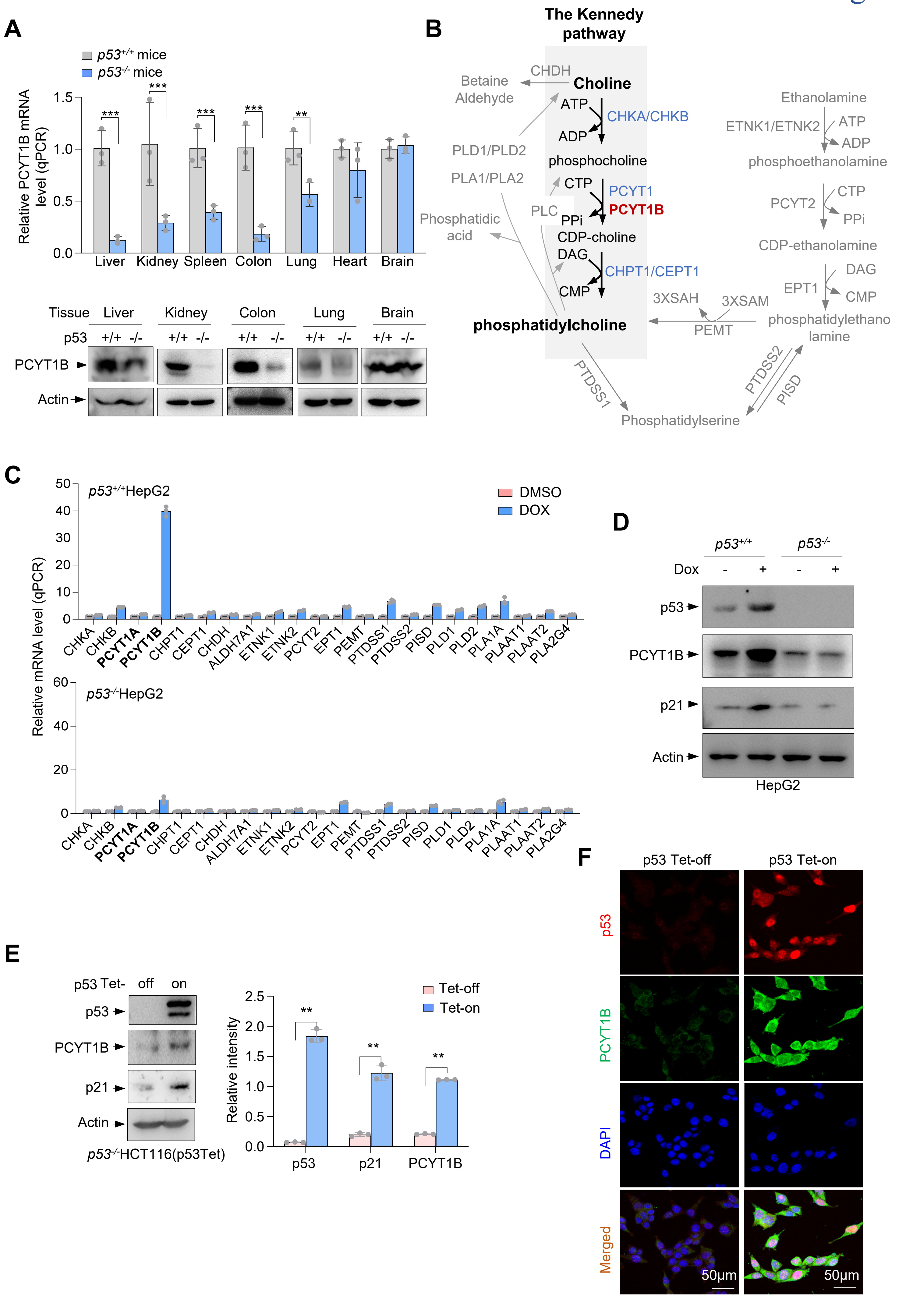
A
p53+/+ mice
p53-/- mice
Relative PCYT1B mRNA level (qPCR)
Liver Kidney Spleen Colon Lung Heart Brain
Tissue Liver Kidney Colon Lung Brain
p53 +/+ -/-
PCYT1B
Actin
B
The Kennedy pathway
Choline
CHDH
Betaine Aldehyde
ATP
ADP
CHKA/CHKB
PLD1/PLD2
PLA1/PLA2
phosphocholine
CTP
PPi
PCYT1
PCYT1B
PLC
Phosphatidic acid
CDP-choline
DAG
CMP
CHPT1/CEPT1
phosphatidylcholine
Ethanolamine
ETNK1/ETNK2
phosphoethanolamine
PCYT2
CDP-ethanolamine
EPT1
phosphatidylethanolamine
3XSAH
3XSAM
PEMT
PTDSS1
PTDSS2
PISD
Phosphatidylserine
C
p53+/+HepG2
DMSO
DOX
Relative mRNA level (qPCR)
CHKA CHKB PCYT1A PCYT1B CHPT1 CEPT1 CHDH ALDH7A1 ETNK1 ETNK2 PCYT2 EPT1 PEMT PTDSS1 PTDSS2 PISD PLD1 PLD2 PLA1A PLAAT1 PLAAT2 PLA2G4
p53-/-HepG2
D
p53+/+
p53-/-
Dox
p53
PCYT1B
p21
Actin
HepG2
E
p53 Tet- off on
p53
PCYT1B
p21
Actin
p53-/-HCT116(p53Tet)
Tet-off
Tet-on
Relative intensity
p53 p21 PCYT1B
F
p53 Tet-off
p53 Tet-on
p53
PCYT1B
DAPI
Merged
50μm

Supplemental Figure 3. p53 positively regulates statistical expression in vivo and in vitro. (**A**) The relative expression of PCYT1B in various tissues of *p53*$^{+/+}$ and *p53*$^{-/-}$ mice (n=3 mice per group) was determined by quantitative RT-PCR and Western blot analysis, respectively. (**B**) Schematic depicting the Kennedy (CDP-choline) pathway and its related metabolic pathways. (**C** and **D**) *p53*$^{+/+}$ and *p53*$^{-/-}$ HepG2 cells were treated with 0.5 μg/ml doxorubicin (DOX) for 12 hours. mRNA was extracted for quantitative RT-PCR analysis of expression of PC metabolism related genes as indicated (**C**). Protein expression of PCYT1B, p53 and p21 was determined by western blot analysis (**D**). (**E** and **F**) *p53*$^{-/-}$ HCT116 cells engineered to conditionally express wild-type p53 (Tet-On p53) were treated with 0.5 μg/ml doxycycline for 72 hours. (**E**) Expression of PCYT1B, p53 and p21 was analyzed by quantitative RT-PCR and western blotting respectively. (**F**) Cells were subjected to immunostaining with the anti-p53 and anti-PCYT1B antibodies and followed by confocal immunofluorescence imaging. All data are the mean ± SD. **P<0.01, ***P<0.001, based on two-tailed unpaired Student's t-test (**A**, **C** and **E**).

Fig. S4

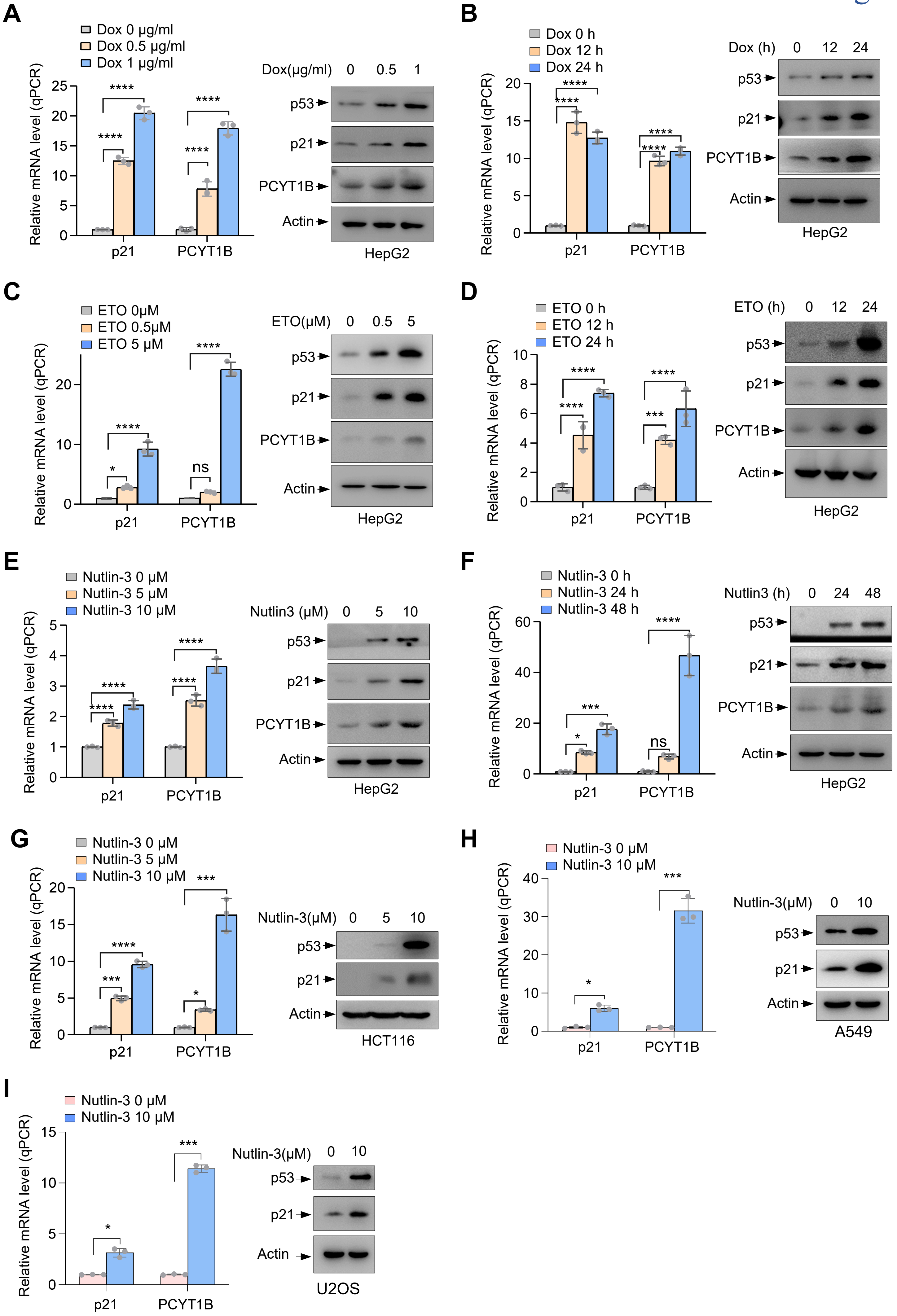

A
Dox 0 μg/ml
Dox 0.5 μg/ml
Dox 1 μg/ml
Relative mRNA level (qPCR)
p21
PCYT1B
Dox(μg/ml) 0 0.5 1
p53
p21
PCYT1B
Actin
HepG2
B
Dox 0 h
Dox 12 h
Dox 24 h
Relative mRNA level (qPCR)
p21
PCYT1B
Dox (h) 0 12 24
p53
p21
PCYT1B
Actin
HepG2
C
ETO 0μM
ETO 0.5μM
ETO 5 μM
Relative mRNA level (qPCR)
ns
p21
PCYT1B
ETO(μM) 0 0.5 5
p53
p21
PCYT1B
Actin
HepG2
D
ETO 0 h
ETO 12 h
ETO 24 h
Relative mRNA level (qPCR)
p21
PCYT1B
ETO (h) 0 12 24
p53
p21
PCYT1B
Actin
HepG2
E
Nutlin-3 0 μM
Nutlin-3 5 μM
Nutlin-3 10 μM
Relative mRNA level (qPCR)
p21
PCYT1B
Nutlin3 (μM) 0 5 10
p53
p21
PCYT1B
Actin
HepG2
F
Nutlin-3 0 h
Nutlin-3 24 h
Nutlin-3 48 h
Relative mRNA level (qPCR)
ns
p21
PCYT1B
Nutlin3 (h) 0 24 48
p53
p21
PCYT1B
Actin
HepG2
G
Nutlin-3 0 μM
Nutlin-3 5 μM
Nutlin-3 10 μM
Relative mRNA level (qPCR)
p21
PCYT1B
Nutlin-3(μM) 0 5 10
p53
p21
Actin
HCT116
H
Nutlin-3 0 μM
Nutlin-3 10 μM
Relative mRNA level (qPCR)
p21
PCYT1B
Nutlin-3(μM) 0 10
p53
p21
Actin
A549
I
Nutlin-3 0 μM
Nutlin-3 10 μM
Relative mRNA level (qPCR)
p21
PCYT1B
Nutlin-3(μM) 0 10
p53
p21
Actin
U2OS

Supplemental Figure 4. Activation of p53 leads to increased expression of PCYT1B in multiple cell lines. (**A** and **B**) HepG2 cells were treated with increasing amounts of DOX for 12 hours (**A**), or treated with 0.5 μg/ml DOX for indicated time points (**B**). Expression of PCYT1B and p21 was analyzed by quantitative RT-PCR and western blotting respectively. (**C** and **D**) HepG2 cells were treated with increasing amounts of etoposide (ETO) for 12 hours (**C**), or treated with 2.5 μM ETO for indicated time points (**D**). Expression of PCYT1B and p21 was analyzed by quantitative RT-PCR and western blotting respectively. (**E** and **F**) HepG2 cells were treated with increasing amounts of Nutlin-3 for 24 hours (**E**), or treated with 10 μM Nutlin-3 for indicated time points (**F**). Expression of PCYT1B and p21 was analyzed by quantitative RT-PCR and western blotting respectively. (**G-I**) Expression of PCYT1B in HCT116 cells (**G**), A549 cells (**H**) and U2OS cells (**I**) treated with indicated concentrations of Nutlin-3 for 24 hours was analyzed by quantitative RT-PCR and western blotting respectively. All data are the mean±SD. Each experiment was carried out at least 3 independent times. **P*<0.05, ***P*<0.01, ****P*<0.001, *****P*<0.0001, based on Two-way ANOVA followed by Dunnett's multiple-comparison test (**A**-**G**) or two-tailed unpaired Student's t-test (**H** and **I**).

Fig. S5

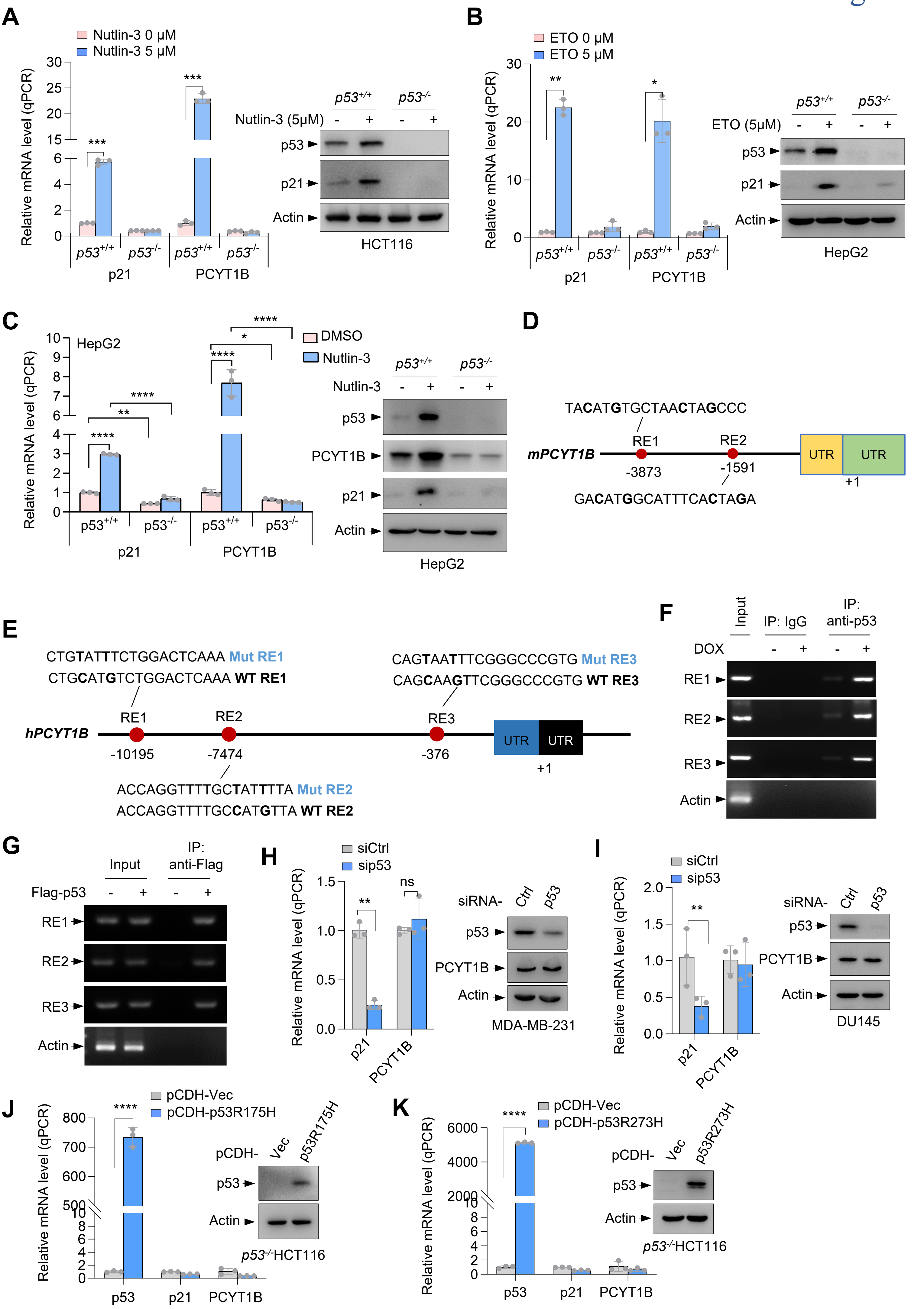
A
Nutlin-3 0 μM
Nutlin-3 5 μM
Relative mRNA level (qPCR)
p53+/+
p53-/-
p21
PCYT1B
Nutlin-3 (5μM)
p53
p21
Actin
HCT116
B
ETO 0 μM
ETO 5 μM
ETO (5μM)
HepG2
C
HepG2
DMSO
Nutlin-3
PCYT1B
D
TACATGTGCTAACTAGCCC
RE1
RE2
mPCYT1B
-3873
-1591
GACATGGCATTTCACTAGA
UTR
+1
E
CTGTATTTCTGGACTCAAA Mut RE1
CTGCATGTCTGGACTCAAA WT RE1
CAGTAATTTCGGGCCCGTG Mut RE3
CAGCAAGTTCGGGCCCGTG WT RE3
hPCYT1B
RE3
-10195
-7474
-376
ACCAGGTTTTGCTATTTTA Mut RE2
ACCAGGTTTTGCCATGTTA WT RE2
F
Input
IP: IgG
IP: anti-p53
DOX
G
IP: anti-Flag
Flag-p53
H
siCtrl
sip53
ns
siRNA-
Ctrl
MDA-MB-231
I
DU145
J
pCDH-Vec
pCDH-p53R175H
pCDH-
Vec
p53R175H
p53-/-HCT116
K
pCDH-p53R273H
p53R273H

Supplemental Figure 5. PCYT1B is a physiological target of wild-type P53, but not of mutant p53. (**A**) Expression of PCYT1B in *p53*$^{+/+}$ and *p53*$^{-/-}$ HCT116 cells treated with 5 μM Nutlin-3 or vehicle (0) for 48 hours was determined by quantitative RT-PCR. The knockout efficiency of p53 was verified by western blot analysis. (**B**) *p53*$^{+/+}$ and *p53*$^{-/-}$ HepG2 cells were treated with 5 μM ETO or vehicle (0) for 12 hours was determined by quantitative RT-PCR. Expression of PCYT1B, and the knockout efficiency of p53 were verified by western blot analysis. (**C**) *p53*$^{+/+}$ and *p53*$^{-/-}$ HepG2 cells were treated with DMSO or 10 μM Nutlin-3 for 24 hours. The mRNA levels of p21 and PCYT1B were analyzed by quantitative RT-PCR (left panel). Protein expression of p53, PCYT1B, p21 and actin were determined by western blot analysis (right panel). (**D**) Schematic representation of human PCYT1B genomic structure. The sequences of potential p53 response elements (REs) RE1-2 are shown. (**E**) Schematic representation of human PCYT1B genomic structure. The sequences of potential p53 response elements (REs) RE1-3 and the corresponding mutant REs are shown. (**F** and **G**) Chromatin immunoprecipitation (ChIP) assay for the binding of p53 to the potential p53 response element (PCYT1B-REs, reference Supplemental Figure 5**E** for detailed sequence) in *p53*$^{+/+}$ and *p53*$^{-/-}$ HepG2 cells treated without (-) or with 0.5 μg/ml DOX for 12 hours using anti-p53 DO-1 antibody (**F**). HEK293T cells transfected with control vector or Flag-p53 were analyzed by ChIP assay using anti-Flag antibody (**G**). Bound DNA was amplified by PCR and quantified. The results are representative of three independent experiments. (**H** and **I**) MDA-MB-231 cells (**H**) and DU145 cells (**I**) were transiently transfected with control siRNA or p53 siRNA for 48 hours. Expression of PCYT1B was determined by quantitative RT-PCR and western blot analysis respectively. The knockdown efficiency of p53 was verified by western blot analysis. Analysis of p21 expression was used as a positive control. (**J** and **K**) Expression of PCYT1B and p21 in *p53*$^{-/-}$ HCT116 cells stably expressing pCDH-vector control, pCDH-p53R175H (**J**) or pCDH-p53R273H (**K**) was analyzed by quantitative RT-PCR. The expression of mutant p53 was determined by western blot analysis. All data are the mean ± SD. Each experiment was carried out at least 3 independent times. ***P*<0.01, ****P*<0.001, *****P*<0.0001, based on Student's t-test (**A**, **B**, **H**, **I**, **J**, and **K**) or two-way ANOVA followed by Tukey's multiple-comparison test. n.s., not statistical significance.

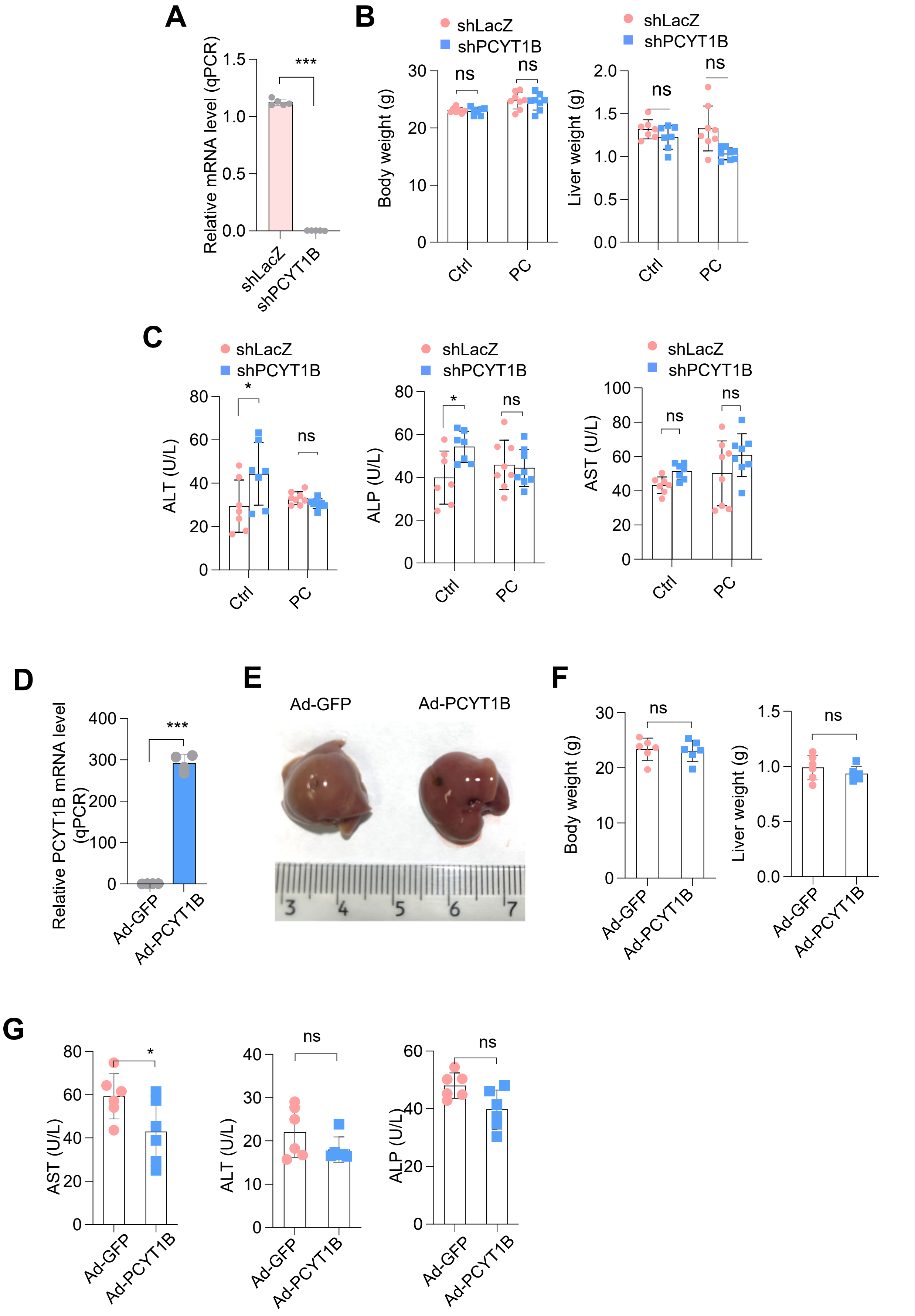
A
Relative mRNA level (qPCR)
1.5
1.0
0.5
0.0
***
shLacZ
shPCYT1B
B
shLacZ
shPCYT1B
Body weight (g)
30
20
10
0
ns
ns
Ctrl
PC
Liver weight (g)
2.0
1.5
1.0
0.5
0.0
ns
ns
C
ALT (U/L)
80
60
40
20
0
*
ns
ALP (U/L)
AST (U/L)
100
D
Relative PCYT1B mRNA level (qPCR)
400
300
200
100
***
Ad-GFP
Ad-PCYT1B
E
Ad-GFP
Ad-PCYT1B
3
4
5
6
7
F
Body weight (g)
Liver weight (g)
G
AST (U/L)
ALT (U/L)
ALP (U/L)
40
30
10

Supplemental Figure 6. PCYT1B improves liver function in mice on a choline-free diet. (**A-C**) Mice administrated with shLacZ or shPCYT1B adenoviruses for 10 days were maintained on a choline-deficient diet for 7 days. mRNA levels of PCYT1B in the liver tissues (**A**), the body weight and liver weight were measured (**B**). The activities of ALT, ALP and AST in serum were analyzed respectively(**C**). n=7 mice per group. (**D-G**) $p53^{-/-}$ mice administered with control (Ad-GFP) or (Ad-PCYT1B) virus for 14 days were maintained on a choline-free diet for 12 days. Expression of PCYT1B in the livers were determined by quantitative RT-PCR (**D**). Representative images of livers are shown (**E**). The body weight and liver weight (**F**), and serum activities of AST, ALT and ALP (**G**) were measured separately. Each group of n=6 mice. All data are the mean ± SD. **P*<0.05, ***P*<0.01, ****P*<0.001, based on two-tailed unpaired Student's t-test (**A-D**, **F**, and **G**). n.s., not statistical significance.

Fig. S7

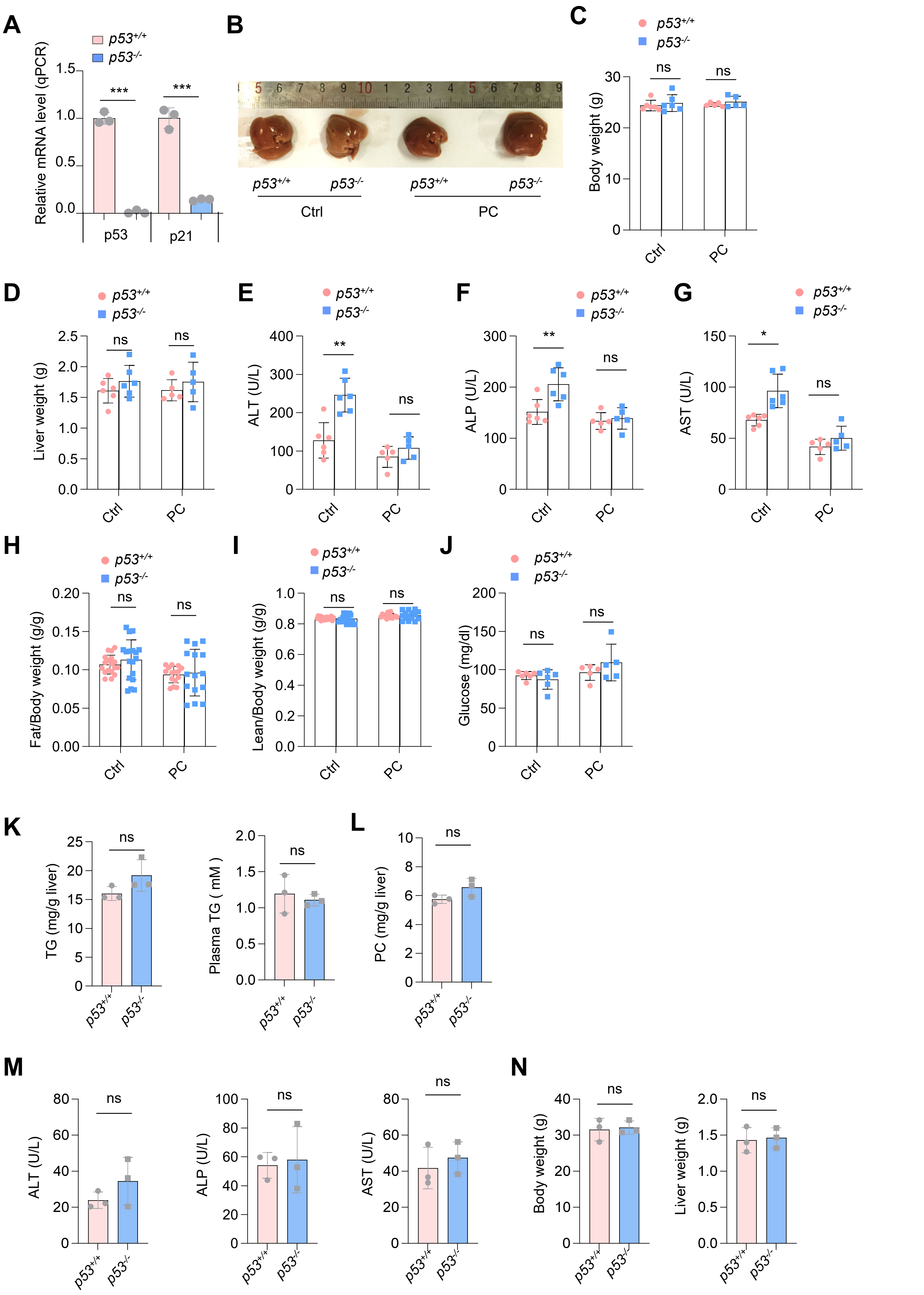

A
p53+/+
p53-/-
Relative mRNA level (qPCR)
***
p53
p21
B
Ctrl
PC
C
Body weight (g)
ns
D
Liver weight (g)
E
ALT (U/L)
**
F
ALP (U/L)
G
AST (U/L)
*
H
Fat/Body weight (g/g)
I
Lean/Body weight (g/g)
J
Glucose (mg/dl)
K
TG (mg/g liver)
Plasma TG (mM)
L
PC (mg/g liver)
M
N

Supplemental Figure 7. The regulation of liver function by p53 depends on the adequacy of PC or choline. (**A**) mRNA levels of p53 and p21 in the liver tissues from *p53*$^{+/+}$ and *p53*$^{-/-}$ mice maintained on a choline-free diet for 5 weeks were determined by quantitative RT-PCR analysis. (**B-J**) *p53*$^{+/+}$ and *p53*$^{-/-}$ mice were maintained on a choline-free diet for 5 weeks, with orally administered 300 μL PC (10 mg/mL) per day or not for the last 3 weeks. Representative images of livers are shown (**B**). The body weight (**C**), liver weight (**D**) and serum activities of ALT (**E**), ALP (**F**) and AST (**G**) were measured separately. The fat (**H**) and lean mass (**I**) normalized for body weight, and blood glucose levels (**J**) were also assessed. Each group of n=5 mice. (**K-N**) *p53*$^{+/+}$ and *p53*$^{-/-}$ mice were maintained on normal diet for 8 weeks. The liver TG and plasma TG levels (**K**) and the liver PC abundance (**L**) were measured. The serum activities of ALT, ALP and AST were examined (**M**). Body weight and liver weight were assessed (**N**). Each group of n=3 mice. All data are mean ± SD. *P<0.05, **P<0.01, based on two-tailed unpaired Student's t-test. n.s., not statistical significance.

Fig. S8

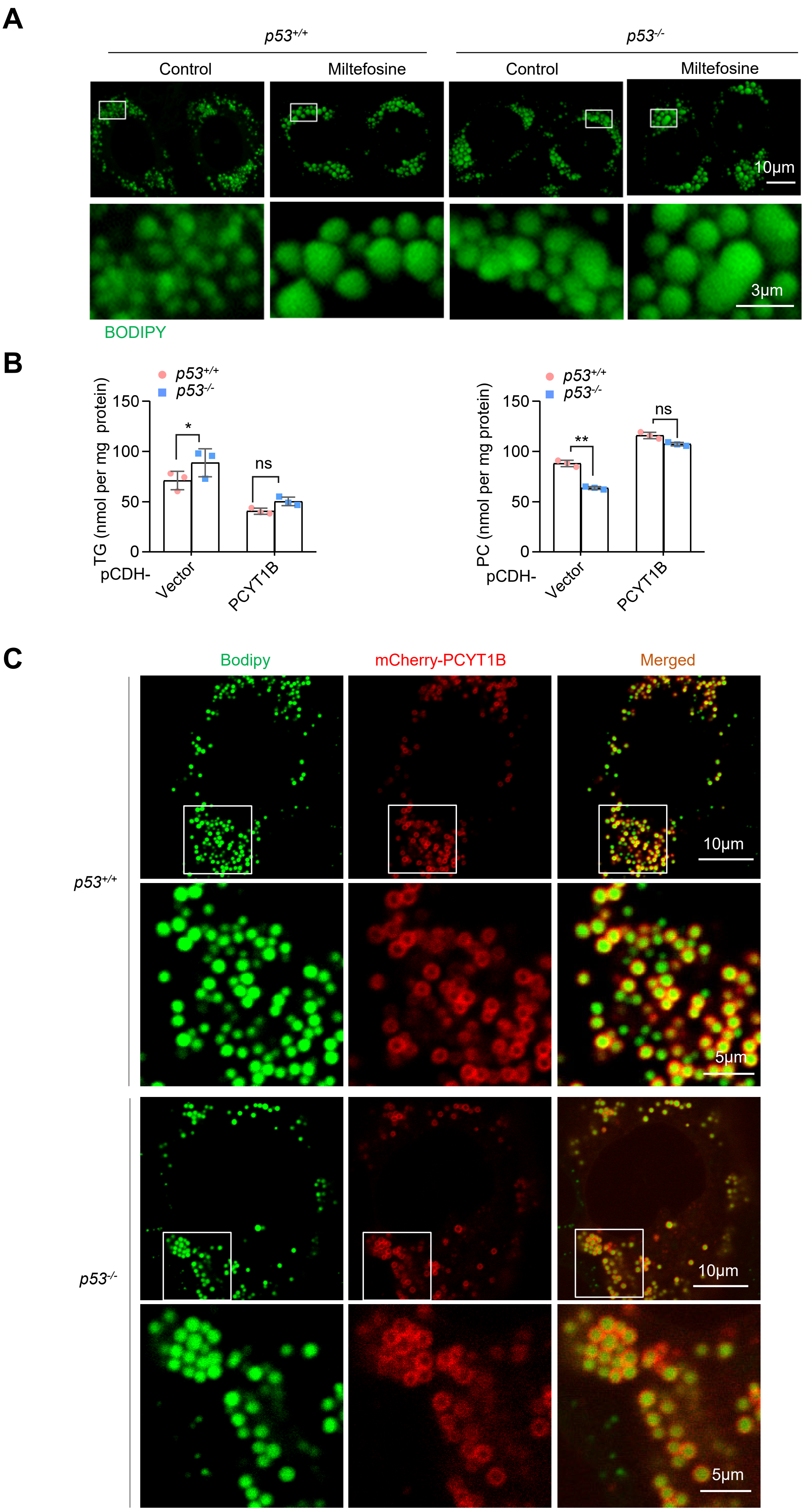
A
p53+/+
p53-/-
Control
Miltefosine
Control
Miltefosine
10μm
3μm
BODIPY
B
p53+/+
p53-/-
TG (nmol per mg protein)
150
100
50
0
*
ns
pCDH-
Vector
PCYT1B
PC (nmol per mg protein)
**
ns
C
Bodipy
mCherry-PCYT1B
Merged
p53+/+
p53-/-
10μm
5μm

Supplemental Figure 8. Upregulation of PCYT1B expression is required for p53-mediated suppression of LD growth. (**A**) *p53*$^{+/+}$ and *p53*$^{-/-}$ HepG2 cells treated with or without a PCYT1(also known as CCT) inhibitor Miltefosine were loaded with 200 μM oleate for 12 hours. Cells were stained with BODIPY 493/503 dye. (**B**) Related to Figure 4**B**. *p53*$^{+/+}$ and *p53*$^{-/-}$ HepG2 cells stably expressing PCYT1B or vector control were cultured with 200 μM oleate for 12 hours. TG and PC levels were measured. (**C**) *p53*$^{+/+}$ and *p53*$^{-/-}$ HepG2 cells stably expressing mCherry-PCYT1B fusion protein were cultured with 200 μM oleate for 12 hours. Cells were stained with BODIPY 493/503 dye. Enlarged areas are indicated. Scale bars are given. All data are mean ± SD. Each experiment was carried out at least 3 independent times. *P<0.05, **P<0.01, based on two-tailed unpaired Student's t-test (**B**). n.s., not statistical significance.

Fig. S9

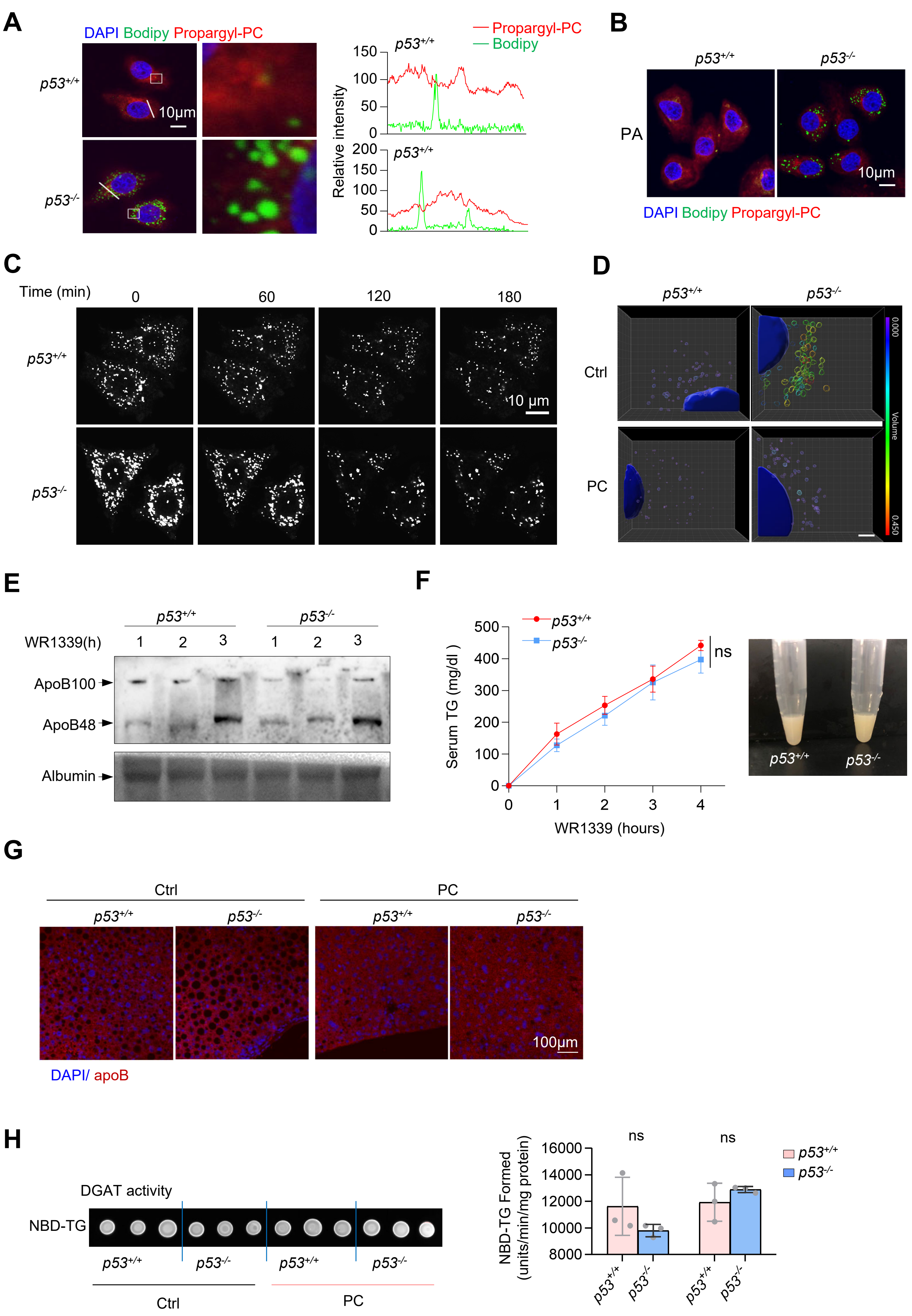

A
DAPI Bodipy Propargyl-PC
p53+/+
p53-/-
10μm
Propargyl-PC
Bodipy
Relative intensity
B
p53+/+
p53-/-
PA
10μm
DAPI Bodipy Propargyl-PC
C
Time (min)
0
60
120
180
10 μm
D
Ctrl
PC
Volume
E
WR1339(h)
ApoB100
ApoB48
Albumin
F
Serum TG (mg/dl)
WR1339 (hours)
ns
G
Ctrl
PC
100μm
DAPI/ apoB
H
DGAT activity
NBD-TG
NBD-TG Formed (units/min/mg protein)
ns

Supplemental Figure 9. Loss of p53 results in decreased PC levels and increased LDL growth, but has no effect on VLDL secretion. (**A**) *p53*$^{+/+}$ and *p53*$^{-/-}$ HepG2 cells cultured with 100 μM propargyl-choline for 12 hours in the presence of 200 μM oleate. Cells were stained with 20 μM Alexa Fluor 647 fluorescent azide, BODIPY, and DAPI. Images were acquired on a confocal laser scanning microscope. (**B**) *p53*$^{+/+}$ and *p53*$^{-/-}$ HepG2 cells cultured with 100 μM propargyl-choline in the presence of 200 μM palmitic acid (PA) for 12 hours. Cells were stained with 20 μM Alexa Fluor 647 fluorescent azide, BODIPY, and DAPI. Images were acquired on a confocal laser scanning microscope. (**C**) *p53*$^{+/+}$ and *p53*$^{-/-}$ HepG2 cells loaded with 200 μM oleate after 3 hours were then transfected with liposomal PC (*Please see Methods for details*) for different times. Cells were stained with BODIPY 493/503 dye at indicated time points. (**D**) *p53*$^{+/+}$ and *p53*$^{-/-}$ HepG2 cells cultured in choline-free medium supplemented with or without PC were loaded with 200 μM oleate for 12 hours. LDs were imaged and analyzed with a Focused Ion Beam Scanning Electron Microscopy combined with 3D reconstruction. (**E**) *p53*$^{+/+}$ and *p53*$^{-/-}$ mice maintained on a choline-free diet for 7 days were injected with Triton WR-1339 for different times, and blood was collected at indicated durations. Levels of plasma apoB-100/-48 were detected by western blotting. (**F**) *p53*$^{+/+}$ and *p53*$^{-/-}$ mice maintained on a choline-free diet for 7 days were injected with 500 mg/kg body weight of Triton WR-1339. Serum TG levels at the indicated durations post injection were measured. Plasma samples at 4 hours after Triton WR-1339 injection were imaged. Each group of n=5 mice. (**G**) Immunostaining of ApoB in liver tissues from *p53*$^{+/+}$ and *p53*$^{-/-}$ mice maintained on a choline-free diet for 5 weeks with or without oral administration of 300 μL PC (10 mg/ml) daily for the last three weeks. (**H**) *p53*$^{+/+}$ and *p53*$^{-/-}$ mice were treated as in (**G**). The DGAT activity in the murine liver tissues was assessed. Each group of n=3 mice. All data are mean±SD. Each experiment was performed at least 3 independent times. n.s., not statistical significance, based on two-way ANOVA (**F**) and Student's t-test (**H**).

Fig. S10

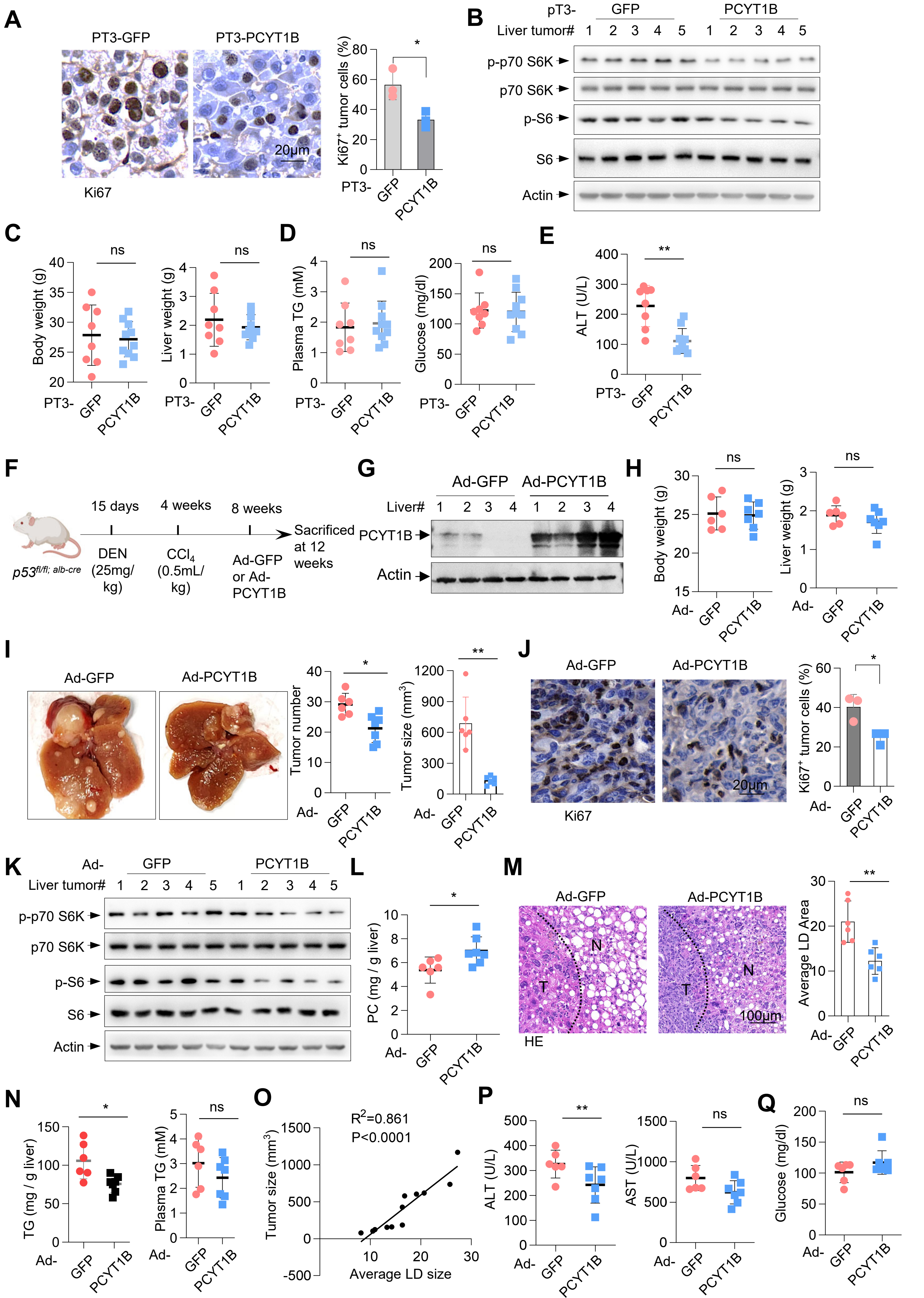
A
PT3-GFP
PT3-PCYT1B
20μm
Ki67
Ki67+ tumor cells (%)
PT3- GFP PCYT1B
B
pT3- GFP PCYT1B
Liver tumor# 1 2 3 4 5 1 2 3 4 5
p-p70 S6K
p70 S6K
p-S6
S6
Actin
C
Body weight (g)
Liver weight (g)
ns
D
Plasma TG (mM)
Glucose (mg/dl)
E
ALT (U/L)
F
15 days
4 weeks
8 weeks
Sacrificed at 12 weeks
DEN (25mg/kg)
CCl4 (0.5mL/kg)
Ad-GFP or Ad-PCYT1B
p53fl/fl; alb-cre
G
Ad-GFP
Ad-PCYT1B
Liver# 1 2 3 4 1 2 3 4
PCYT1B
Actin
H
Ad- GFP PCYT1B
I
Tumor number
Tumor size (mm3)
J
Ki67
K
L
PC (mg / g liver)
M
HE
100μm
T
N
Average LD Area
N
TG (mg / g liver)
O
R2=0.861
P<0.0001
Tumor size (mm3)
Average LD size
P
AST (U/L)
Q

Supplemental Figure 10. PCYT1B suppresses hepatic tumorigenesis during choline starvation. (**A**-**E**) Related to Figure 5**C**. PCYT1B suppresses liver tumorigenesis in response to oncogenic stress in *p53$^{fl/fl;alb\text{-}cre}$* mice. The liver tumor models using HTVI to deliver a transposon expressing MYC to the liver together with a PT3 plasmid that encodes GFP or PCYT1B. Mice were fed on a choline-free diet 4 weeks after injection with the components as indicated using HTVI and maintained for another 4 weeks. (**A**) Representative histological analysis of the tumors stained for Ki67. Liver tissues were analyzed by Western blot for the expression of indicated proteins. The activity of the mTORC signaling pathway was determined by Western blot analysis of the phosphorylation levels of its substrates S6K and S6 (**B**). Mice body and liver weight (**C**), The levels of plasma TG and blood glucose (**D**), as well as serum activity of ALT (**E**) were assessed. (**F**-**Q**) *p53$^{fl/fl;alb\text{-}cre}$* mice were treated with DEN 15 days after birth and CCl_4 2 weeks later. Mice were then infected with Ad-GFP or Ad-PCYT1B virus at week 8 and started on a choline-free diet, and euthanized after another 4 weeks (**F**). Expression of PCYT1B in livers were analyzed by western blot (**G**). The body and liver weight (**H**), representative images of liver tumor and size of tumors in livers (**I**), representative histological analysis of the tumors stained for Ki67 (**J**) are given. The activity of the mTORC signaling pathway was determined by Western blot analysis of the phosphorylation levels of its substrates S6K and S6 (**K**). Absolute levels of PC in the livers (**L**), the levels of plasma TG and liver TG (**N**), serum activities of ALT and AST(**P**), and blood glucose levels (**Q**) are assessed. n=6 (for Ad-GFP treatment) or 7 (for Ad-PCYT1B treatment) mice per group. (**M**) Representative histological analysis of the tumors stained for H&E, and the LD area were quantified. Images and quantification were taken at tumor periphery. (**O**) Linear regression analysis was performed on the mean LD size and tumor size in the livers tested (n = 12). All data are the mean±SD. **P*<0.05, ***P*<0.01, based on two-tailed unpaired Student's t-test (**A-E**, **H-J**, **L-N**, **P**, and **Q**) and linear regression analysis (**O**). n.s., not statistical significance.

Fig. S11

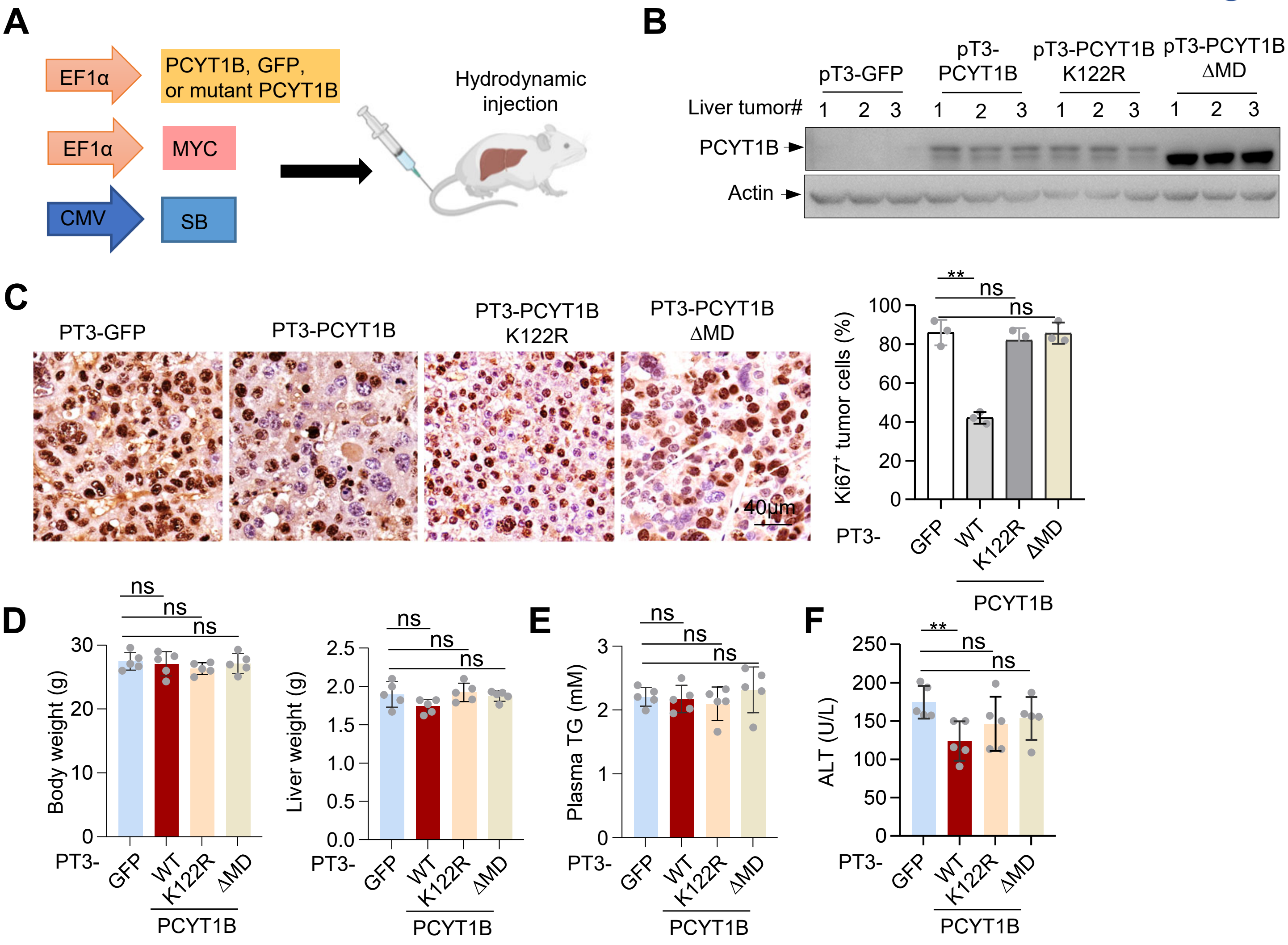

A
EF1α
PCYT1B, GFP, or mutant PCYT1B
EF1α
MYC
CMV
SB
Hydrodynamic injection
B
pT3-GFP
pT3-PCYT1B
pT3-PCYT1B K122R
pT3-PCYT1B ΔMD
Liver tumor# 1 2 3 1 2 3 1 2 3 1 2 3
PCYT1B
Actin
C
PT3-GFP
PT3-PCYT1B
PT3-PCYT1B K122R
PT3-PCYT1B ΔMD
40μm
Ki67+ tumor cells (%)
**
ns
ns
PT3-
GFP
WT
K122R
ΔMD
PCYT1B
D
Body weight (g)
Liver weight (g)
E
Plasma TG (mM)
F
ALT (U/L)

Supplemental Figure 11. The enzymatic activity of PCYT1B is required for its tumor suppression function. (**A-F**) A liver tumor model using HTVI to deliver a transposon expressing MYC together with a PT3 plasmid encoding GFP, wild-type PCYT1B or enzymatically inactive mutant PCYT1B to the liver (**A**). The expression of PCYT1B in liver tumor tissue was analyzed by Western blot (**B**). Representative histological analyses of tumors stained for Ki67 are shown (**C**). Body and liver weights (**D**), plasma TG levels (**E**) and serum ALT activities (**F**) were assessed. n=5 mice for each group. All data are the mean±SD. *P<0.05, **P<0.01, based on one-way ANOVA followed by Dunnett's multiple-comparison test. n.s., not statistical significance.

Fig. S12

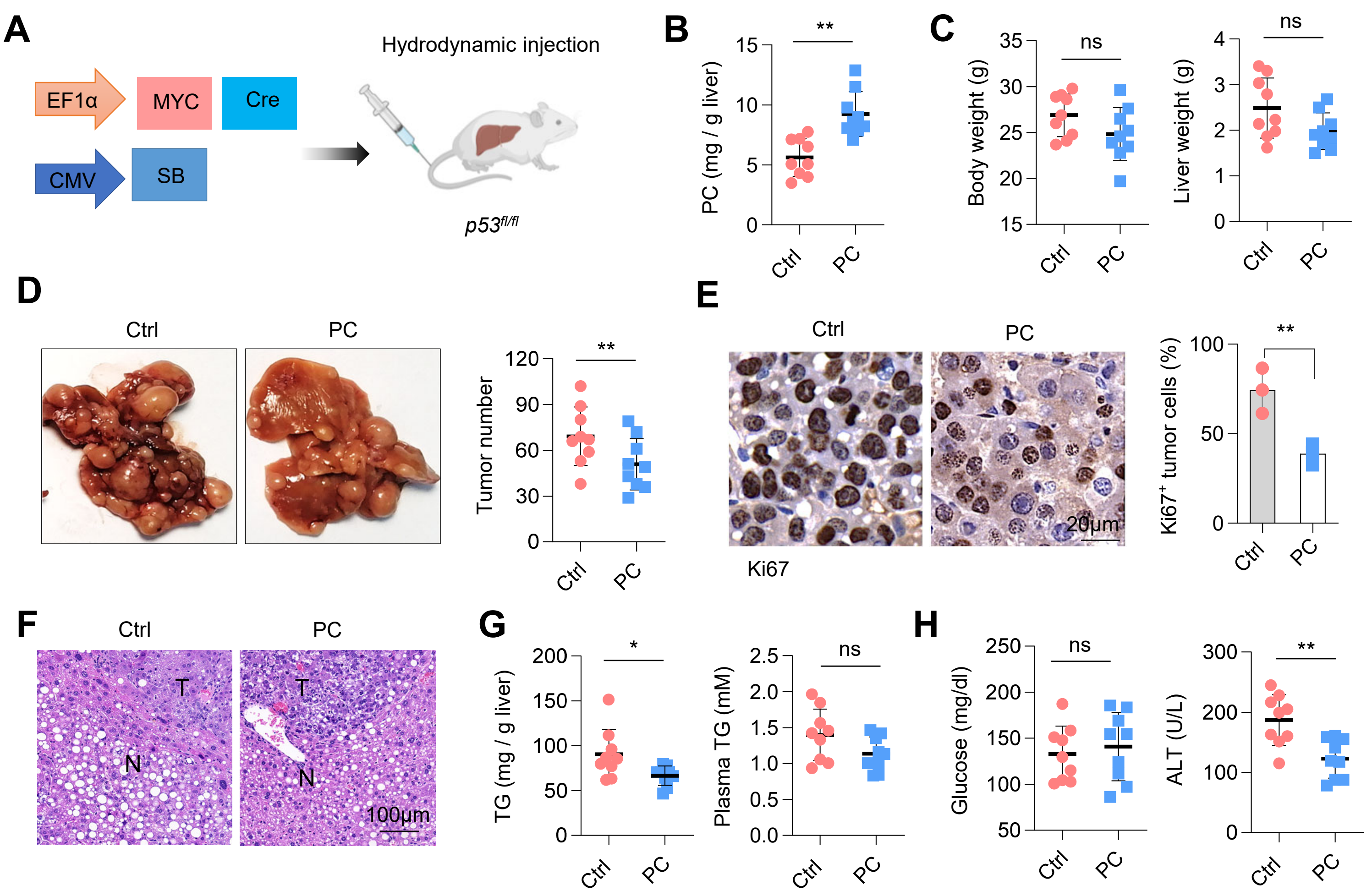
A
EF1α
MYC
Cre
CMV
SB
Hydrodynamic injection
p53fl/fl
B
PC (mg / g liver)
**
Ctrl
PC
C
Body weight (g)
ns
Liver weight (g)
ns
D
Ctrl
PC
Tumor number
**
E
Ctrl
PC
20μm
Ki67
Ki67+ tumor cells (%)
**
F
Ctrl
PC
T
N
100μm
G
TG (mg / g liver)
*
Plasma TG (mM)
ns
H
Glucose (mg/dl)
ns
ALT (U/L)
**

Supplemental Figure 12. PC suppresses tumorigenesis. (**A**-**H**) PC treatment suppresses oncogenic stress-induced liver tumorigenesis in $p53^{fl/fl}$ mice. (**A**) Diagram of PC treatment (oral administration in 100 mg/kg doses daily) in $p53^{fl/fl;alb\text{-}cre}$ mice injected with the indicated components required for generating liver tumors (p53 loss; MYC) using HTVI. Mice were fed on a choline-free diet 4 weeks after injection and maintained for another 4 weeks. (**B**) The levels of liver PC were measured. (**C**) Body and liver weight were assessed. (**D**) Representative images of liver tumor multiplicity are shown and the number of tumors was measured. n=9 mice per group. (**E**) Representative histological analysis of the tumors stained for Ki67. (**F**) Representative histological analysis of the tumors stained for H&E. Images and quantification were taken at tumor periphery. The liver TG and plasma TG contents (**G**), and the levels of blood glucose and serum activity of ALT (**H**) were assessed. All data are mean±SD. *$P<0.05$, **$P<0.01$, based on two-tailed unpaired Student's t-test. n.s., not statistical significance.

Fig. S13

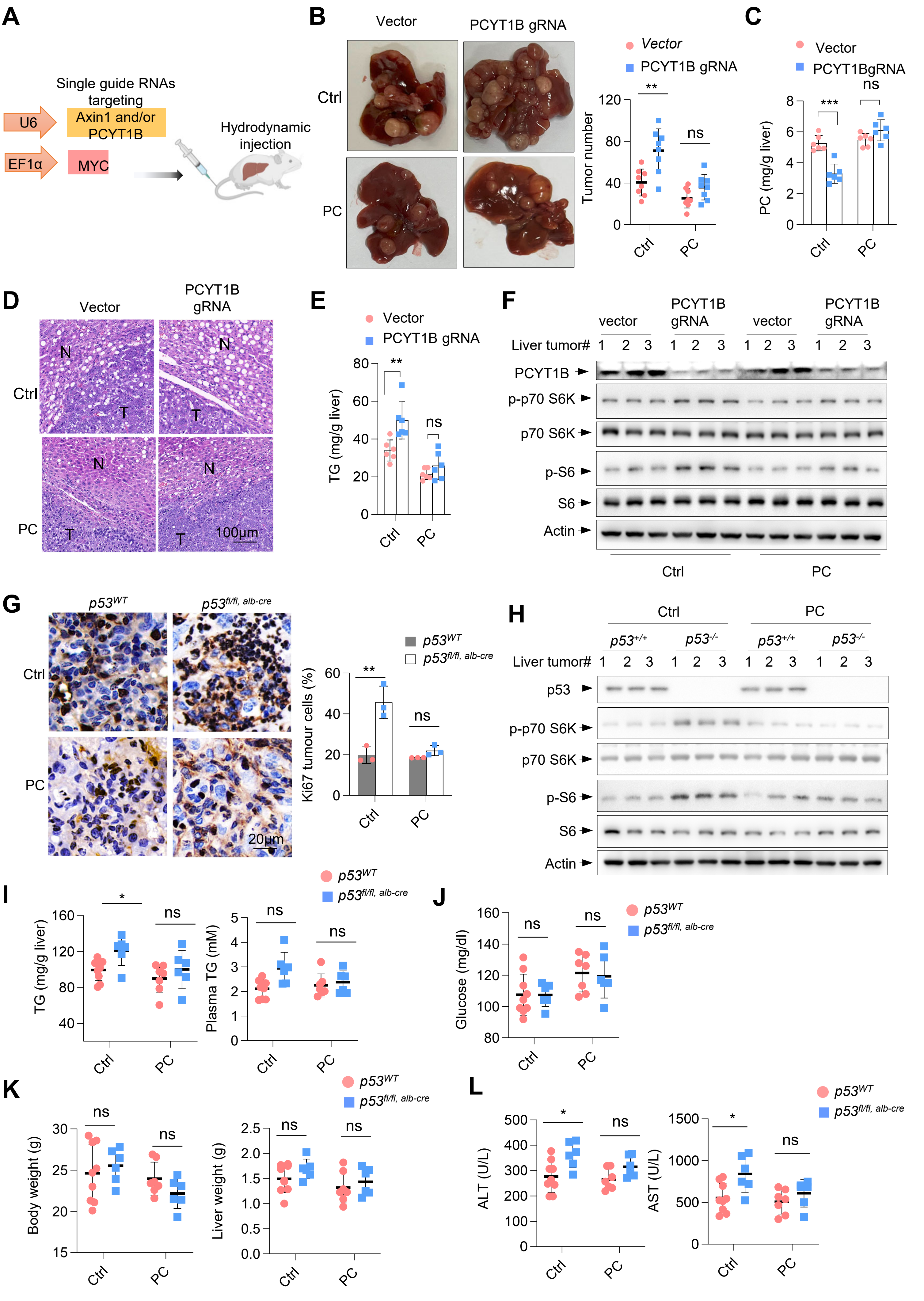
A
Single guide RNAs targeting
U6
Axin1 and/or PCYT1B
EF1α
MYC
Hydrodynamic injection
B
Vector
PCYT1B gRNA
Ctrl
PC
Vector
PCYT1B gRNA
Tumor number
**
ns
C
Vector
PCYT1BgRNA
PC (mg/g liver)
***
ns
D
Vector
PCYT1B gRNA
Ctrl
PC
N
T
100μm
E
Vector
PCYT1B gRNA
TG (mg/g liver)
**
ns
F
vector
PCYT1B gRNA
Liver tumor#
PCYT1B
p-p70 S6K
p70 S6K
p-S6
S6
Actin
Ctrl
PC
G
p53WT
p53fl/fl, alb-cre
Ctrl
PC
20μm
Ki67 tumour cells (%)
**
ns
H
Ctrl
PC
p53+/+
p53-/-
Liver tumor#
p53
p-p70 S6K
p70 S6K
p-S6
S6
Actin
I
p53WT
p53fl/fl, alb-cre
TG (mg/g liver)
Plasma TG (mM)
*
ns
J
Glucose (mg/dl)
ns
K
Body weight (g)
Liver weight (g)
ns
L
ALT (U/L)
AST (U/L)
*
ns

Supplemental Figure 13. PC restoration is sufficient to suppress PCYT1B-depeted or p53-deficient tumorigenesis. (**A-F**) Hydrodynamic tail vein injection (HTVI) was performed in $p53^{+/+}$ mice to deliver a transposon expressing MYC together with a CRISPR plasmid targeting PCYT1B or Axin1 (sgRNA) to the liver (**A**). Representative images of liver tumor multiplicity are shown and the number of tumors were measured (**B**). Absolute levels of PC (**C**) and TG (**E**) in the livers were measured. n=6 mice per group. (**D**) Representative histological analysis of H&E-stained tumors. (**F**) Liver tissues were analyzed by Western blot for the expression of indicated proteins. The activity of the mTORC signaling pathway was determined by the phosphorylation of its substrates S6K and S6. (**G-L**) $p53^{WT}$ and $p53^{fl/fl;alb\text{-}cre}$ mice were treated with DEN, CCl_4, PC and fed on a choline-free diet, as illustrated in Figure **6A**. n=6 mice per group. (**G**) Representative histological analysis of the tumors stained for Ki67. (**H**) Liver tissues were analyzed by Western blot for expression of the indicated proteins. The liver and plasma TG abundance (**I**), blood glucose levels (**J**), and serum activities of ALT and AST (**K**), body and liver weight (**L**) were assessed. All data are mean±SD. *P<0.05, **P<0.01, based on Student's t-test. n.s., not statistical significance.

Fig. S14

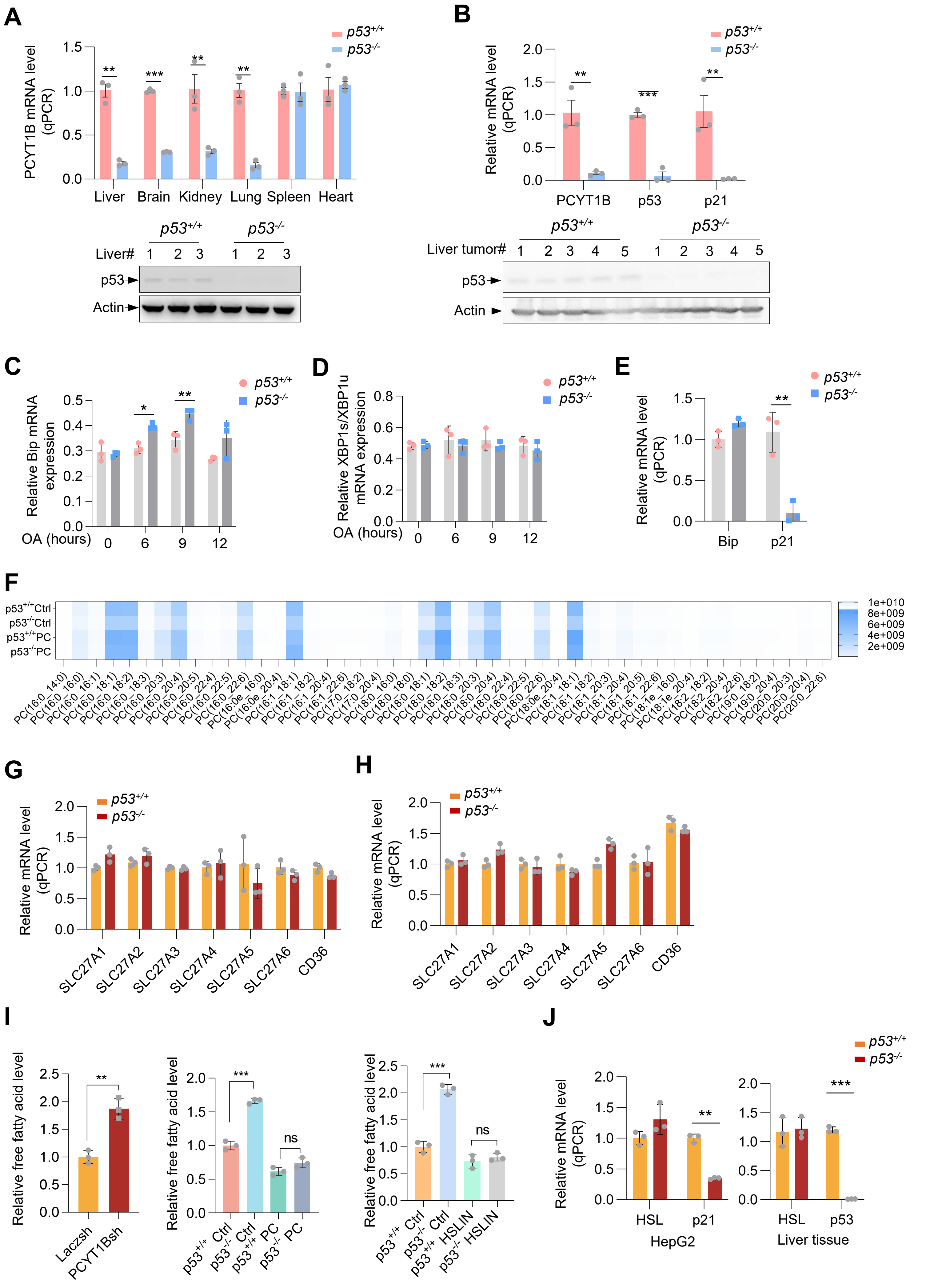
A
PCYT1B mRNA level (qPCR)
p53+/+
p53-/-
Liver
Brain
Kidney
Lung
Spleen
Heart
Liver#
p53
Actin
B
Relative mRNA level (qPCR)
PCYT1B
p53
p21
Liver tumor#
C
Relative Bip mRNA expression
OA (hours)
D
Relative XBP1s/XBP1u mRNA expression
E
Bip
p21
F
p53+/+Ctrl
p53-/-Ctrl
p53+/+PC
p53-/-PC
1e+010
8e+009
6e+009
4e+009
2e+009
PC(16:0_14:0)
PC(16:0_16:0)
PC(16:0_16:1)
PC(16:0_18:1)
PC(16:0_18:2)
PC(16:0_18:3)
PC(16:0_20:3)
PC(16:0_20:4)
PC(16:0_20:5)
PC(16:0_22:4)
PC(16:0_22:5)
PC(16:0_22:6)
PC(16:0e_16:0)
PC(16:0e_20:4)
PC(16:1_18:1)
PC(16:1_18:2)
PC(16:1_20:4)
PC(16:1_22:6)
PC(17:0_18:2)
PC(17:0_20:4)
PC(18:0_16:0)
PC(18:0_18:0)
PC(18:0_18:1)
PC(18:0_18:2)
PC(18:0_18:3)
PC(18:0_20:3)
PC(18:0_20:4)
PC(18:0_22:4)
PC(18:0_22:5)
PC(18:0_22:6)
PC(18:0e_20:4)
PC(18:1_18:1)
PC(18:1_18:2)
PC(18:1_20:3)
PC(18:1_20:4)
PC(18:1_20:5)
PC(18:1_22:6)
PC(18:1e_16:0)
PC(18:1e_20:4)
PC(18:2_18:2)
PC(18:2_20:4)
PC(18:2_22:6)
PC(19:0_18:2)
PC(19:0_20:4)
PC(20:0_20:3)
PC(20:0_20:4)
PC(20:0_22:6)
G
H
SLC27A1
SLC27A2
SLC27A3
SLC27A4
SLC27A5
SLC27A6
CD36
I
Relative free fatty acid level
Laczsh
PCYT1Bsh
p53+/+ Ctrl
p53-/- Ctrl
p53+/+ PC
p53-/- PC
p53+/+ HSLIN
p53-/- HSLIN
ns
J
HSL
HepG2
Liver tissue

Supplemental Figure 14. Mechanistic studies of how p53 loss-induced LD growth supports tumorigenesis. (**A** and **B**) mRNA levels of PCYT1B in multiple tissues of *p53*$^{+/+}$ and *p53*$^{-/-}$ mice shortly after DEN treatment (**A**), and in tumors formed in DEN -treated *p53*$^{+/+}$ and *p53*$^{-/-}$ mice (**B**) were analyzed by quantitative RT-PCR analysis. p53 expression was determined by western blot analysis. n=3 (**A**) or 5 (**B**) mice per group. (**C** and **D**) *p53*$^{+/+}$ and *p53*$^{-/-}$ HepG2 cells cultured in choline-free medium containing 200 μM oleate for indicated time points were analyzed by quantitative RT-PCR for the expression of Bip and XBP1s/XBP1u respectively. n=3 independent wells for each treatment. (**E**) Liver tissue from *p53*$^{+/+}$ and *p53*$^{-/-}$ mice fed a choline-free diet for 4 weeks was analyzed by quantitative RT-PCR for the expression of Bip. n=6 mice per group. (**F**) *p53*$^{+/+}$ and *p53*$^{-/-}$ mice were maintained on a choline-free diet for 4 weeks, with or without oral administration of 300 μL PC (10 mg/ml) daily. PC levels on lipid droplets were measured by LC-MS analysis. n=6 mice per group. (**G**) *p53*$^{+/+}$ and *p53*$^{-/-}$ HepG2 cells cultured in choline-free medium containing 200 μM oleate for 24 hours were analyzed by quantitative RT-PCR analysis for the expression of the indicated genes. n=3 independent wells for each treatment. (**H**) Liver tumor tissues from *p53*$^{+/+}$ and *p53*$^{-/-}$ mice feed on a choline-free diet for 4 weeks were analyzed by quantitative RT-PCR analysis for the indicated gene expression. n=6 mice per group. (**I**) Related to Figure **A, B** and **E**. Hepatocytes from mice fed a choline-deficient diet were cultured in lipid-free medium for 12 hours. Fatty acid levels in the culture medium were determined. n=3 samples per group. (**J**) HSL expression in *p53*$^{+/+}$ and *p53*$^{-/-}$ HepG2 cells or liver tissue from *p53*$^{+/+}$ and *p5*$^{3-/-}$ mice was determined by quantitative RT-PCR analysis. n=3 samples per group. All data are the mean±SD. $^*P<0.05$, $^{**}P<0.01$, based on Student's t-test (A-E, G-J). n.s., not statistical significance.

Fig. S15

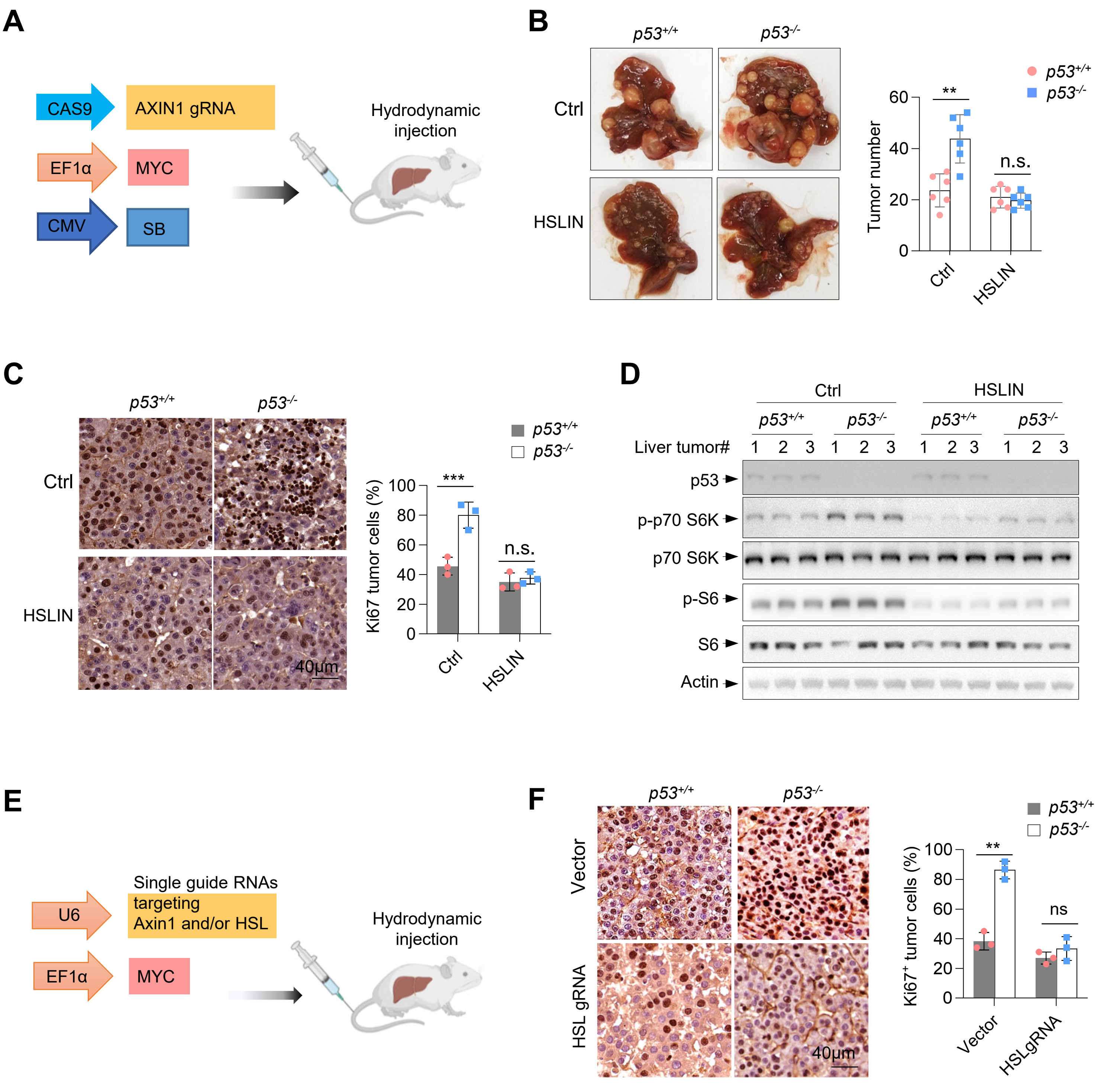
A
CAS9
AXIN1 gRNA
EF1α
MYC
CMV
SB
Hydrodynamic injection
B
p53+/+
p53-/-
Ctrl
HSLIN
Tumor number
**
n.s.
C
Ki67 tumor cells (%)
***
40μm
D
Liver tumor#
1 2 3 1 2 3 1 2 3 1 2 3
p53
p-p70 S6K
p70 S6K
p-S6
S6
Actin
E
U6
Single guide RNAs targeting Axin1 and/or HSL
EF1α
MYC
Hydrodynamic injection
F
Vector
HSL gRNA
Ki67+ tumor cells (%)
ns
HSLgRNA

Supplemental Figure 15. Effect of HSL on the development of tumors harboring p53 deletion. (**A-D**) Diagram showing the generation of a liver tumor model using HTVI to deliver a transposon expressing MYC to the liver along with a CRISPR plasmids that that directly targets Axin1. Mice were orally administrated with 100 μL HSL inhibitor (HSL-IN-1, a HSL inhibitor, 5 mg/ml) daily (**A**). (**B**) Representative images of liver tumor multiplicity are shown and the number of tumors was measured. (**C**) Representative histological analysis of Ki67-stained tumors. (**D**) Liver tissues were analyzed by Western blot for the expression of indicated proteins. n=6 mice per group. (**E** and **F**) Diagram showing the generation of a liver tumor model using HTVI to deliver a transposon expressing MYC to the liver along with a CRISPR plasmids that that directly targets HSL and/or Axin1(**E**). (**F**) Representative histological analysis of the tumors stained for Ki67. All data are mean±SD. **P*<0.05, ***P*<0.01, based on Student's t-test. n.s., not statistical significance.

Fig. S16

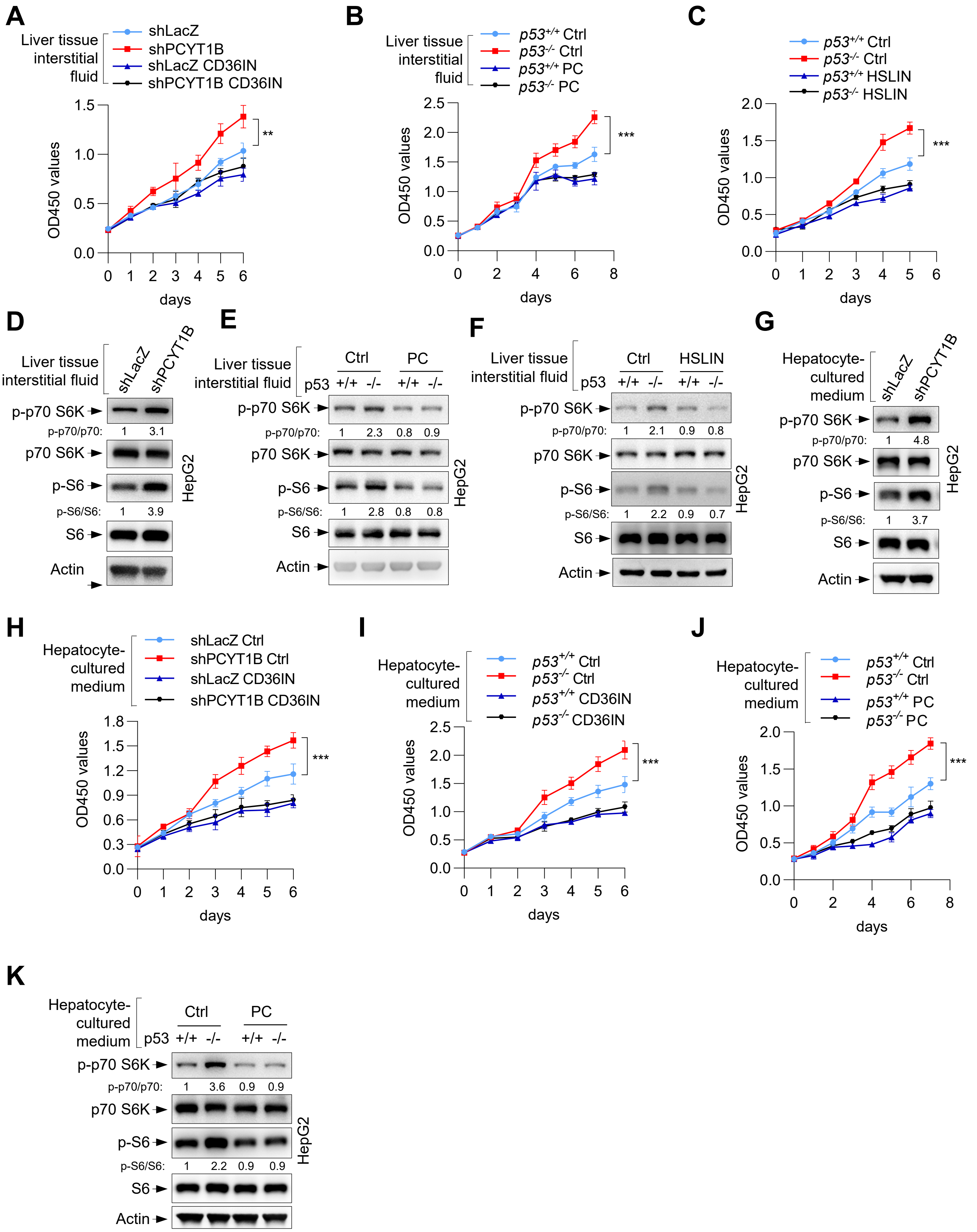
A
Liver tissue interstitial fluid
shLacZ
shPCYT1B
shLacZ CD36IN
shPCYT1B CD36IN
OD450 values
days
**
B
Liver tissue interstitial fluid
p53+/+ Ctrl
p53-/- Ctrl
p53+/+ PC
p53-/- PC
OD450 values
days
***
C
p53+/+ Ctrl
p53-/- Ctrl
p53+/+ HSLIN
p53-/- HSLIN
OD450 values
days
***
D
Liver tissue interstitial fluid
shLacZ
shPCYT1B
p-p70 S6K
p-p70/p70: 1 3.1
p70 S6K
p-S6
p-S6/S6: 1 3.9
S6
Actin
HepG2
E
Liver tissue interstitial fluid
Ctrl
PC
p53 +/+ -/- +/+ -/-
p-p70 S6K
p-p70/p70: 1 2.3 0.8 0.9
p70 S6K
p-S6
p-S6/S6: 1 2.8 0.8 0.8
S6
Actin
HepG2
F
Liver tissue interstitial fluid
Ctrl
HSLIN
p53 +/+ -/- +/+ -/-
p-p70 S6K
p-p70/p70: 1 2.1 0.9 0.8
p70 S6K
p-S6
p-S6/S6: 1 2.2 0.9 0.7
S6
Actin
HepG2
G
Hepatocyte-cultured medium
shLacZ
shPCYT1B
p-p70 S6K
p-p70/p70: 1 4.8
p70 S6K
p-S6
p-S6/S6: 1 3.7
S6
Actin
HepG2
H
Hepatocyte-cultured medium
shLacZ Ctrl
shPCYT1B Ctrl
shLacZ CD36IN
shPCYT1B CD36IN
OD450 values
days
***
I
Hepatocyte-cultured medium
p53+/+ Ctrl
p53-/- Ctrl
p53+/+ CD36IN
p53-/- CD36IN
OD450 values
days
***
J
Hepatocyte-cultured medium
p53+/+ Ctrl
p53-/- Ctrl
p53+/+ PC
p53-/- PC
OD450 values
days
***
K
Hepatocyte-cultured medium
Ctrl
PC
p53 +/+ -/- +/+ -/-
p-p70 S6K
p-p70/p70: 1 3.6 0.9 0.9
p70 S6K
p-S6
p-S6/S6: 1 2.2 0.9 0.9
S6
Actin
HepG2

Supplemental Figure 16. Fatty acids released by HSL contribute to tumor cell proliferation induced by p53 loss. (**A**) Mice administrated of shLacZ or shPCYT1B adenoviruses for 14 days were maintained on a choline-deficient diet. The interstitial fluid of liver tissue containing 10μM CD36 inhibitor (CD36IN, Sulfosuccinimidyl oleate) was used to culture HepG2 cells for different times. The proliferation of HepG2 cells was measured. (**B** and **C**) *p53*$^{+/+}$ and *p53*$^{-/-}$ mice were maintained on a choline-free diet for 4 weeks, with or without oral administration of 300 μL PC (10 mg/ml, **B**) or 100 μL HSL inhibitor HSL-IN-1 (HSLIN, 5 mg/ml, **C**) daily. The interstitial fluid from the liver tissue was used to culture HepG2 cells for different periods of time and cell proliferation was measured. (**D**) Mice administrated with shLacZ or shPCYT1B adenoviruses for 14 days were maintained on a choline-deficient diet. The liver tissue interstitial fluid was used to culture HepG2 cells for 6 hours. mTORC1 activity was determined by western blot analysis of the phosphorylation of S6K and S6. (**E**) Related to **B**. *p53*$^{+/+}$ and *p53*$^{-/-}$ mice were maintained on a choline-free diet for 4 weeks, with or without oral administration of 300 μL PC (10 mg/ml) daily. The liver tissue interstitial fluid was used to culture HepG2 cells for 6 hours. mTORC1 activity in HepG2 cells was determined by western blot analysis of the phosphorylation of S6K and S6. (**F**) Related to **C**. *p53*$^{+/+}$ and *p53*$^{-/-}$ mice were maintained on a choline-free diet for 4 weeks, with or without oral administration of 100 μL HSL inhibitor (5 mg/ml) daily. The liver tissue interstitial fluid was used to culture HepG2 cells for 6 hours. mTORC1 activity in HepG2 cells was determined by western blot analysis of the phosphorylation of S6K and S6. (**G**) Mice administrated of shLacZ or shPCYT1B adenoviruses for 14 days were maintained on a choline-deficient diet. The isolated hepatocytes were cultured in lipid-free medium for 12 hours and then the conditioned medium was used to culture HepG2 cells for another 12 hours. Protein expression was determined by Western blot analysis. (**H**) Mice administrated of shLacZ or shPCYT1B adenoviruses for 14 days were maintained on a choline-deficient diet. The isolated hepatocytes were cultured in lipid-free medium for a further 12 hours. The conditioned medium containing 10μM CD36 inhibitor (CD36IN) was then used to culture HepG2 cells for different times and HepG2 cell proliferation was measured. (**I**) *p53*$^{+/+}$ and *p53*$^{-/-}$ mice were maintained on a choline-free diet for 4 weeks. The isolated hepatocytes were cultured in lipid-free medium for a further 12 hours. The conditioned medium containing 10μM CD36 inhibitor (CD36IN) was then used to culture HepG2 cells for different times and HepG2 cell proliferation was measured. (**J** and **K**) *p53*$^{+/+}$ and *p53*$^{-/-}$ mice were maintained on a choline-free diet for 4 weeks, with or without oral administration of 300 μL PC (10 mg/ml) daily. The isolated hepatocytes were cultured in lipid-free medium for 12 hours. Then the conditioned medium was used to culture HepG2 cells for different times. HepG2 cell proliferation (**J**) and mTORC1 activity (**K**) was measured. All data are the mean±SD. $^*P<0.05$, $^{**}P<0.01$, based on two-way ANOVA (**A**-**C**, **H**-**J**). n.s., not statistical significance.

Fig. S17

A

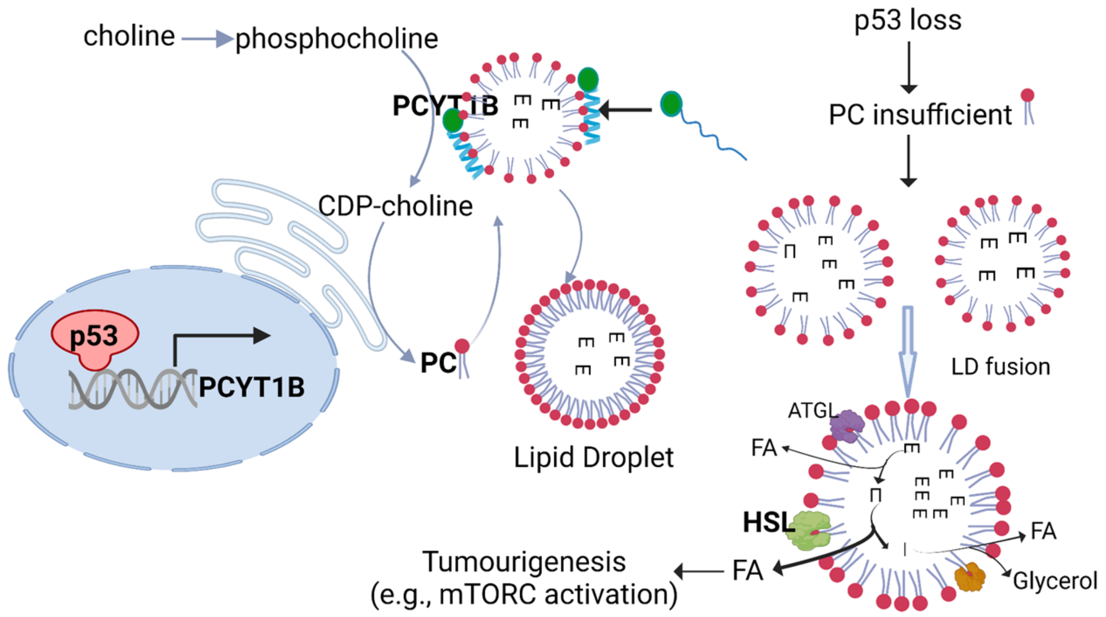

choline
phosphocholine
PCYT1B
CDP-choline
p53
PCYT1B
PC
Lipid Droplet
p53 loss
PC insufficient
LD fusion
ATGL
FA
HSL
FA
Glycerol
FA
Tumourigenesis
(e.g., mTORC activation)

B

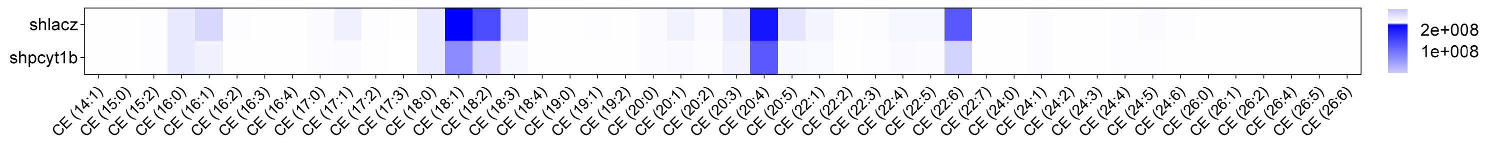

shlacz
shpcyt1b
2e+008
1e+008

C

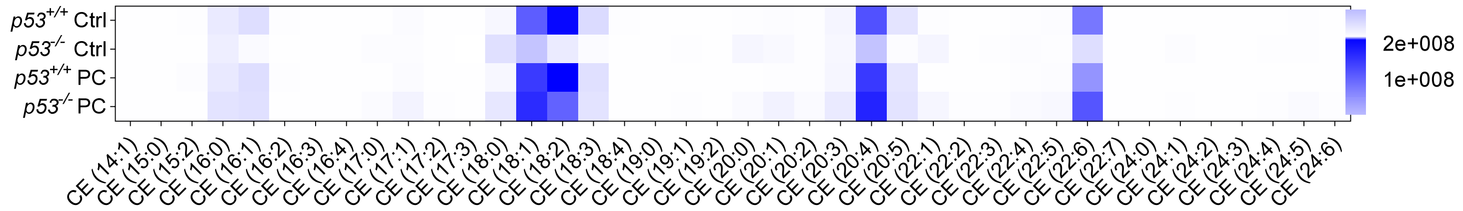

p53+/+ Ctrl
p53-/- Ctrl
p53+/+ PC
p53-/- PC
2e+008
1e+008

Supplemental Figure 17. Loss of p53 or depletion of PCYT1B reduces cholesterol ester levels in mice. (**A**) A working model illustrates how loss of p53 dysregulates the Kennedy pathway of LD growth to fuel tumorigenesis. Through direct upregulation of the expression of PCYT1B p53 unexpectedly functions in activating the Kennedy pathway for de novo PC synthesis and limiting LD coalescence. However, in the absence of p53, PCYT1B, is downregulated and PC synthesis is blocked, leading to LD growth. Moreover, increased surface localization of HSL on LDs caused by p53 loss or defects in the Kennedy pathway can release specific fatty acids as fuel for tumorigenesis. (**B**) Mice receiving shLacZ or shPCYT1B adenoviruses were maintained on a choline-deficient diet for 15 days. Cholesterol ester levels in the interstitial fluid of liver tissue were measured by LC-MS. (**C**) $p53^{+/+}$ and $p53^{-/-}$ mice maintained on a choline-free diet for 3 weeks. Cholesterol ester levels in the interstitial fluid of liver tissue were measured by LC-MS.

Table 1. List of primers

Primers	Sequences (5′ to 3′)	Primers	Sequences (5′ to 3′)
**Human p21-F**	CCGGCGAGGCCGGGATGAG	Human ALDH7A1-F	TTGCGGGAGAAGATCCAAGT
**Human p21-R**	CTTCCTCTTGGAGAAGATC	Human ALDH7A1-R	ACCATACACTGCCACAGGGA
**Human p53-F**	CACGAGCTGCCCCCAGG	Human CHDH-F	TATCACCCTCCATTCAGCACA
**Human p53-R**	TCAGTCGACGTCTGAGT	Human CHDH-R	GAACCCACCTGTTTCCAGATG
**Mouse p21-F**	AACTTCGTCTGGGAGCGC	Human ETNK1-F	GTGCCCAAGCTGAACGTCA
**Mouse p21-R**	TCAGGGTTTTCTCTTGCAGA	Human ETNK1-R	TCACCAGGACTACATCCTCCA
**Mouse p53-F**	ATGTTCCGGGAGCTGAATG	Human ETNK2-F	GCAGATGTCCCTAAGGTAGAGG
**Mouse p53-R**	CCCCACTTTCTTGACCATTG	Human ETNK2-R	GCAGGTCATTGTGACAAAACACC
**Human PCYT1B-F**	ATGCAAGAGCCCTTATGCAAG	Human PCYT2-F	TTCGACCTGTTCCACATCGG
**Human PCYT1B-R**	GCTTCGTATCTCTCGGCTTCATT	Human PCYT2-R	CACGCTCAGAGTCCGTTCAT
**Mouse Pcyt1b-F**	GATTGACTTTGTGGCTCACG	Human EPT1-F	GGCTTTCTGCTGGTCGTATTC
**Mouse Pcyt1b-R**	GCCTTCTGTTCTTTGTGTTGG	Human EPT1-R	GCCCACTACAATCCAAACCCA
**Human CHKB-F**	TGAGGGTTTACCCCGTGAG	Human PISD-F	AGGACCTGCATCACTACCG
**Human CHKB-R**	TCCGCAAGTATGGCGAACATC	Human PISD-R	CCGATGGGCTAATCACGCTG
**Human CHKA-F**	TCAGAGGCGGCCTTAGCAAC	Human PLAAT1-F	CCTGTAGATGGCATTCCTGC
**Human CHKA-R**	CATGGCCTCAGCCCCTTGAA	Human PLAAT1-R	GCCACCTCCTGTCCAATTAC
**Human CHPT1-F**	GCTCGTGCTCATCTCCTACTG	Human PLAAT2-F	AGAAGGAACTGCTGTCTGTGG
**Human CHPT1-R**	CTAAGCGAGCGGCAATTGAA	Human PLAAT2-R	CACTGGTCAGCGAATAAGGC
**Human CEPT1-F**	TACAGCTACAGAGCAGGCAC	Human PLA1A-F	GCCTCGAGATCTCCCTTTTCC
**Human CEPT1-R**	TCCAATCAGGGTTTGTCCCC	Human PLA1A-R	ATCCAAGCGCTCTTCCACAC
**Human PEMT-F**	GGGGTTCGCTGGAACTTTC	Human PLD1-F	CCCAGCGATCCCAAGATACAA
**Human PEMT-R**	GAGCCACTATGTAGGTGAGGG	Human PLD1-R	GACAGCCGGAGAGATACGTCT
**Human PTDSS1-F**	CGTTCACTCGACCTCATCCA	Human PLD2-F	CAGATGGAGTCCGATGAGGTG
**Human PTDSS1-R**	GCTGATAATCCTCTCCCAGGTG	Human PLD2-R	CCGCTGGTATATCTTTCGGTG
**Human PTDSS2-F**	AGGAAACACCTCAGGACACG	Human PLA2G4A-F	TACCAGCACATTATAGTGGAGCA
**Human PTDSS2-R**	GTGGTGGCAACTGGAAGAGTA	Human PLA2G4A-R	GCTGTCAGGGGTTGTAGAGAT
**Human HSL-F**	CTCATGGCTCAACTCCTTCC	Mouse Pcyt1b sh-top	CACCGCCAGGTACAAACAGACACTTAGAGATAAGTGTCTGTTTGTACCTGGC
**Human HSL-R**	GTCAGGTTCTTGAGGGAATCC	Mouse Pcyt1b sh-bottom	AAAAGCCAGGTACAAACAGACACTTATCTCTAAGTGTCTGTTTGTACCTGGC
**Human CIDEB-F**	CAGCGACCTTTCCGTGTCT	Human CD36-F	CTTTGGCTTAATGAGACTGGGAC
**Human CIDEB-R**	GGGTCTCCAATGCTTTGGCT	Human CD36-R	GCAACAAACATCACCACACCA
**Human FABP5-F**	CTGCAACTTTACAGATGGTGC	Human SLC27A1-F	ATGAGGACACAATGGAGCTG
**Human FABP5-R**	GATCCGAGTACAGGTGACATTG	HumanSLC27A1-R	TGACATAGCCATCGAAGCG
**HumanSLC27A2-F**	CGAGAAAAGTTGGTGCTGTTG	HumanSLC27A3-F	ATCAACTACACAGGACAGCG
**HumanSLC27A2-R**	TGGGAACTCTGACGCAATATC	HumanSLC27A3-R	CATAGCGAATCAAGGAGAAGGG
**HumanSLC27A5-F**	TGATGGGACTTGTCGTTGG	Mouse Cd36-F	ATGGGCTGTGATCGGAACTG
**HumanSLC27A5-R**	CCAGAAGCAGGAAGTAGAGAAC	Mouse Cd36-R	GTCTTCCCAATAAGCATGTCTCC
**Mouse Cideb-F**	CAATGGCCTGCTAAGGTCAGT	Mouse Bscl2-F	TGGGGCAAGAGAGACATGC
**Mouse Cideb-R**	GATCACAGACACGGAAGGGTC	Mouse Bscl2-R	TCTTCCACAGGGACGATACCC
**Mouse Slc27a1-F**	CCCATCTTCCTGCGTCTTC	Mouse Slc27a2-F	ACATCTACTTCAACAGCGGAG

**Mouse Slc27a1-R**	GTACATAGCGTCCCTGCTTC	Mouse Slc27a2-R	ACAAAATCTACCAGTCCCACG
**Mouse Slc27a3-F**	ATCTACCTCGCACTCTCACT	Mouse Slc27a4-F	CTGGAAGATGCCCCGAAG
**Mouse Slc27a3-R**	ACTGTCACCCTGTGTTTCTG	Mouse Slc27a4-R	GCCCGATGTGTAGATGTAGAAG
**Human BIP-F**	CTGCCATGGTTCTCACTAAAATG	Human XBP1s-F	GCCCTGGTTGCTGAAGAG
**Human BIP-R**	TTAGGCCAGCAATAGTTCCAG	Human XBP1s-R	AGTCAATACCGCCAGAATCC
**Mouse BIP-F**	ACTTGGGGACCACCTATTCCT	Human XBP1u-F	TGGATTCTGGCGGTATTGAC
**Mouse BIP-R**	GTTGCCCTGATCGTTGGCTA	Human XBP1u-R	TCCTTCTGGGTAGACCTCTG
