# Supplementary material for: p53 suppresses lipid droplet–fueled tumorigenesis through phosphatidylcholine
Source: J Clin Invest. 2024 Jan 9;134(4):e171788. doi: 10.1172/JCI171788 (PMC10866454; doi:10.1172/JCI171788)

Figure 1**E**

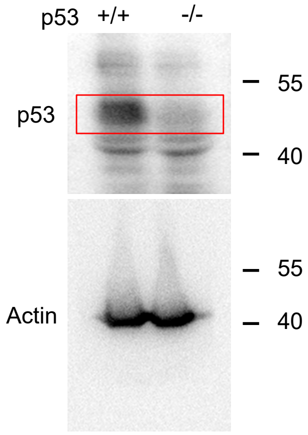

Mouse liver tissue

Figure 2F

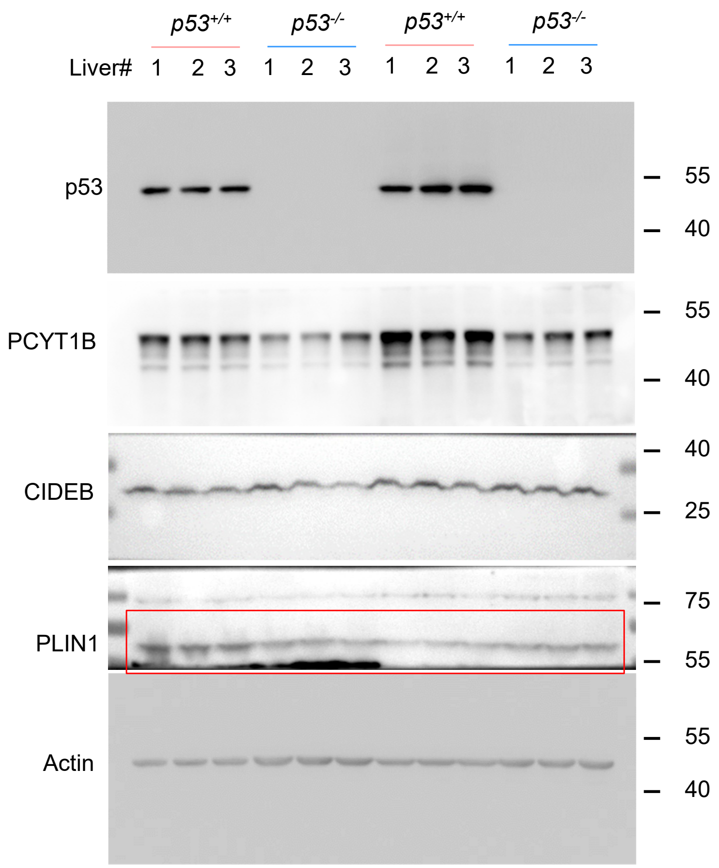

Figure 2G

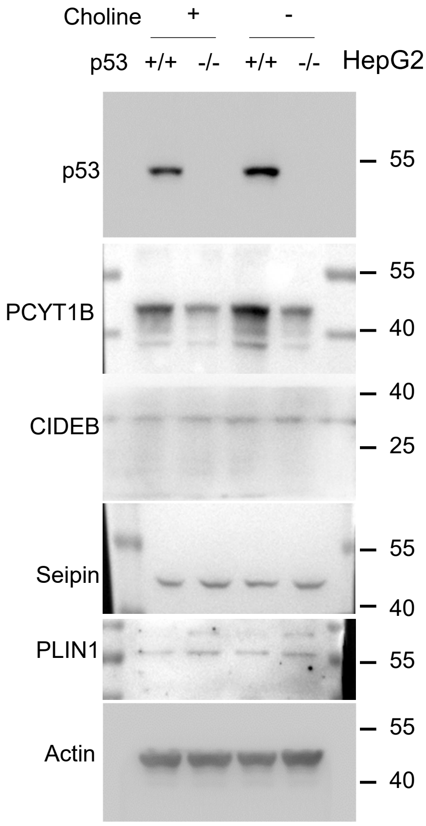

Figure 3**A**

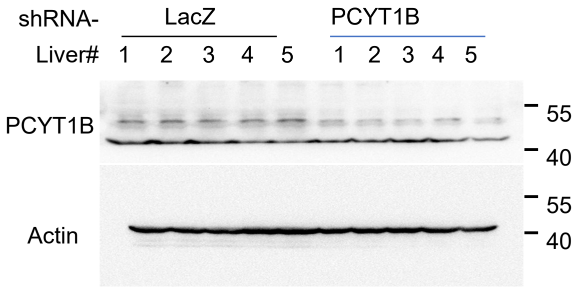

Figure 3**F**

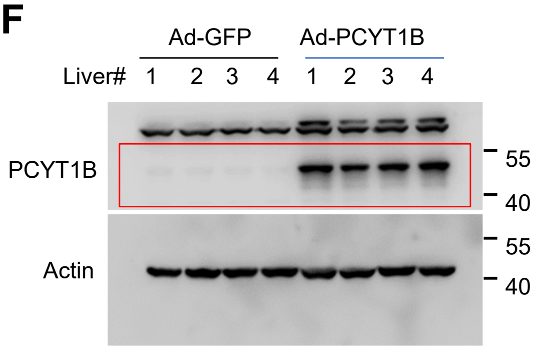

Figure 3**K**

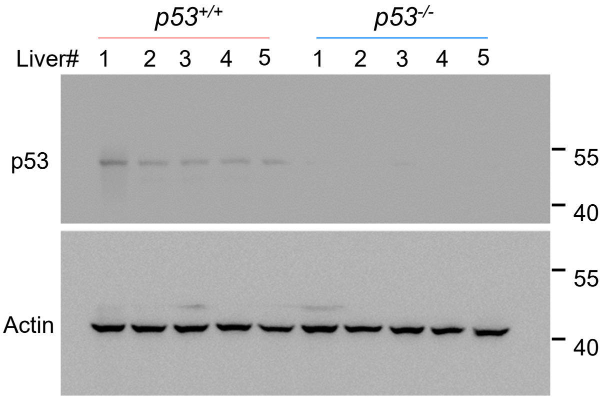

Figure 4**B**

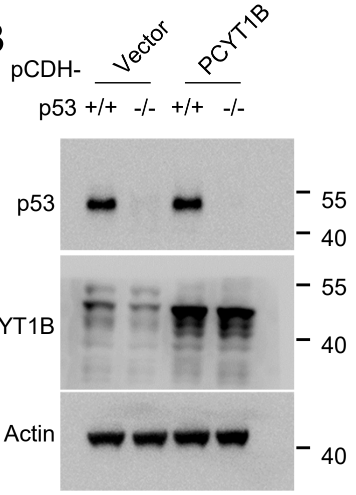

Figure 4**I**

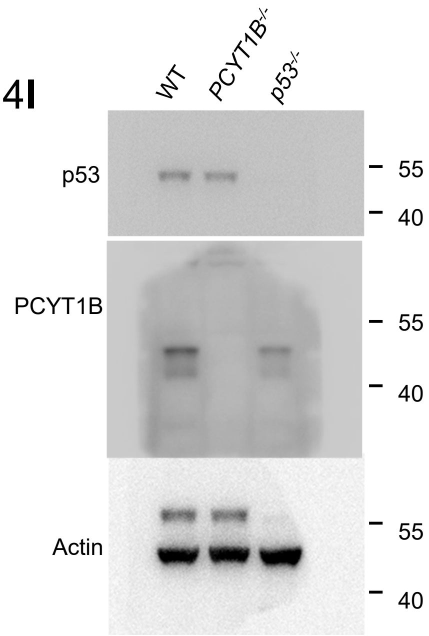

Figure 5**D**

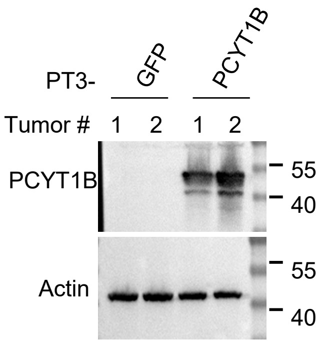

Figure 6J

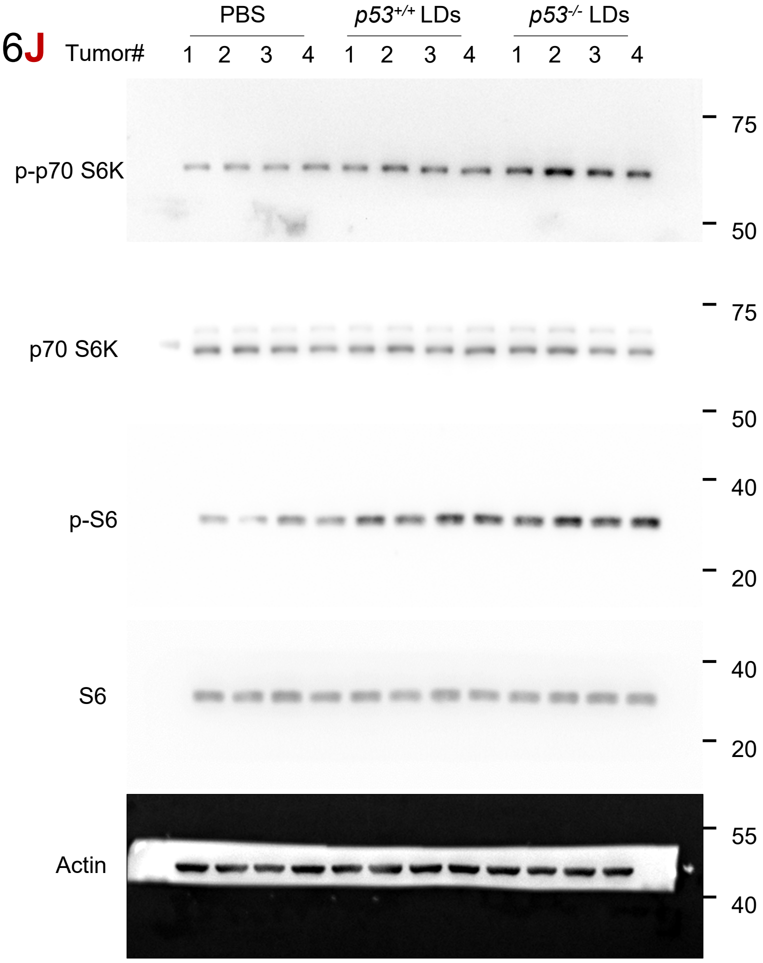

PBS
p53+/+ LDs
p53-/- LDs
Tumor#
1 2 3 4 1 2 3 4 1 2 3 4
p-p70 S6K
p70 S6K
p-S6
S6
Actin
75
50
75
50
40
20
40
20
55
40

Figure 7C

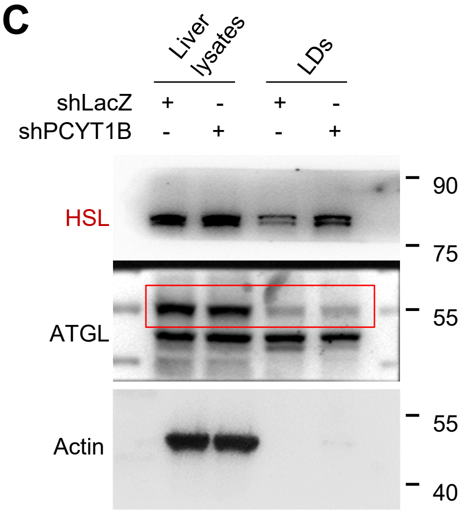

Figure 7D

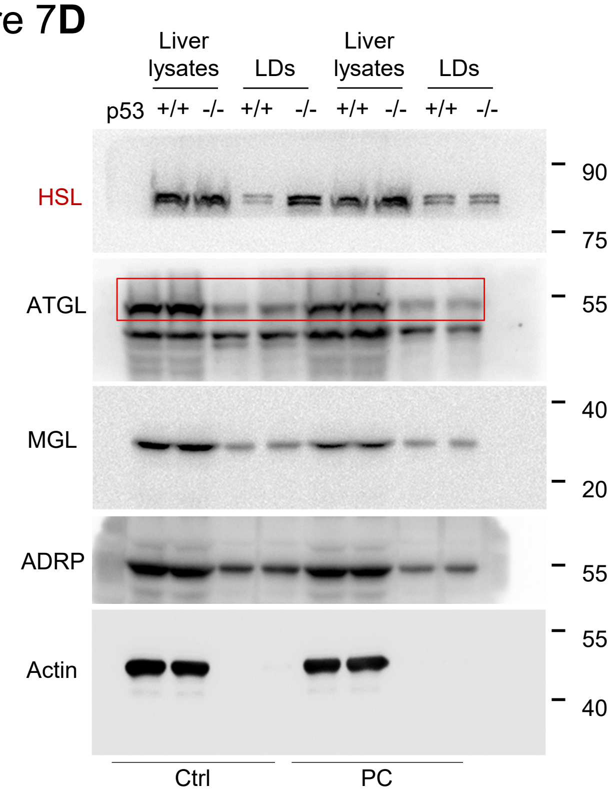

Figure 7**G**

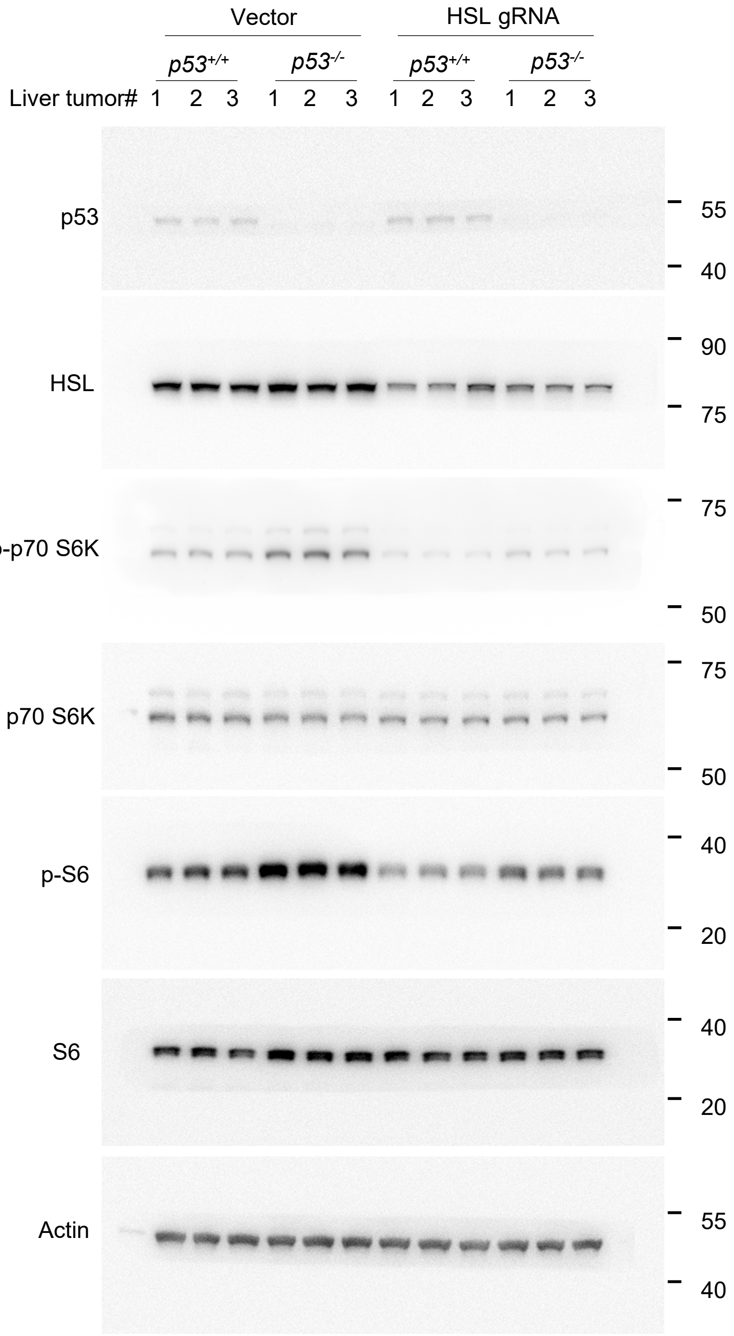

Figure 7**J**

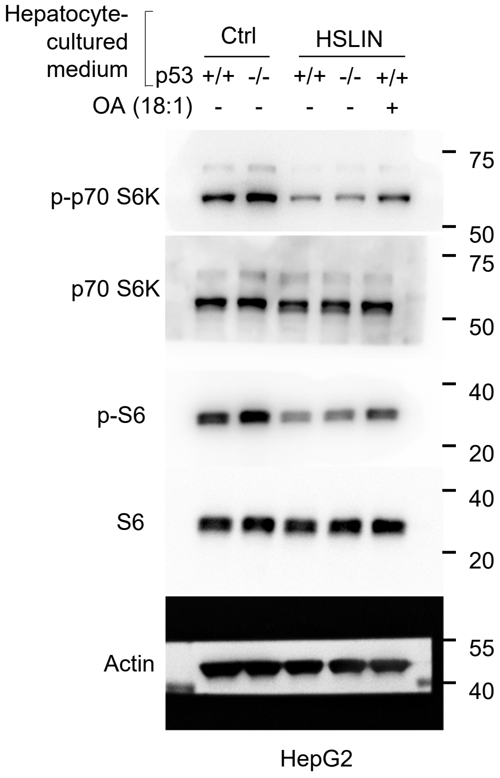

Fig. S1A

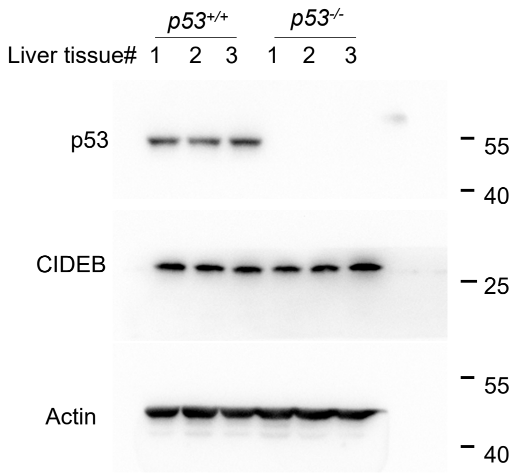

Fig. S1B

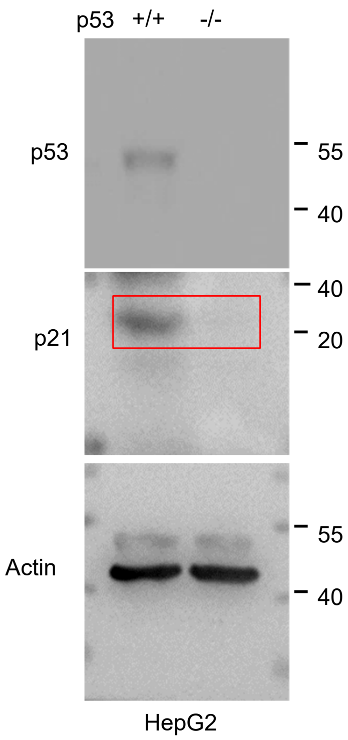

Fig. S3A

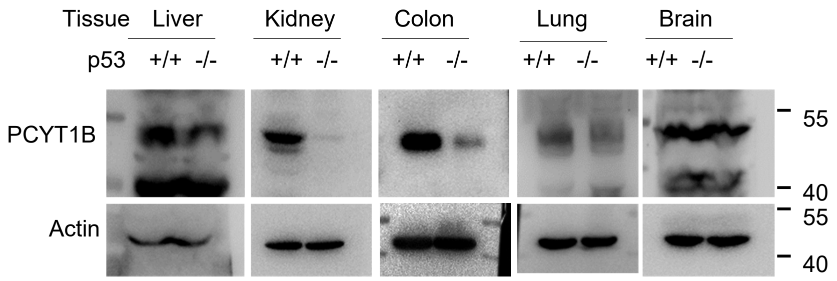

Fig. S3D

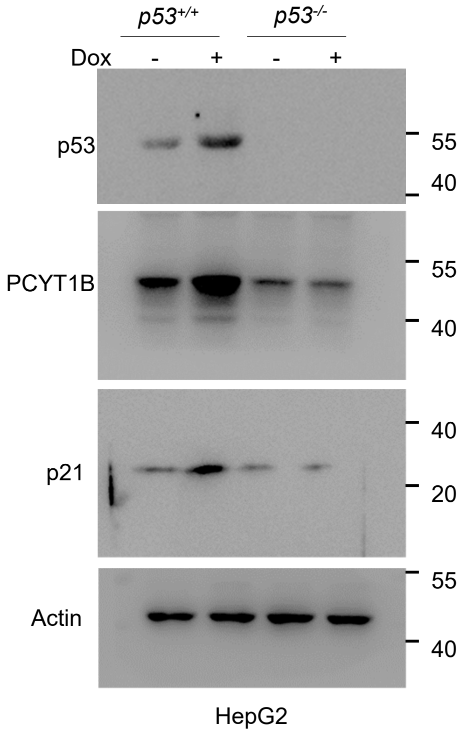

Fig. S3E

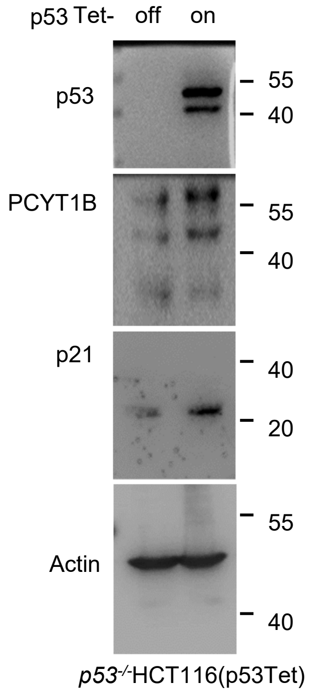

Fig. S4

Fig. S4**A**

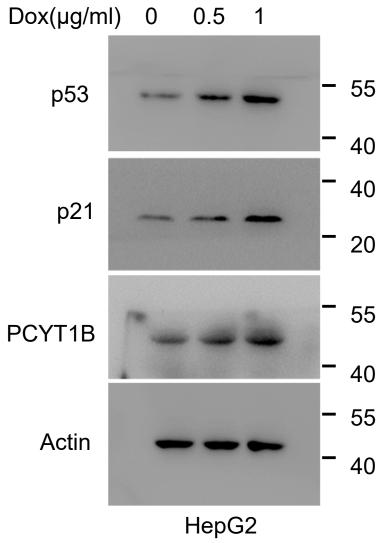

Fig. S4**B**

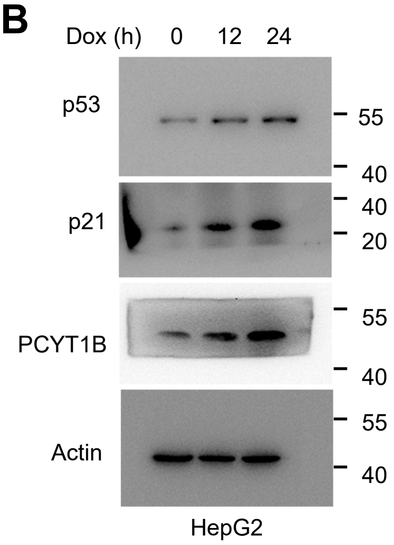

Fig. S4**C**

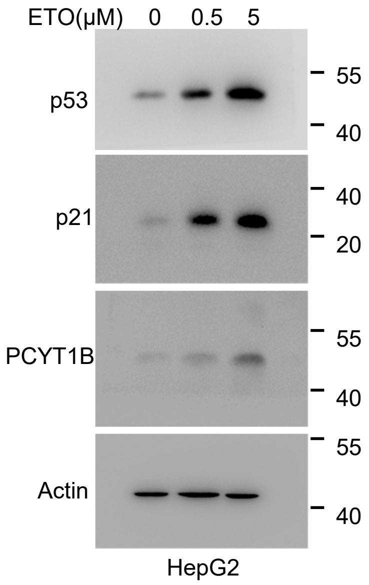

Fig. S4**D**

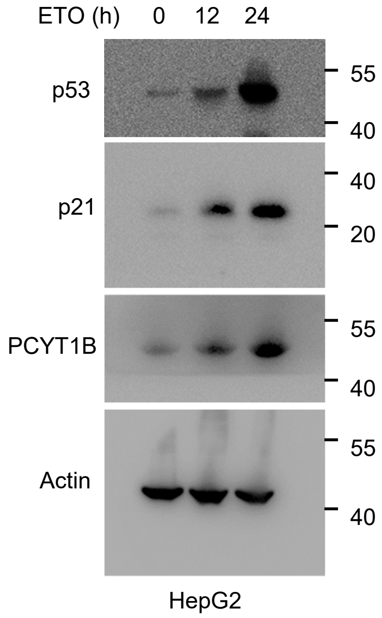

Fig. S4**E**

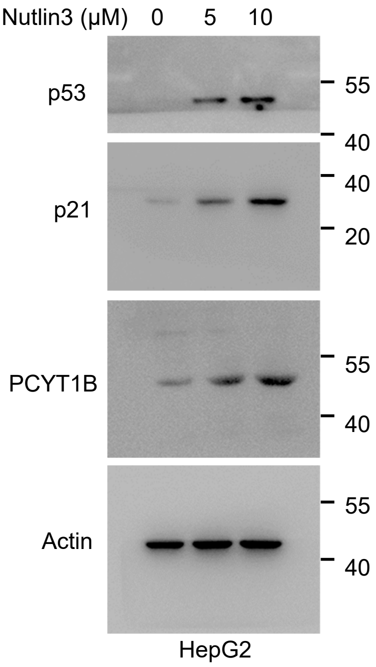

Fig. S4**F**

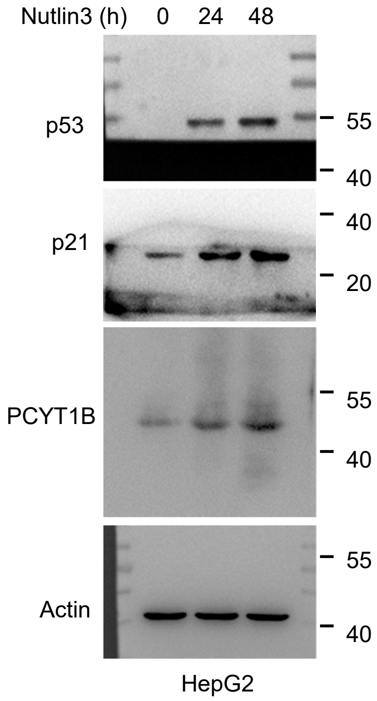

Fig. S4**G**

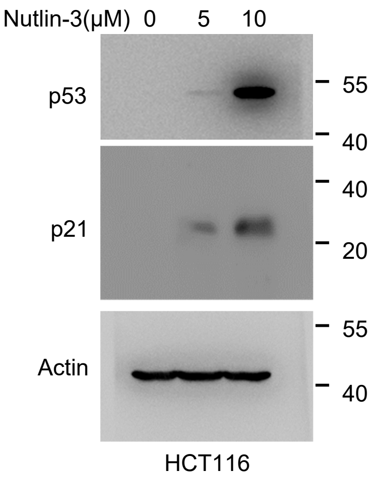

Fig. S4**H**

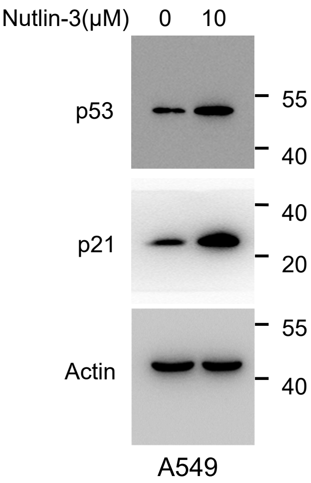

Fig. S4**I**

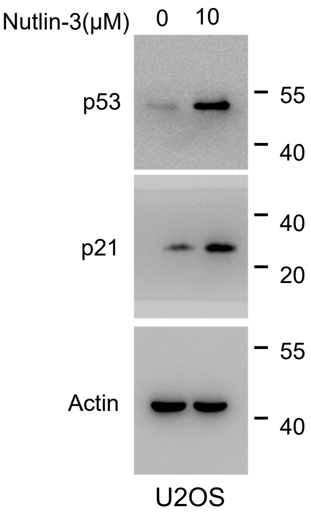

Fig. S5

Fig. S5**A**

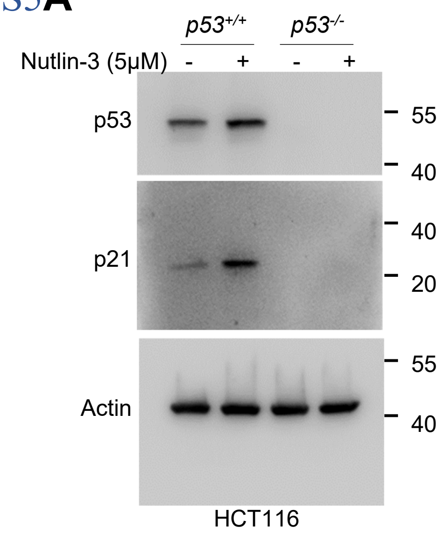

Fig. S5**B**

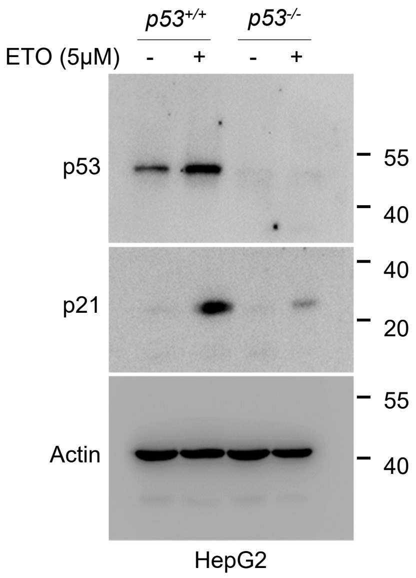

Fig. S5**C**

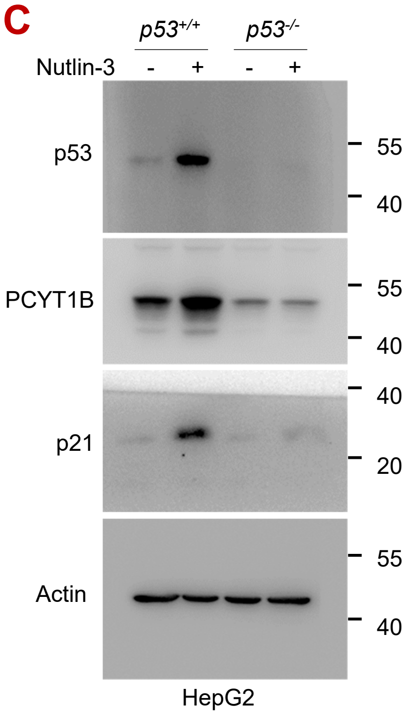

Fig. S5**H**

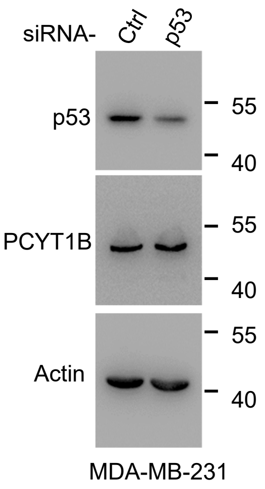

Fig. S5**I**

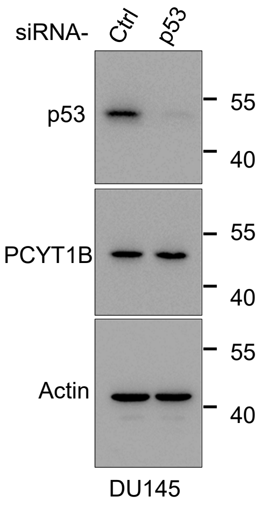

Fig. S5**J**

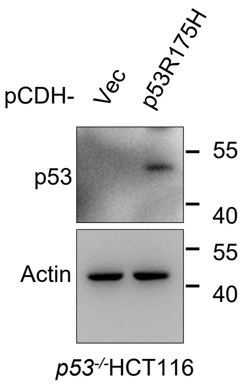

Fig. S5**K**

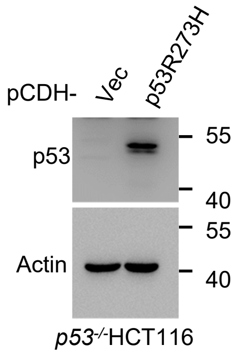

Fig. S9**E**

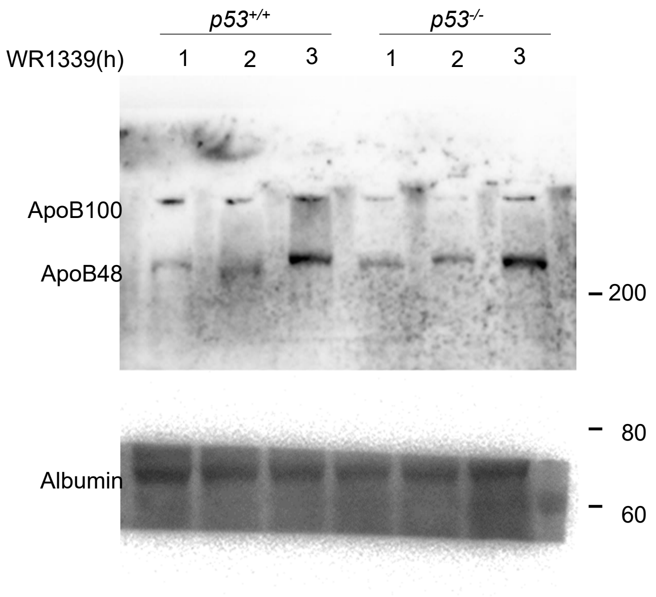

Fig. S10**B**

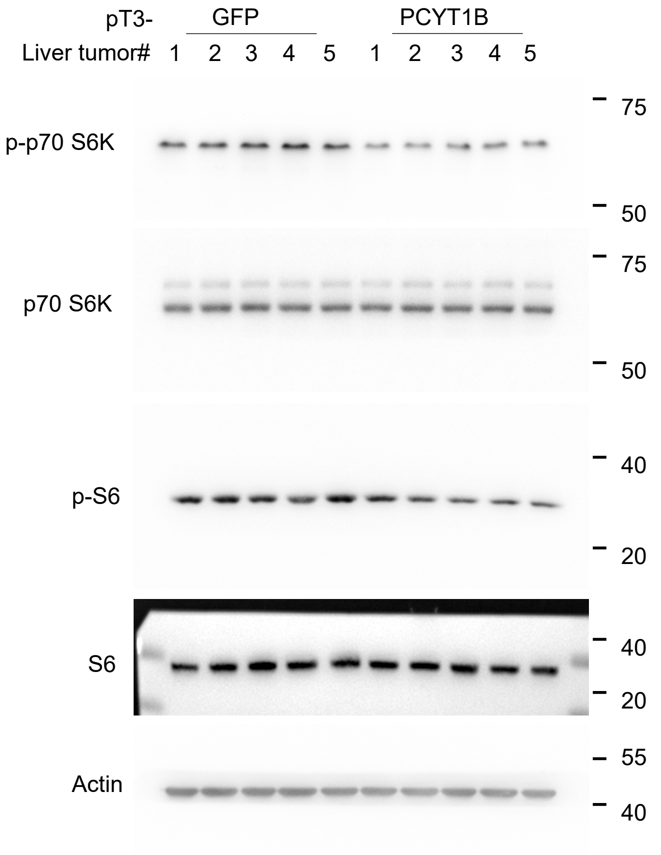

Fig. S10**G**

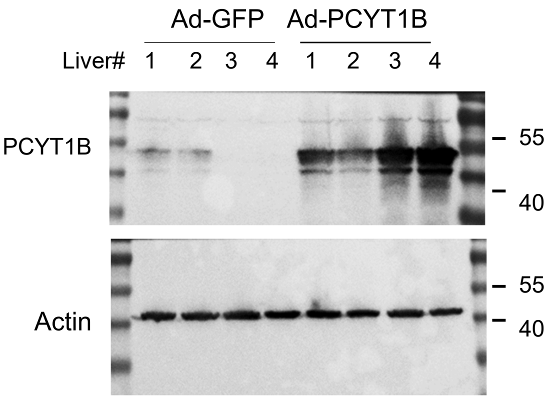

Fig. S10**K**

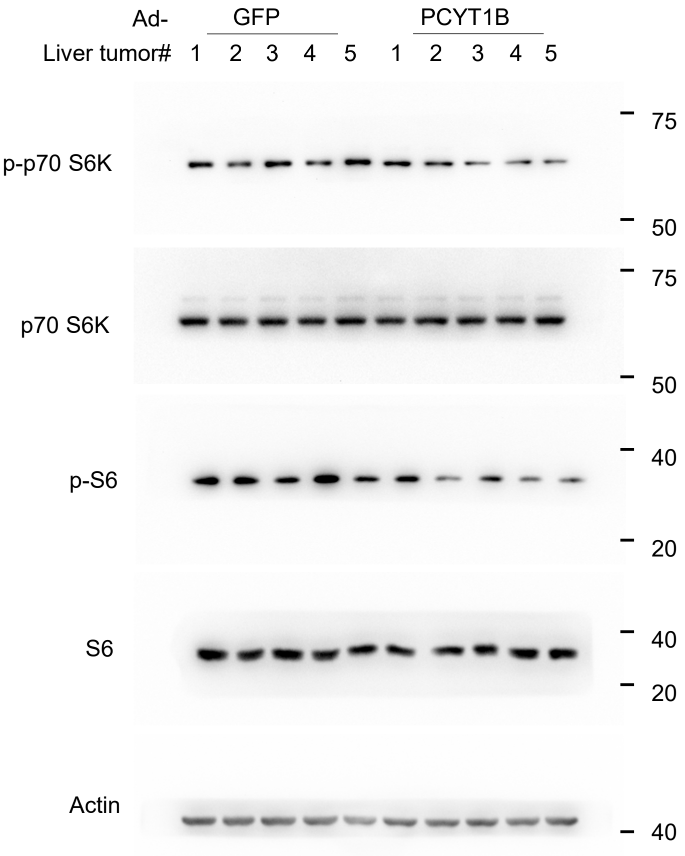

Fig. S11**B**

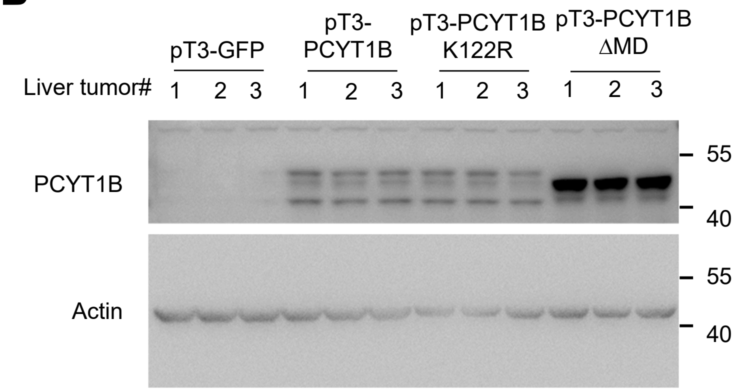
pT3-GFP
pT3-PCYT1B
pT3-PCYT1B K122R
pT3-PCYT1B ΔMD
Liver tumor#
1 2 3 1 2 3 1 2 3 1 2 3
PCYT1B
Actin
55
40
55
40

Fig. S13**F**

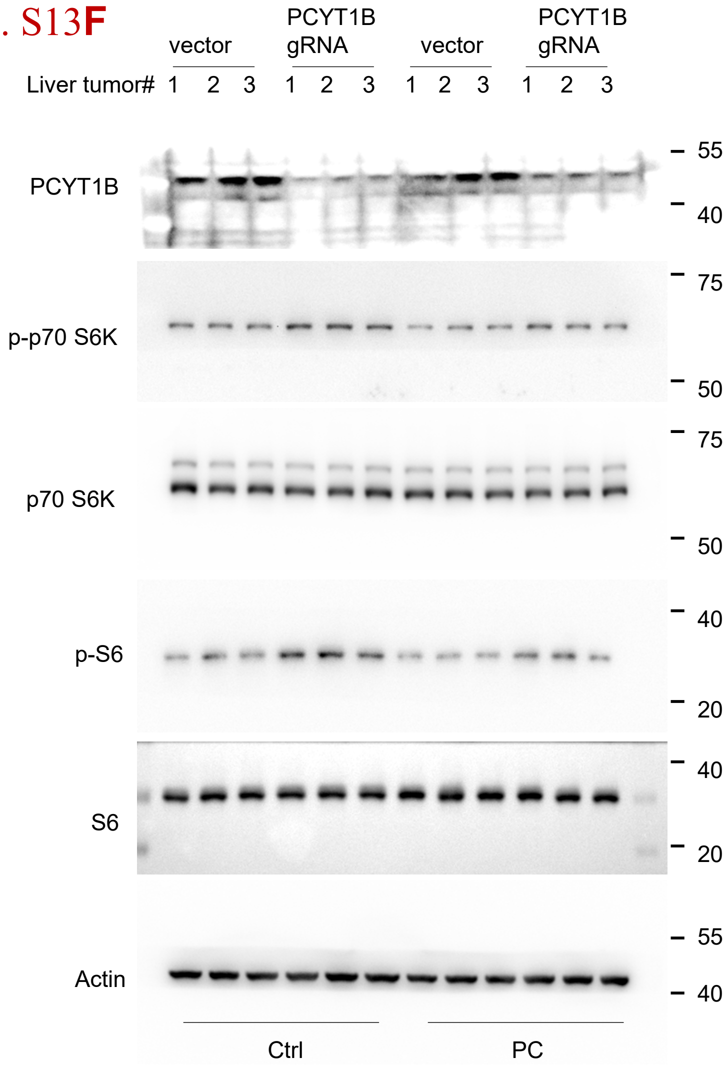
vector
PCYT1B gRNA
vector
PCYT1B gRNA
Liver tumor# 1 2 3 1 2 3 1 2 3 1 2 3
PCYT1B
p-p70 S6K
p70 S6K
p-S6
S6
Actin
55
40
75
50
75
50
40
20
40
20
55
40
Ctrl
PC

Fig. S13**H**

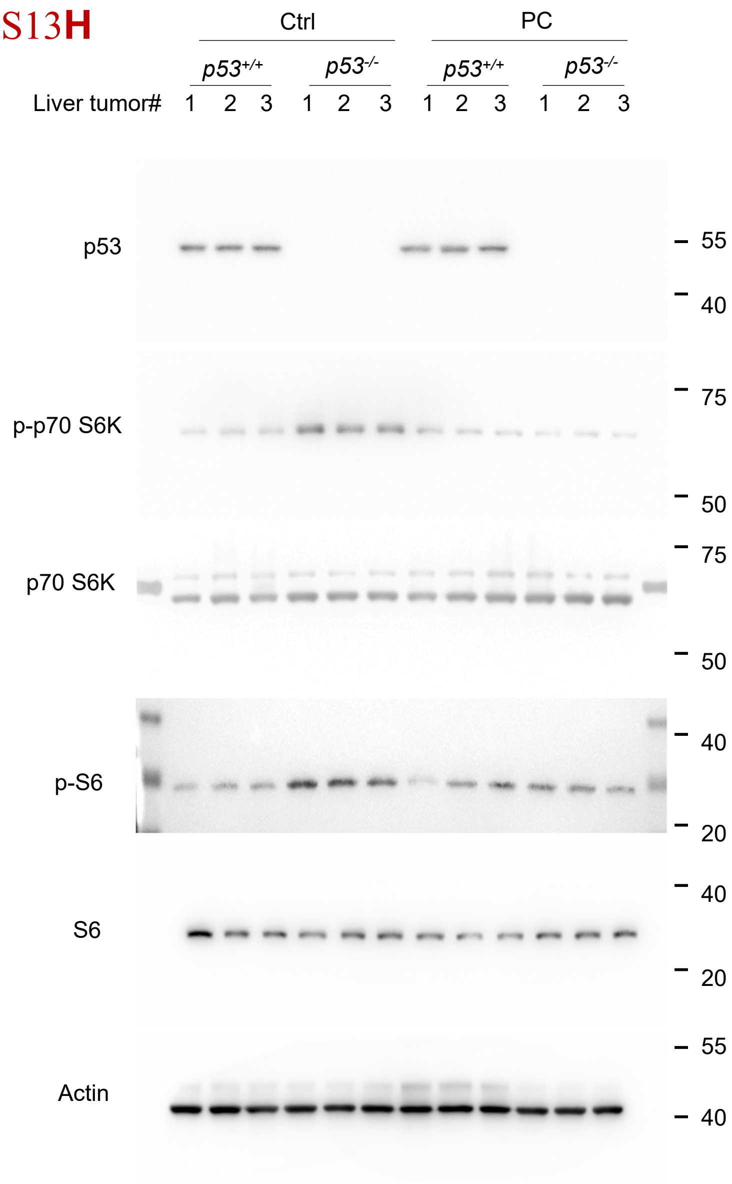

Ctrl
PC
p53+/+
p53-/-
p53+/+
p53-/-
Liver tumor# 1 2 3 1 2 3 1 2 3 1 2 3
p53
55
40
p-p70 S6K
75
50
p70 S6K
75
50
p-S6
40
20
S6
40
20
Actin
55
40

Fig. S14**A**

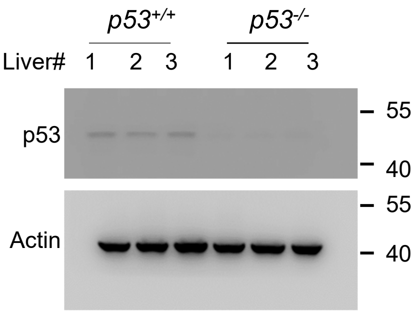

Fig. S14**B**

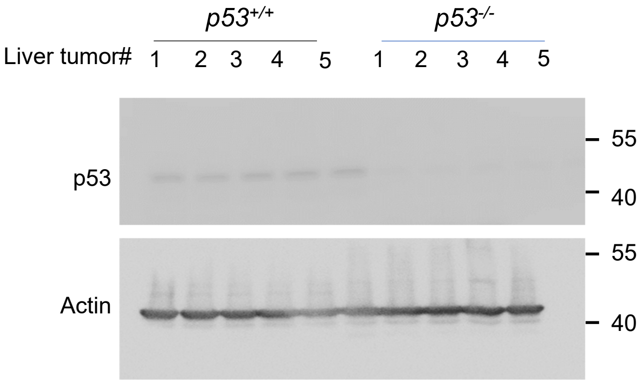

Fig. S15**D**

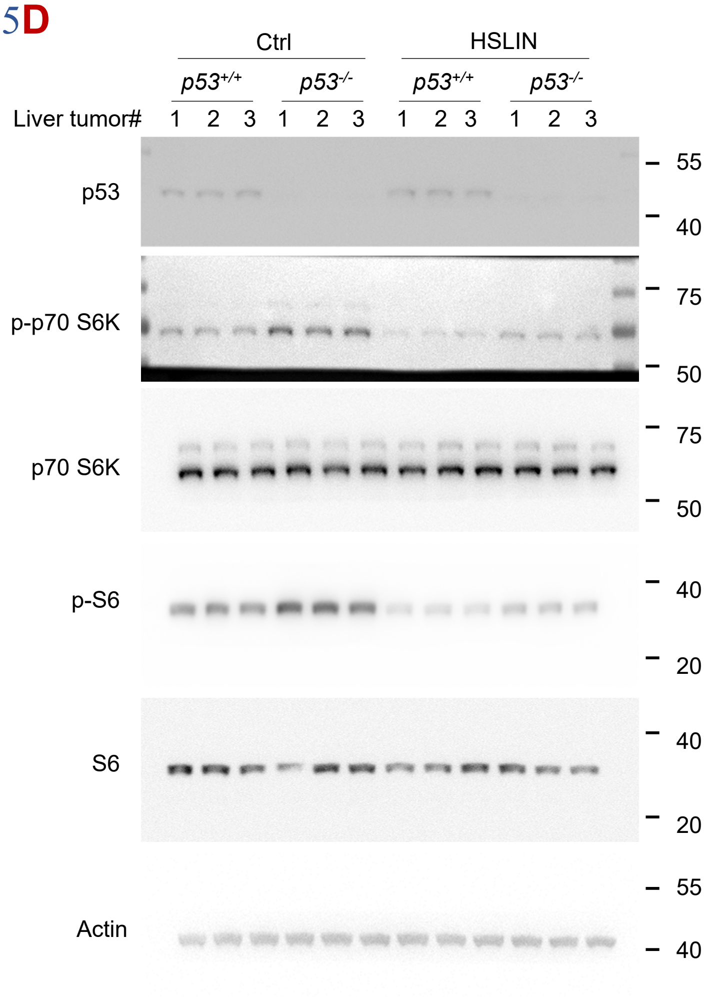
Ctrl
HSLIN
p53+/+
p53-/-
p53+/+
p53-/-
Liver tumor#
1 2 3 1 2 3 1 2 3 1 2 3
p53
55
40
p-p70 S6K
75
50
p70 S6K
75
50
p-S6
40
20
S6
40
20
Actin
55
40

Fig. S16**D**

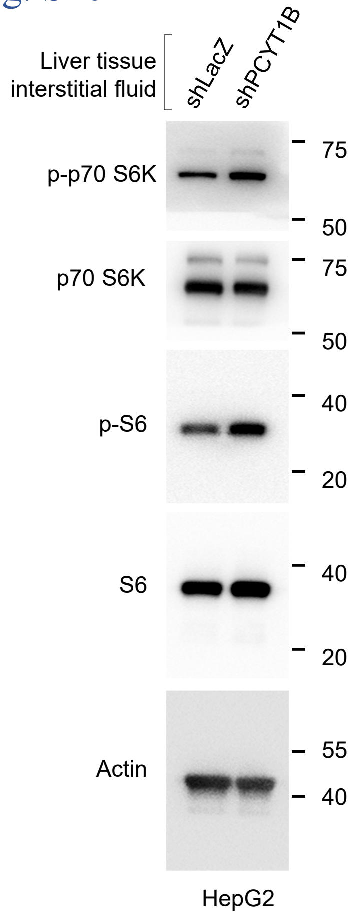

Fig. S16**E**

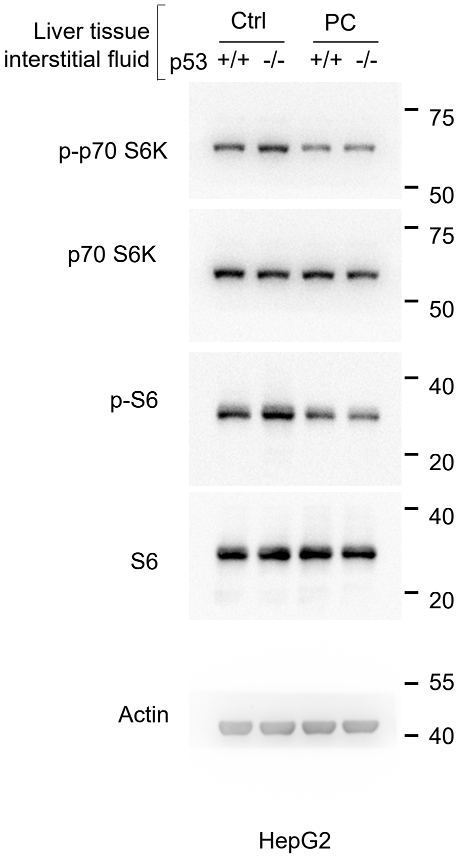

Fig. S16**F**

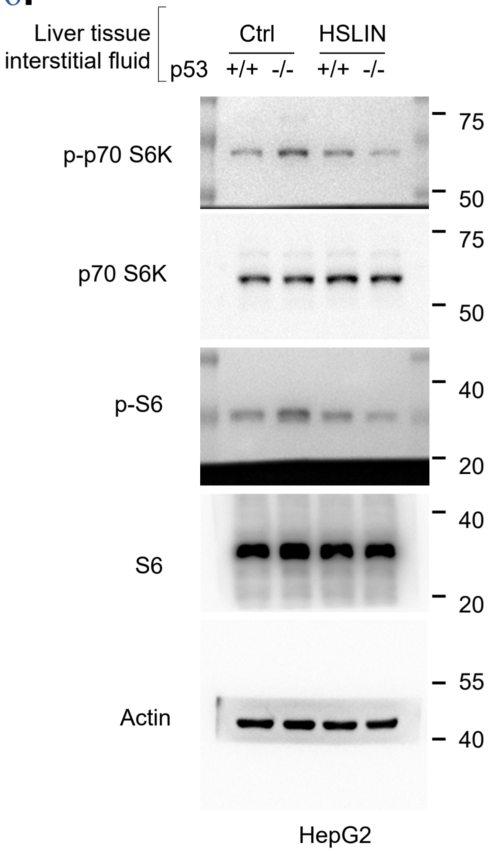

Fig. S16**G**

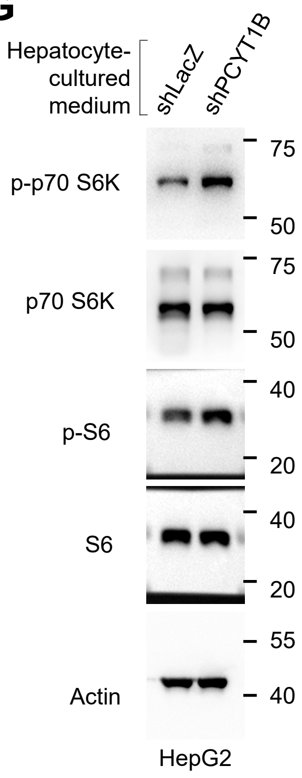

Supplement: Unedited blot and gel images [file jci-134-171788-s027.pdf]
